# Influence of rice varieties, organic manure and water management on greenhouse gas emissions from paddy rice soils

**DOI:** 10.1371/journal.pone.0253755

**Published:** 2021-06-30

**Authors:** Ei Phyu Win, Kyaw Kyaw Win, Sonoko D. Bellingrath-Kimura, Aung Zaw Oo

**Affiliations:** 1 Department of Agronomy, Yezin Agricultural University, Yezin, Myanmar; 2 Institute of Land Use Systems, Faculty of Life Science, Humboldt-Universität zu Berlin, Berlin, Germany; 3 Institute for Agro-Environmental Science, National Agriculture and Food Research Organization, Tsukuba, Ibaraki, Japan; Emory University, UNITED STATES

## Abstract

The study is focused on impact of manure application, rice varieties and water management on greenhouse gas (GHG) emissions from paddy rice soil in pot experiment. The objectives of this study were a) to assess the effect of different types of manure amendments and rice varieties on greenhouse gas emissions and b) to determine the optimum manure application rate to increase rice yield while mitigating GHG emissions under alternate wetting and drying irrigation in paddy rice production. The first pot experiment was conducted at the Department of Agronomy, Yezin Agricultural University, Myanmar, in the wet season from June to October 2016. Two different organic manures (compost and cow dung) and control (no manure), and two rice varieties; Manawthukha (135 days) and IR-50 (115 days), were tested. The results showed that cumulative CH_4_ emission from Manawthukha (1.084 g CH_4_ kg^-1^ soil) was significantly higher than that from IR-50 (0.683 g CH_4_ kg^-1^ soil) (P<0.0046) with yield increase (P<0.0164) because of the longer growth duration of the former. In contrast, higher cumulative nitrous oxide emissions were found for IR-50 (2.644 mg N_2_O kg^-1^ soil) than for Manawthukha (2.585 mg N_2_O kg^-1^ soil). However, IR-50 showed less global warming potential (GWP) than Manawthukha (P<0.0050). Although not significant, the numerically lowest CH_4_ and N_2_O emissions were observed in the cow dung manure treatment (0.808 g CH_4_ kg^-1^ soil, 2.135 mg N_2_O kg^-1^ soil) compared to those of the control and compost. To determine the effect of water management and organic manures on greenhouse gas emissions, second pot experiments were conducted in Madaya township during the dry and wet seasons from February to October 2017. Two water management practices {continuous flooding (CF) and alternate wetting and drying (AWD)} and four cow dung manure rates {(1) 0 (2) 2.5 t ha^-1^ (3) 5 t ha^-1^ (4) 7.5 t ha^-1^} were tested. The different cow dung manure rates did not significantly affect grain yield or greenhouse gas emissions in this experiment. Across the manure treatments, AWD irrigation significantly reduced CH_4_ emissions by 70% during the dry season and 66% during the wet season. Although a relative increase in N_2_O emissions under AWD was observed in both rice seasons, the global warming potential was significantly reduced in AWD compared to CF in both seasons (P<0.0002, P<0.0000) according to reduced emission in CH_4_. Therefore, AWD is the effective mitigation practice for reducing GWP without compromising rice yield while manure amendment had no significant effect on GHG emission from paddy rice field. Besides, AWD saved water about 10% in dry season and 19% in wet season.

## Introduction

Developing new strategies is necessary to achieve the dual goals of ensuring food security and protecting natural resources and the environment through reduced greenhouse gas (GHG) emissions [1, 2]. It is estimated that nitrous oxide (N_2_O) and methane (CH_4_) emissions may increase by 35–60% and 60%, respectively, by 2030 [3]. Flooded rice soils are an important source of global CH_4_ emissions [4, 5], and rice-based cropping systems can emit substantial amounts of N_2_O [6] during the rice season itself [7].

Rice is one of the most important cereal grains and staple food crops globally and is particularly important in Asia [8]. Rice paddies contribute to the emission of the two most important GHGs; methane and nitrous oxide. IPCC [9] reported that rice fields contribute about 30% and 11% of global agricultural CH_4_ and N_2_O emissions, respectively. With a linearly increasing rate of 0.26% per year during the recent few decades, the atmospheric N_2_O concentration has increased by 18% compared to the preindustrial level. Methane and nitrous oxide have long atmospheric lifetimes of 12 and 114 years, respectively, and account for 20% and 7%, respectively, of global radiative forcing [10]. The high global warming potential (GWP) of CH_4_ and N_2_O, 34 and 298 times that of CO_2_ at a 100-year time horizon, makes them major contributors to climate change [11]. In recent years, suitable management practices have been developed for achieving both improvement in rice yields and mitigation of GHG emissions, which include the development of new rice varieties [12], the application of manure such as cow dung [13], the selection of appropriate cultivation methods [14] and the timing of drainage [15].

The magnitude of CH_4_ emissions from rice plants is regulated by complex and dynamic interactions among the plants, environment, and microorganisms [16]. Methane produced in flooded rice soils is emitted to the atmosphere by molecular diffusion, ebullition or plant-mediated transport. Approximately 80–90% of the total CH_4_ flux is emitted to the atmosphere from the rhizosphere via the rice plant [17]. An increase in plant biomass [18] and tiller number [19] enhanced CH_4_ oxidization activity by enlarging the volume of aerenchyma and enhancing O_2_ transport from the atmosphere to the rhizosphere. Ma et al. [20] revealed that a hybrid rice variety with 50–60% higher shoot biomass emitted less CH_4_ than an indica rice variety, possibly due to higher CH_4_ oxidization activity.

Nitrous oxide is produced as a by-product of nitrification, denitrification, nitrifier denitrification, etc. and moisture content is a key factor governing N_2_O production in soils [21]. Ciarlo et al. [22] found that denitrification is dominant pathway for N_2_O emission when the water-filled pore space in soils is high (80%), and if the soils were saturated than this level, most of N_2_O would be reduced to nitrogen.

Selecting a rice variety that has high productivity and low GHG emissions is crucial for improving crop yield and mitigating climate change; however, research examining the effects of rice varieties has mostly focused on CH_4_ flux so far [12, 23, 24], with little focus on N_2_O flux [25]. Many studies reported that the effect of rice varieties on methane emissions is mostly related to rice growth performance, i.e., the number of plant tillers and above- and belowground biomass, root exudates and root arenchyma [26–30]. Although significant positive relationship have been found between rice biomass and methane fluxes [31, 32], a comparison of rice varieties has produced different results [33, 34].

Organic residue amendments have been practised to improve soil fertility in paddy production. The organic matter in paddy fields originates from both direct by-products of rice production (such as sloughed-off root cells and root exudates) and added materials (manures and previous crop residues). The addition of organic carbon to the soil, whether it comes from the disposal of crop residues or as organic fertilizer, appears to be the most important factor in methane production [32]. Waterlogged conditions are ideal for the decomposition of organic matter in paddy fields. The methane production from rice soil can be increased by addition of cow manure as a source of organic material [35]. Nitrous oxide emissions from applied fertilizer and manures can vary with different environmental factors (e.g., climate and soil conditions), crop factors (e.g., crop type and crop residues), and management practices (e.g., type of manure and fertilizer, application rate, time of application) [36].

Although there are wide range of factors influencing methane emission from paddy soil [37–39], water management and organic amendment are the two main drivers of methane production. Under anaerobic soil conditions, methanogens (methane-producing bacteria) produce methane by oxidation of organic matter during anaerobic respiration. Flooded rice soils are known to have strong denitrification activity emitting some amount of N_2_O from rice soils. However, it is also guided by the water management conditions of the rice field. Nitrate and nitrite in rice soils is limited due to submerged conditions. The oxygen supply due to decomposition of organic matter, roots, and also through vascular transport via tillers may help in production of nitrate in rice soils. Sometimes due to prolonged submergence of the rice fields, the soil nitrate and nitrite N (available due to mineralization of organic matter) is completely reduced to N_2_ gas thereby resulting in low N_2_O emission. This varies with the prevailing conditions of the rice field. Although minimal N_2_O emissions are likely from flooded soils, some off-site (indirect) N_2_O emissions are likely from irrigated rice production due to the addition of nitrogen fertilizer to fields [40].

Myanmar ranks the sixth largest production for rice in the world. Rice is the country’s most important crop and is grown on 7.3 million ha [41]. The conventional rice production method commonly used by the farmers in Myanmar includes transplanting old seedlings (30–45) under continuous flooding conditions and the intensive use of organic fertilizers such as manure or compost. However, there is very limited information on methane emissions from the flooded rice fields of Myanmar, although more than half of the cultivated area is under to rice production [13, 42]. Thus, the objectives of this study were a) to assess the effect of different types of manure amendments and rice varieties on greenhouse gas emissions and b) to determine the optimum manure application rate to increase rice yield while mitigating GHG emissions under alternate wetting and drying irrigation in paddy rice production.

## Materials and methods

**Fig 1:**
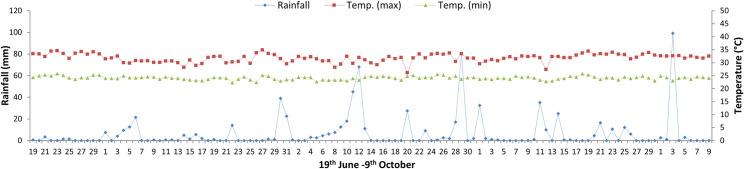
Daily rainfall, maximum and minimum temperatures in Yezin Agricultural University, Myanmar during wet season, 2016.

The first pot experiment was conducted at open field, Department of Agronomy, Yezin Agricultural University (19° 45’N and 96° 6’E), Myanmar, during the wet season (June–October), 2016 to study the local production potential of GHG emission in this area. A two-factor factorial experiment with completely randomized design was used with 3 replications. The factor A was assigned into two categories of organic manure (compost and cow dung) and control (no manure). The compost is collected from straw compost making process and stored for ten months. The cow dung is resulted from farmer traditional heap method for ten months. The amount of organic manure for cow dung and compost treatments was based on the nitrogen content of the organic manure analysis. The recommended chemical fertilizer amounts are 60 kg N ha^-1^, 30 kg P_2_O_5_ ha^-1^, and 20 kg K_2_O ha^-1^ for all treatments. The recommended amounts of nitrogen fertilizer of compost and cow dung treatments were replaced by compost and cow dung manures which was based on same amount of carbon content. Therefore, 3 t ha^-1^ of compost was applied to compost treatment, and 3.15 ton cow dung + chemical fertilizer (22.15 kg N ha^-1^) was applied to cow dung treatment to get the recommended amount of nitrogen fertilizer based on their nitrogen analysis. The control treatment received only recommended amount of chemical nitrogen fertilizer. The factor B was two types of rice varieties: Manawthukha (135 days) and IR-50 (115 days). The two rice varieties were grown in a concrete pot (52.5 cm in diameter and 45 cm in height). The soil was collected from a lowland irrigated rice field. Twenty-one-day-old seedlings were transplanted with two seedlings per pot. Compost and cow dung manures were broadcasted at 14 days before transplanting to avoid transplanting shock due to the manure decomposition process. The soil was analysed for pH (1:5 soil: water suspension), electrical conductivity (1:5 soil: water suspension), total N% (Kjaldehl distillation method), organic matter% (Tyurin’s method), calcium chloride extractable SO_4_-S (Turbidity method) and Texture (pipette method). The manures were analysed for total N% (Kjaldehl distillation method), total P% (Molybivanado phosphoric acid method), total K% (Wet digestion with HNO3: HCLO4 (4:1), total S% (Turbidity method) and organic carbon % (Tyurin’s method). Table 1 shows the physiochemical properties of the soil and manures. The recommended amount of T-super (30 kg P_2_O_5_ ha^-1^) was applied as basal fertilizer, and the recommended amount of potash (20 kg K_2_O ha^-1^) was applied with two split applications (as basal fertilizer and at the panicle initiation stage) to all treated pots. The water level was maintained at 5 cm throughout the rice growing period except during the drying period before harvest. During the rice growing season, weather data were recorded at the Department of Agronomy and are shown in Fig 1. The average minimum and maximum temperatures during the rice growing season (wet season) were 23.9 and 31.8°C, respectively, with 739 mm of rainfall.

**Table 1:** Physiochemical properties of experimental soil and organic manures used in the first pot experiment.

**Item**	**Total N%**	**Total P%**	**Total K%**	**O.C%**				
Cow dung	1.2	1.0	2.1	23.3				
Compost	2.0	2.9	1.9	24.5				
**Item**	**pH**	**EC (dS/m)**	**Total N%**	**OM%**	**SO4-S (mg/kg)**	**Texture %**		
						**Sand**	**Silt**	**Clay**
Soil	6.7	0.4	0.2	2.7	12	79.74	13.28	6.98

The second pot experiment was conducted in a farmer’s field, Madaya Township (22° 13’ 0" N and 96° 7’ 0" E), Myanmar, during the dry and wet seasons (February–October 2017) to assess the effect of cow dung manure and water management on greenhouse gases emissions from paddy rice soils. The pots were arranged in a two-factor factorial experiment with completely randomized design with three replications. Water management (continuous flooding (CF) and alternate wetting and drying (AWD) was arranged as factor A. Different rates of organic manure were assigned as factor B. In this study, cow dung manure was applied as an organic source based on the reduced global warming potential (GWP) value in previous study and widely used in the study area. The cow dung manure treatments (OM_0_ = no cow dung, OM_1_ = half of the recommended cow dung (2.5 t ha^-1^), OM_2_ = the recommended rate of cow dung (5 t ha^-1^) and OM_3_ = one and a half times the recommended rate of cow dung (7.5 t ha^-1^), were applied seven days before transplanting. The recommended rate of cow dung manure is 5 t ha^-1^. Each pot received the recommended fertilizer at the rates of 90 kg N ha^-1^, 30 kg P_2_O_5_ ha^-1^, and 20 kg K_2_O ha^-1^. Urea, T-super and potash were used as nutrient sources. Urea was applied as three equal split applications at the active tillering, panicle initiation and heading growth stages. T-super was applied only as a basal fertilizer at one day before transplanting, and potash fertilizer was applied in two equal split applications as a basal fertilizer and at panicle initiation. The soil was collected from a lowland irrigated rice field and analysed for pH (1:5 soil: water suspension), available N (Alkaline permanganate method), available P (9C-Olsen’s P-Malachite green), available K (1N Ammonium acetate extraction), total N% (Kjaldehl distillation method), organic matter% (Tyurin’s method), cation exchange capacity (CEC) (Leaching method) and texture (Pipette method). The cow dung manure was collected from farmer traditional heap method stored for ten months, and analysed for total N% (Kjaldehl distillation method) and organic carbon % (Tyurin’s method). Table 2 shows the physiochemical properties of the soil and cow dung.

**Table 2:** Physiochemical properties of experimental soil and cow dung manure used in the second pot experiment.

Analytical Item	Unit	Analytical Result	
Soil pH		7.4	Moderately alkaline
Available N	mg kg^-1^	50	Low
Available P	mg kg^-1^	13	Medium
Available K	mg kg^-1^	78	Low
Total N	%	0.17	
Organic matter	%	1.8	Low
CEC	cmol_c_ kg^-1^	11	Low
Sand	%	87	
Silt	%	4	
Clay	%	9	
Textural class			Loamy sand
**Cow dung manure**		**Dry season**	**Wet season**
Total N	%	1.32	1.2
Organic carbon	%	16	23.3

**Fig 2:**
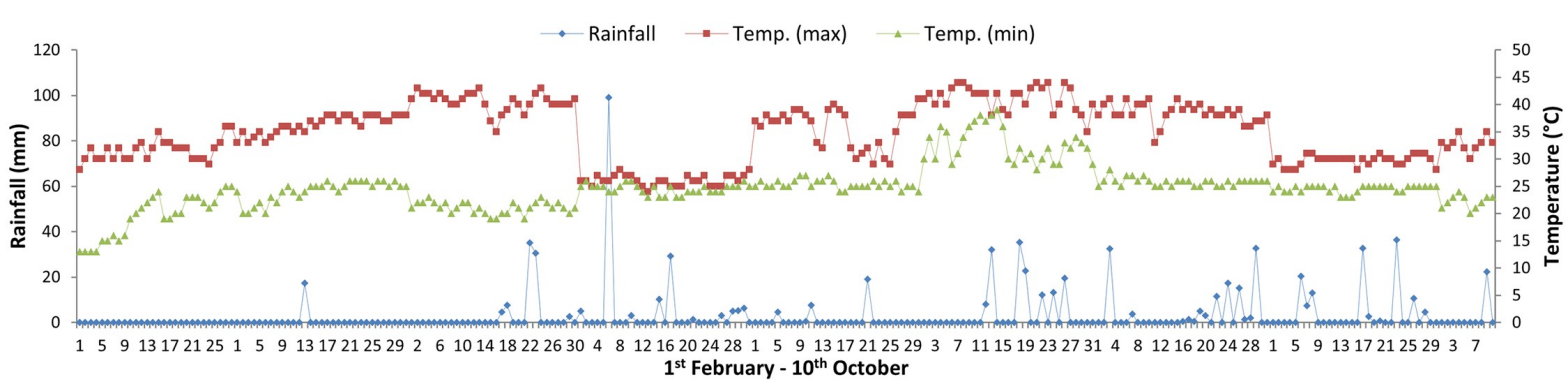
Daily rainfall, maximum and minimum temperatures in Madaya township, Myanmar during dry and wet seasons, 2017.

IR-50 rice variety (115 days) was used based on reduced emission in previous study and widely grown in the study area. Dry-season rice was transplanted on 1^st^ February 2017 and harvested on 14 May 2017. Wet-season rice was transplanted on 8 July 2017 and harvested on 11^st^ October 2017. Just after transplanting, a base (40 cm in diameter with 2.5 cm water seal, 5 cm in height) was placed around the plants used for gas sampling to avoid disturbing the environmental conditions around the rice plants during chamber deployment in both experiments. The base was equipped with a water seal to ensure a gas-tight closure. The base remained embedded in the soil throughout the rice growing period. Water tubes (PVC pipe-25 cm height with six row holes each 2.5 cm apart) were installed in the AWD pots at a depth of 15 cm below the soil surface between the seedlings and the base just after transplanting. For AWD pots, whenever there was no water in the water tube, irrigation water was applied to a 5 cm depth above the soil surface. The irrigation interval ranged from 4 to 9 days, and the amount ranged from 7 to 13 litres depending on the different rates of cow dung manure in the AWD pots. Withdrawal of water was started one week before the harvest period in all irrigated pots. During the rice growing seasons, weather data were collected from the Department of Agricultural Research, Madaya and are shown in Fig 2. The average minimum and maximum temperatures were 21.8°C and 35.6°C during the dry season and 26.8°C and 35.7°C during the wet season, respectively. The total rainfall amounts were 201.7 mm during the dry season and 420.6 mm during the wet season.

### Gas sample collection, analysis and calculation

A two-bonded chamber with a total capacity of 77 L (93 cm height) was used for collecting the gas sample. To thoroughly mix the gases in the chamber, the chamber was equipped with a small 12 volt DC fan connected with three 9-volt dry battery [43]. For CH_4_ calculation, the temperature was recorded with a digital thermometer (TT-508 Tanita, Tokyo, Japan). To compensate for the air pressure changes between the increased temperature and gas sampling, an air buffer bag (1-L Tedlar bag) was attached to the chamber. The silicon rubber tube connected with three-way stop cock was inserted airtight into a hole on the chamber. The gas sample was taken with an airtight 50 ml syringe by connecting it to the three-way stop cock and then transferred to a 20 ml pre-evacuated glass vial.

Gas sampling was performed at 7-day intervals starting from 1 day after transplanting until harvest by the closed chamber method in pot experiment 1. In pot experiment 2, the first two gas samplings were performed at one-week intervals after transplanting, and later gas samplings were performed at 10-day intervals. The gas samples were collected from 9:00 am until 12:00 am and three times (0, 15, 30 min) for each treatment for gas flux calculation.

Methane and N_2_O concentrations were analysed with a gas chromatograph (GC 2014, Shimadzu Corporation, Kyoto, Japan) equipped with a flame ionization detector (FID) and an electron capture detector (ECD). The amount of CH_4_ and N_2_O fluxes was calculated by using the following equation:

Q=(V/A)x(Δc/Δt)x(M/22.4)x(273/K)

where    Q = the flux of gas (mg m^-2^ min^-1^)

        V = the volume of the chamber (m^3^)

        A = the base area of the chamber (m^2^)

        (Δc/Δt) = the rate of increase or decrease in the gas concentration (mg m^-3^) per unit time

(min)

        M = the molar weight of the gas

        K = Kelvin temperature of the air temperature inside the chamber

Total emissions were calculated by interpolation method of sample gas analysis at each gas measurement for the growing period. In this study, the IPCC factors were used to calculate the combined GWPs for 100 years (GWP = (25×CH_4_) + (298×N_2_O)) in kg CO_2_-equivalents ha^-1^ [9] for CH_4_ and N_2_O. The yield was recorded from each pot. The grains were threshed, cleaned and sun-dried. Yields were adjusted at 14% moisture by using the following formula to remove the error due to the different moisture content, and grain moistures were measured by using a grain moisture metre (model: GMK-303RS).

Adjusted grain weight at 14% moisture level = A x W

where    A = Adjustment coefficient

        W = Weight of harvested grains

$$A=\frac{100‐\mathrm{moisture}\%}{86}$$

In both water management practices, the water was applied with 1.2 L water cup. The total amount of water applied throughout the growing season was recorded and water saving in AWD was calculated.

The greenhouse gas intensity (GHGI) was calculated by dividing the GWP by the rice grain yield [44–46].


$$GHGI=GWP/grain\;yield\;(kg\;CO_{2}‐eq.\;kg^{‐1}\;grain)$$

### Statistical analysis

The data were analysed by using Statistix (version 8.0). Mean comparisons were performed by least significant difference (LSD) test at the 5% level.

## Results

### First pot experiment during the wet season, 2016

#### Methane emission

**Fig 3:**
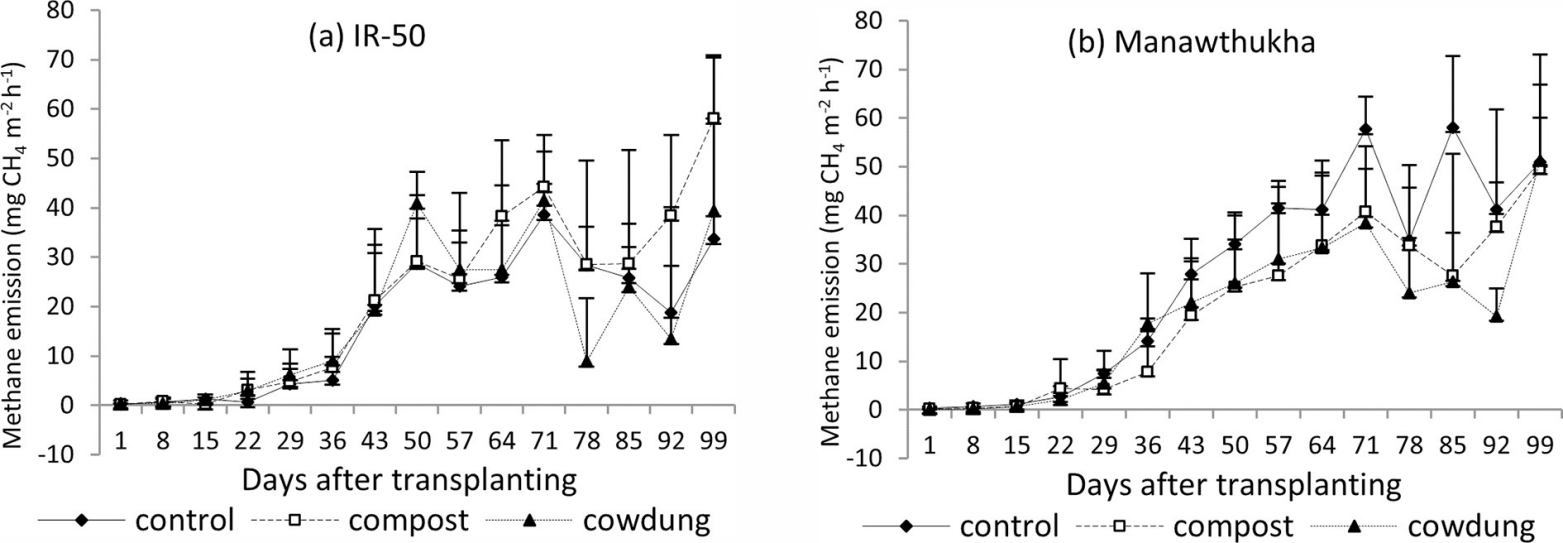
Methane emission of rice varieties at Yezin Agricultural University during the wet season, 2016. Mean value±standard deviation (n = 3).

During the early growth stage, low CH_4_ emission was observed for both varieties until 36 days after transplanting (DAT), and then CH_4_ emission flux gradually increased with some fluctuations until harvest (Fig 3). The mean cumulative CH_4_ emissions from the control, compost, and cow dung treatments were 0.893, 0.951 and 0.808 g CH_4_ kg^-1^ soil, respectively. Although the effect was not significant, cow dung amendment reduced cumulative CH_4_ emissions by 9.5% compared with the control and 15% compared to compost. When comparing the two rice varieties, the total cumulative CH_4_ emissions were significantly higher in the Manawthukha variety (1.084 g CH_4_ kg^-1^ soil) than in the IR-50 variety (0.683 g CH_4_ kg^-1^ soil) (P<0.0046) (Table 3). The IR-50 variety reduced cumulative CH_4_ emissions by 37% compared to the Manawthukha variety. During the rice growing season, cumulative CH_4_ emissions were higher in later growth stages (reproductive and ripening) than in the vegetative growth stage.

**Table 3:** Effects of manure and rice variety on greenhouse gases emission and grain yield of rice during wet season, 2016.

Treatment	CH_4_ (g kg^-1^ soil)	N_2_O (mg kg^-1^ soil)	Grain yield (g plant^-1^)	GHGI (kg CO_2_-eqv kg^-1^ grain)
Manure				
Control	0.893	3.218	122.3± 2.4 a	2.8
Compost	0.951	2.491	102.6±13.6 ab	3.8
Cow dung	0.808	2.135	79.6±15.6 b	4.3
LSD _0.05_	0.307	3.490	38.2	1.7
Variety				
Manawthukha	1.084 a	2.585	121.5±15.0 a	3.8
IR-50	0.683 b	2.644	81.6±8.0 b	3.5
LSD _0.05_	0.251	2.849	31.2	1.4
Pr>F				
Manure	0.6091	0.7922	0.0901	0.2332
Variety	0.0046	0.9649	0.0164	0.6719
Manure*Variety	0.6724	0.5766	0.6633	0.9931
CV (%)	27.67	106.09	29.92	38.66

Within each column, values with different alphabets indicate significant differences among the treatments at 5% of LSD test.

#### Nitrous oxide emission

**Fig 4:**
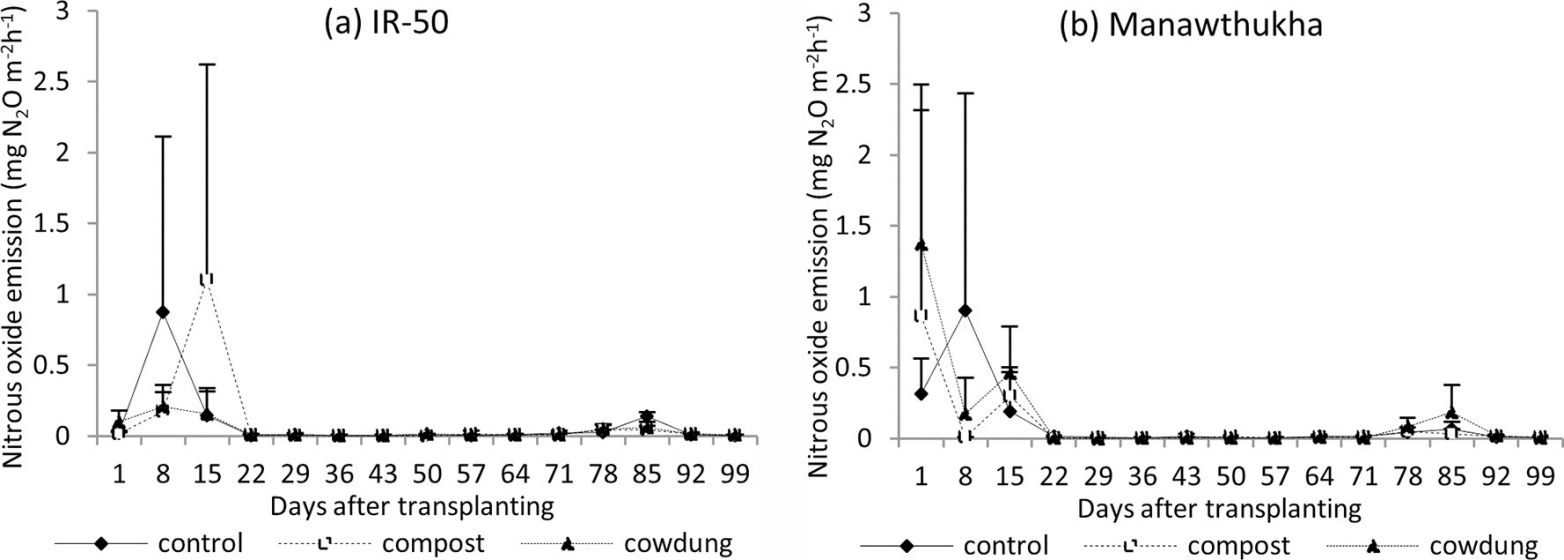
Nitrous oxide emission of rice varieties at Yezin Agricultural University during the wet season, 2016. Mean value±standard deviation (n = 3).

High nitrous oxide emission was observed during very early growth until 15 DAT in both varieties. After that, a small amount of N_2_O emission was found until harvest (Fig 4). Although there was no significant difference in N_2_O emissions among the manure treatments, higher emission (3.218 mg N_2_O kg^-1^ soil) was recorded from the control (no manure) compared to the compost (2.491 mg N_2_O kg^-1^ soil) and cow dung (2.135 mg N_2_O kg^-1^ soil) (Table 1). Cow dung manure reduced cumulative N_2_O emissions by 33.7% compared with the control and 14.3% compared to compost. There was also no significant difference in cumulative N_2_O emissions among the tested varieties: Manawthukha variety (2.585 mg N_2_O kg^-1^ soil), IR-50 (2.644 mg N_2_O kg^-1^ soil) (Table 3).

#### Global warming potential (GWP)

**Fig 5:**
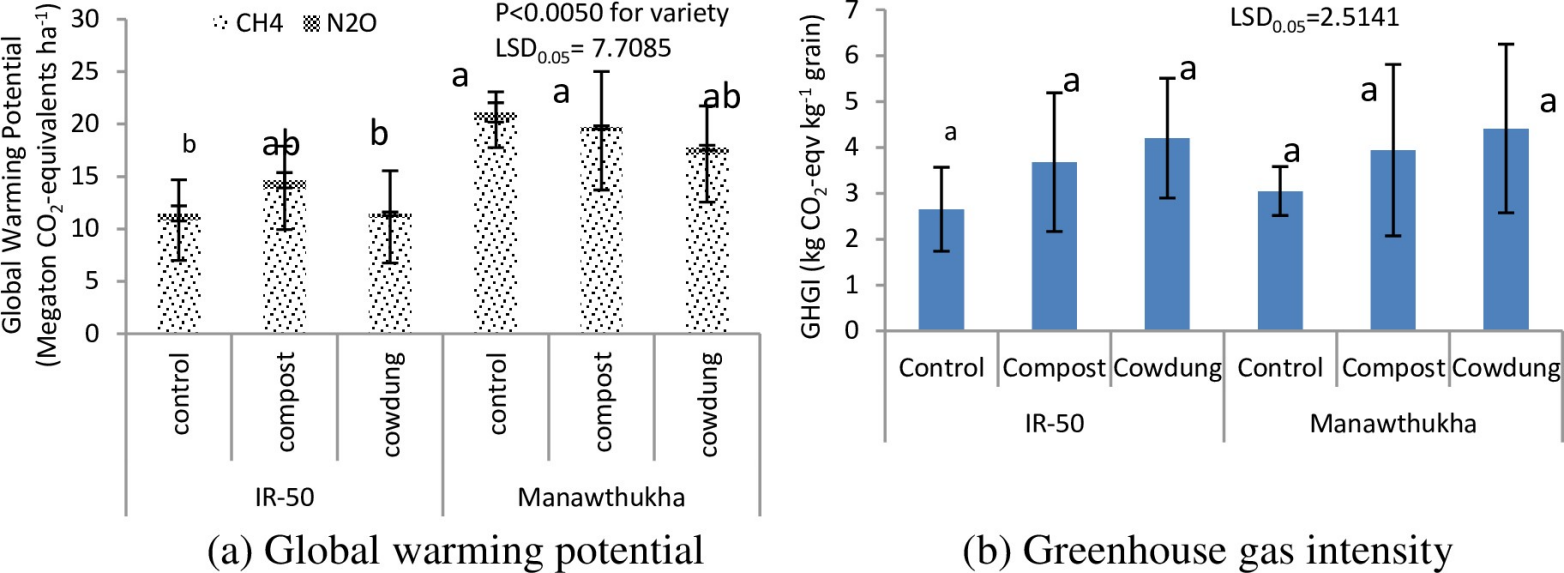
Effect of organic manure and rice varieties on (a) global warming potential and (b) greenhouse gas intensity during wet season, 2016. Mean value±standard deviation (n = 3).

GWP was not significantly different among manure treatments, although higher GWP (17.2 Mt CO_2_-eq. ha^-1^) was observed in the compost treatment followed by the control (16.3 Mt CO_2_-eq. ha^-1^) and cow dung treatment (14.6 Mt CO_2_-eq. ha^-1^) (Fig 5a). There was a significant difference in GWP among the varieties (P<0.0050). A higher GWP was observed for Manawthukha (19.5 Mt CO_2_-eq.ha^-1^) than for IR-50 (12.5 Mt CO_2_-eq. ha^-1^).

#### Rice yield

Grain yield was not significantly affected by the manure treatments. However, the numerically highest grain yield (122.3±2.4 g plant^-1^) was recorded from the control (no manure), followed by the compost (102.6±13.6 g plant^-1^) and cow dung treatments (79.6±15.6 g plant^-1^). There was significant different in grain yield between the varieties Manawthukha (121.5±15.0 g plant^-1^) and IR-50 (81.6±8.0 g plant^-1^) (P<0.0164) (Table 3).

#### Greenhouse gas intensity

Greenhouse gas intensity was not affected by manure management and varieties (Table 3). However, across manure management treatments, higher GHGI values were found in Manawthukha (3.8 kg CO_2_-eq. kg^-1^ grain) than in IR-50 (3.5 kg CO_2_-eq. kg^-1^ grain) (Fig 5B). There was no interaction between manure and rice varieties on the GHGI.

### Second pot experiment during the dry and wet seasons, 2017

#### Methane emission

**Fig 6:**
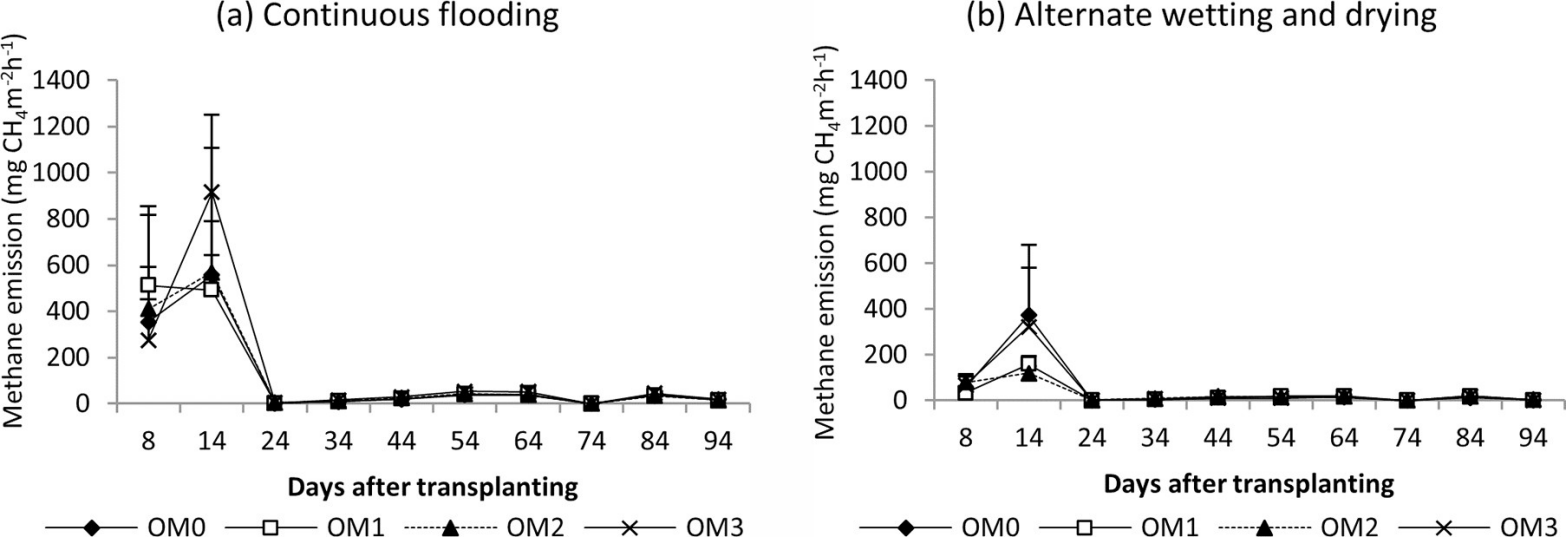
Methane emission from rice under (a) continuous flooding and (b) alternate wetting and drying during the dry season, 2017. Mean value±standard deviation (n = 3).

The seasonal methane emissions of rice are shown in Figs 6 (dry season) and 7 (wet season). In the dry season, high methane emissions were observed in the early growth stage and then decreased until harvest under both water regimes. In the wet season, a slight increase in emissions was recorded in the early growth stage, emissions peaked in the middle stage, and gradually decreased until harvest. There were significant differences in cumulative CH_4_ emissions among water management practices (P<0.0003) (Table 4). Higher cumulative CH_4_ emissions were observed under CF than AWD. Despite no significant difference among the cow dung manure rates, a generally higher amount of cow dung manure produced more methane emissions than lower rates (Fig 12A).

**Fig 7:**
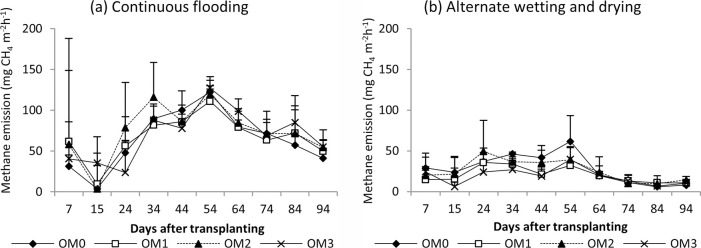
Methane emission from rice under (a) continuous flooding and (b) alternate wetting and drying during the wet season, 2017. Mean value±standard deviation (n = 3).

**Table 4:** Mean effects of water and manure management on greenhouse gases emission of rice during dry and wet seasons, 2017.

Treatment	Cumulative methane emission (g CH_4_ kg^-1^ soil)		Cumulative nitrous oxide emission (mg N_2_O kg^-1^ soil)	
	Dry season	Wet season	Dry season	Wet season
Water				
CF	3.327 a	2.009 a	0.590 b	0.183
AWD	0.996 b	0.682 b	1.392 a	0.344
LSD _0.05_	1.089	0.369	0.631	0.176
Manure				
OM_0_ (0 t ha^-1^)	2.129	1.361	0.786	0.306
OM_1_ (2.5 t ha^-1^)	2.036	1.279	1.621	0.201
OM_2_ (5 t ha^-1^)	2.007	1.460	0.817	0.234
OM_3_ (7.5 t ha^-1^)	2.475	1.283	0.740	0.312
LSD _0.05_	1.540	0.522	0.892	0.248
Pr>F				
Water	0.0003	0.0000	0.0159	0.0709
Manure	0.9114	0.8682	0.1546	0.7335
Water x Manure	0.9038	0.8217	0.0890	0.8582
CV (%)	58.21	31.74	73.53	77.08

Within each column, values with different alphabets indicate significant differences among the treatments at 5% of LSD test.

#### Nitrous oxide emission

**Fig 8:**
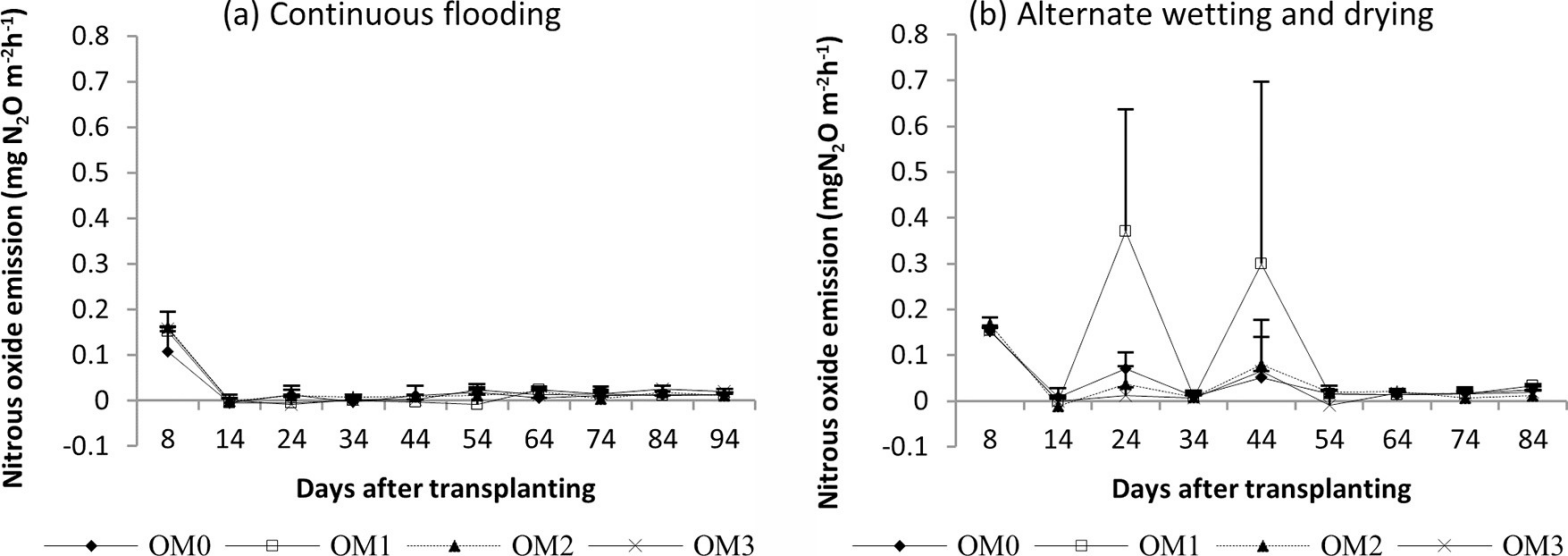
Nitrous oxide emission from rice under (a) continuous flooding and (b) alternate wetting and drying during the dry season, 2017. Mean value±standard deviation (n = 3).

The seasonal nitrous oxide emissions of rice are shown in Figs 8 (dry season) and 9 (wet season). Relatively high nitrous oxide emissions were found in the early growth stage, and reduced emissions were found in the later growth stage in both seasons. There were significant different in nitrous oxide emissions among the water management practices in dry season (P<0.0159) but not significant in wet season (Table 4). AWD gave a relative high N_2_O emission in both seasons. No significant difference was observed in N_2_O emission among the manure rates in either season.

**Fig 9:**
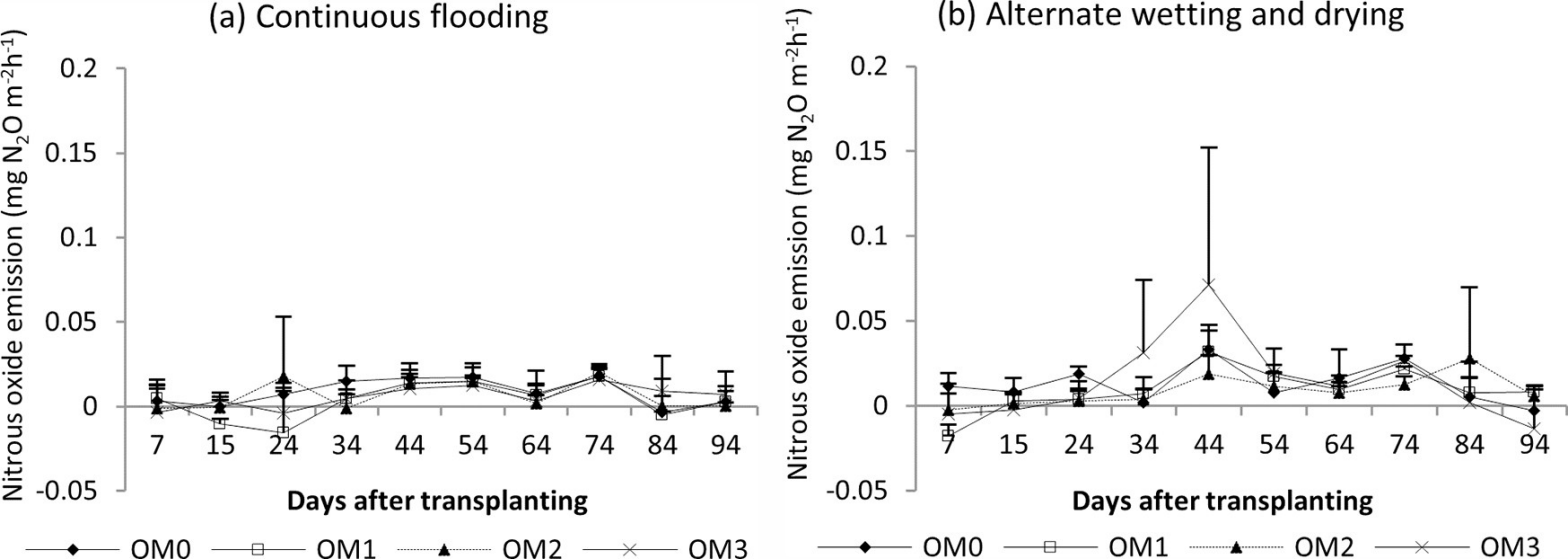
Nitrous oxide emission of rice under (a) continuous flooding and (b) alternate wetting and drying during the wet season, 2017. Mean value±standard deviation (n = 3).

#### Global warming potential (GWP)

**Fig 10:**
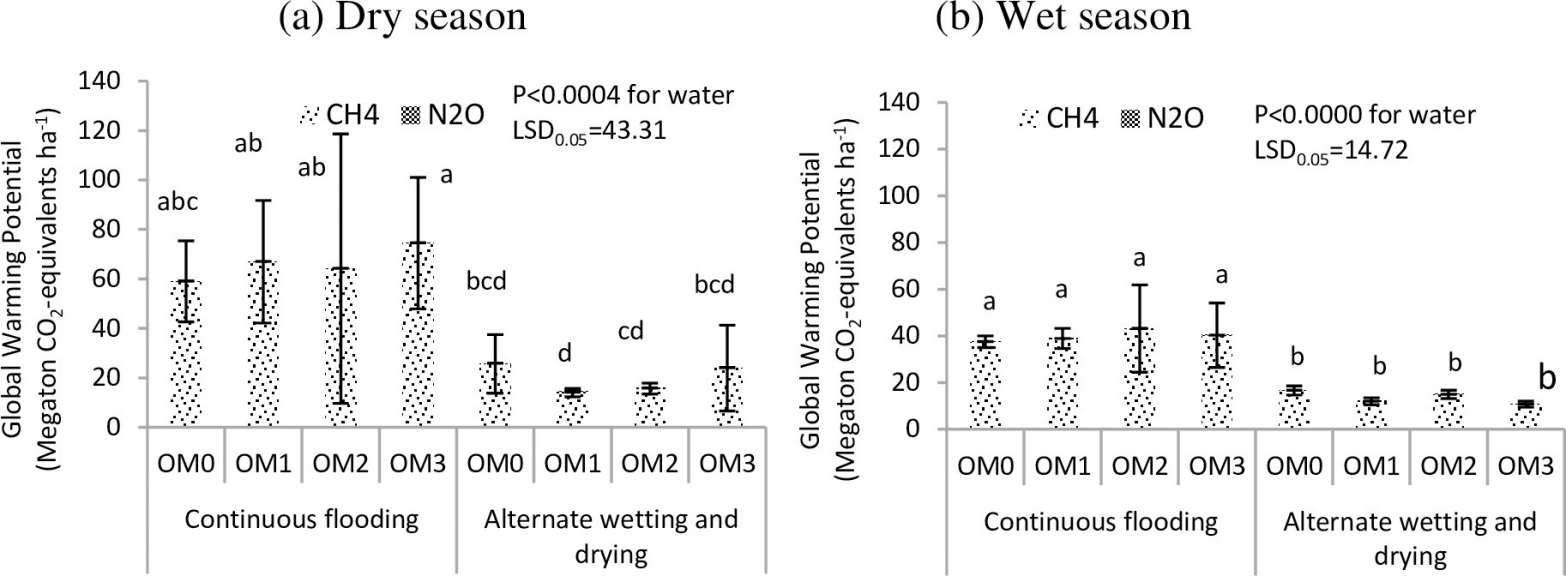
Effect of water and cow dung manure management on global warming potential of potted rice during dry (a) and wet (b) seasons, 2017. OM_0_-no cow dung, OM_1_- cow dung 2.5 t ha^-1^, OM_2_- cow dung 5.0 t ha^-1^, OM_3_- cow dung 7.5 t ha^-1^. Mean value±standard deviation (n = 3).

The GWP was significantly different among water management practices in both seasons (P<0.0004, P<0.0000). A higher GWPs (66.3 Mt CO_2_-eq. ha^-1^ and 40.0 Mt CO_2_-eq. ha^-1^) were observed under CF than under AWD (20.1 Mt CO_2_-eq. ha^-1^ and 13.7 Mt CO_2_-eq. ha^-1^) in the dry (Fig 10A) and wet seasons (Fig 10B), respectively. Generally, the large application of cow dung manure resulted in a higher GWP in both seasons (Fig 12B). The different rates of cow dung manure had no significant effect on the GWP in either season.

#### Rice yield

In this pot experiment, the grain yield was not significantly affected by water management and manure amendment in either rice season (Table 5). However, higher grain yields per plant were recorded with AWD than CF in both seasons. The manure rates had no significant effect on rice yield.

**Table 5:** Mean effects of water and manure management on rice yield during dry and wet seasons, 2017.

Treatment	Yield (g plant^-1^)		GHGI (kg CO_2_-eq. kg^-1^ grain)	
	Dry season	Wet season	Dry season	Wet season
Water				
CF	175.06±6.3	180.88±4.9	7.4 a	4.3 a
AWD	177.73±6.8	183.47±6.7	2.1 b	1.4 b
LSD _0.05_	12.67	14.27	2.3	0.7
Manure				
OM_0_ (0 t ha^-1^)	181.54±9.4	183.70±3.1	4.5	2.9
OM_1_ (2.5 t ha^-1^)	171.79±3.2	178.81±0.1	4.7	2.7
OM_2_ (5 t ha^-1^)	178.08±11.0	179.20±0.1	4.3	3.1
OM_3_ (7.5 t ha^-1^)	174.16±5.0	187.01±7.6	5.6	2.7
LSD _0.05_	17.92	20.18	3.2	1.1
Pr>F				
Water	0.6608	0.7060	0.0002	0.0000
Manure	0.6758	0.7984	0.8359	0.8283
Water x Manure	0.3976	0.6511	0.7742	0.8092
CV (%)	8.30	9.05	55.13	31.13

Within each column, values with different alphabets indicate significant differences among the treatments at 5% of LSD test.

#### Greenhouse gas intensity

**Fig 11:**
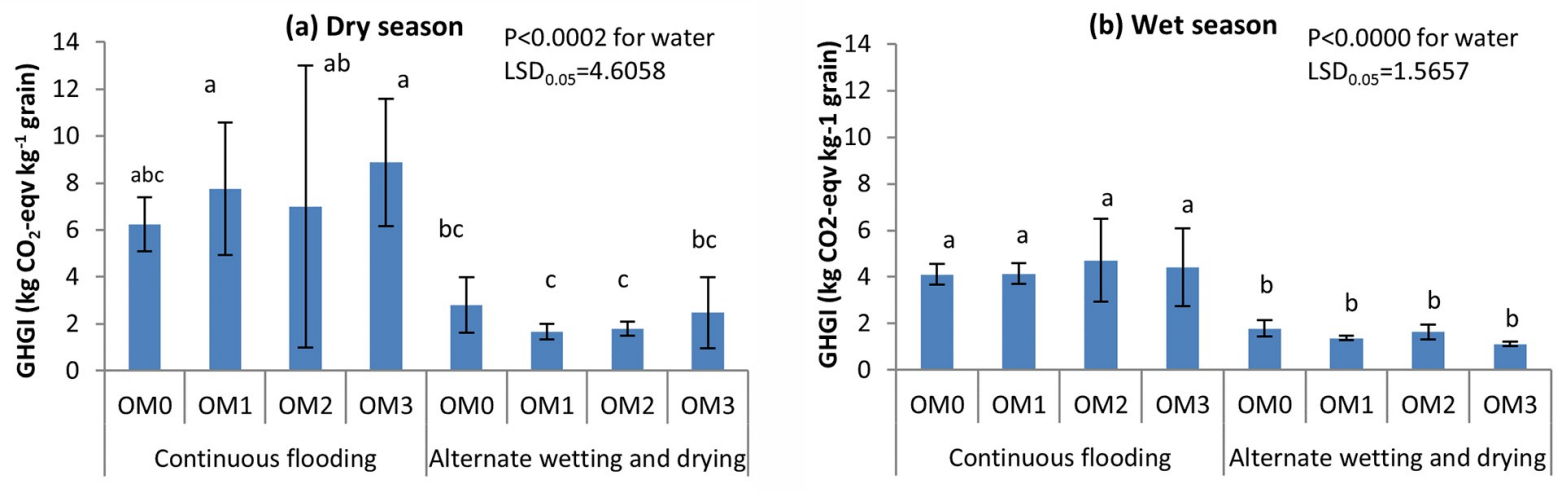
Effect of water and cow dung manure management on greenhouse gas intensity of potted rice during dry (a) and wet (b) seasons, 2017. OM_0_-no cow dung, OM_1_- cow dung 2.5 t ha^-1^, OM_2_- cow dung 5.0 t ha^-1^, OM_3_- cow dung 7.5 t ha^-1^. Mean value±standard deviation (n = 3).

Greenhouse gas intensities were significantly different among the water management practices in both seasons (P<0.0002, P<0.0000) (Table 5). Significantly higher GHGI values were found under CF than under AWD in both seasons (Fig 11). No significant differences were found in GHGI values among the manure management practices. However, the higher amount of cow dung manure affected the GHGI values under CF irrigation, but the effect of manure was suppressed by AWD.

#### Water input and water saving

Water inputs of rice as affected by water and manure management are shown in Table 6. There was significantly different of water input among the water management practices in either rice season (P<0.0001, P<0.0000). CF was irrigated more than AWD. The organic manure increases the water holding capacity of the soil. Accordingly, the higher amount of cow dung manure used less water. Water saving of treatments is shown in Table 7. AWD saved water 13.6% over CF in no cow dung manure in dry season and 19.1% in wet season. Across the manure management, the water saving of rice mainly depends on water management practices.

**Table 6:** Mean effects of water and manure management on water input of rice during dry and wet seasons, 2017.

Treatment	Water input _(I+R)_ (mm)	
	Dry season	Wet season
Water		
CF	1075.5 a	842.1 a
AWD	969.8 b	681.4 b
LSD _0.05_	42.4	32.7
Manure		
OM_0_	1030.5	775.0
OM_1_	1017.6	750.5
OM_2_	1015.4	740.6
OM_3_	1027.1	780.9
LSD _0.05_	60.0	46.2
Pr>F		
Water	0.0001	0.0000
Manure	0.9385	0.2372
Water x Manure	0.0955	0.7619
CV (%)	4.80	4.96

Within each column, values with different alphabets indicate significant differences among the treatments at 5% of LSD test.

**Table 7:** Comparison of water saving of rice as affected by water and cow dung manure management during dry and wet seasons, 2017.

Treatment		Water saving (%)	
Water	Cow dung manure	Dry season	Wet season
CF	OM_0_		
CF	OM_1_		
CF	OM_2_		
CF	OM_3_		
AWD	OM_0_	13.6	19.1
AWD	OM_1_	7.5	18.7
AWD	OM_2_	2.8	17.1
AWD	OM_3_	14.9	21.2

## Discussion

### Effect of manure and rice varieties on greenhouse gas emissions, global warming potential and rice yield

Methane production and emissions from flooded paddies are highly affected by the addition of organic matter [47]. The higher CH_4_ emission in this study was found in later growth stages (Fig 3) because the CH_4_ emission was associated with higher soil organic matter with increased microbial activities, decomposition of plant residues from fallen leaves and decayed roots, and higher availability of root exudates in the rhizosphere [48]. Although there was no significant difference based on manure management, cow dung manure reduced methane emissions. This study also agrees with that of Oo et al. [30], who reported the lower CH_4_ emissions from pots treated with well-decomposed cattle manure due to fewer carbon substrates with a reduction of potential CH_4_ precursors resulting from the preceding decompostion. In contrast to CH_4_ emissions, high N_2_O emissions were recorded in the early growth stage, and small amounts of emissions were recorded in the later growth stage (Fig 4). This could be due to the rapid nitrification with the presence of oxygen and denitrification with the utilization of NO_3_^-^ as electron acceptor in the initial stage with high temperature and low rainfall (Fig 1), and indigenous soil nitrogen (Table 1). After emission peaked initially, the rates of N_2_O emission were generally low due to continuous flooding. Another study reported that the consistently low soil redox potential under continuous flooding resulted in more complete denitrification and thus reduced N_2_O emissions [49]. The control (no manure) treatment resulted in higher N_2_O emissions than the compost and cow dung treatments. Lower nitrous oxide emission was resulted by incorporation of organic inputs due to nitrogen immobilization [50–52]. This study agrees with that of Shan and Yan [53], who reported that N_2_O emissions were significantly reduced by crop residue return combined with synthetic N fertilizers compared with emissions from treatments only received synthetic N fertilizer.

The selection of suitable rice varieties might play a significant role in regulating CH_4_ emissions from rice fields [54]. The results of this study showed that there was a significant difference between the tested rice varieties (Fig 3 and Table 3). The result was in agreement with other findings, which highlighted that there were substantial differences in the rates of CH_4_ emission among different rice varieties [30, 55, 56]. As ninety percent of methane emissions to the atmosphere are through rice plants [57], the Manawthukha variety, which has a longer growth duration, emitted more CH_4_ than IR-50, which has a shorter growth duration. Previous studies [42, 58–60] have also reported that the CH_4_ flux from late maturing rice is higher than that from early maturing rice. According to this research finding, a shorter growth duration (IR-50) variety can be used to reduce methane emissions. When compared to the two rice varieties, IR-50 resulted in higher N_2_O emissions than the Manawthukha variety. This could be due to the favourable effect of root exudates of IR-50 on the nitrification process in the soil. Gogoi and Baruah [25] reported that the main driving forces influencing N_2_O emission in rice were soil NO_3_-N, soil organic carbon. There was no interaction between manure and rice varieties. The compost and Manawthukha variety emitted higher CH_4_ due to longer decomposing time and support of substrates for methanogens. The control treatment and IR-50 variety resulted in higher N_2_O emission due to nitrification and denitrification of inorganic nutrient in the favour of root exudates (Fig 5A).

Cow dung manure resulted in a 10.4% reduction in GWP compared to the control (no manure) with reduced CH_4_ and N_2_O emission. Combination of decomposed cow dung manure + mineral fertilizer might suppress the available carbon and nitrogen for CH_4_ and N_2_O production. Unfortunately, in our study, we couldn’t measure the carbon and nitrogen at every gas sampling and their mechanisms. While an increased GWP (5.5%) was found in the compost compared to the control. Huang et al. [61] reported that incorporation of organic residues provides a source of readily available C and N in the soil and subsequently influences N_2_O emissions. Manawthukha had a higher GWP (56%) than IR-50. These results were supported by Zheng et al. [62], who reported that yield-scaled GWP at 80–90 days of growth duration after transplanting was 87% higher than that at 70–80 days of growth duration after transplanting. Feng et al. [63] also reported that yield-scaled GWP in late rice in a double-rice cropping system was 73% higher than that for varieties with 90–100 days of growth duration after transplanting.

A high grain yield of rice was recorded from the control due to the mineralization of indigenous soil organic matter and inorganic fertilizer (Table 3). Oo et al. [64] reported a high grain yield under inorganic sources of nutrients due to the immediate release and availability of nutrients. Relative to IR-50, Manawthukha had a higher grain yield by 48.9% whereas the GWP was reduced in IR-50 than in Manawthukha. Therefore, considering the mitigation practice for GWP from rice production, IR-50 and cow dung manure was experimented in 2017 rice seasons.

### Effect of water management and manure application on greenhouse gas emissions, global warming potential and rice yield

In the dry season, high methane emissions were observed in the early growth stage and then decreased until harvest in both water regimes (Fig 6). The early increase in CH_4_ emission was due to the indigenous soil carbon content and availability of substrates, and the decrease in the later growth stage was due to the senescence of older leaves and non-availability of substrate as the crop approached maturity [65–67]. In the wet season, a slight increase in emission was recorded in the early growth stage, and emission was highest in the middle stage and gradually decreased until harvest (Fig 7). A higher rate of CH_4_ production is attributed to the availability of organic substrates from the previous crop residues in the form of plant-derived C through processes such as root exudation and release of fallen leaves and intensively reduced conditions in the rice rhizosphere [54, 68]. The gradual decrease in CH_4_ emission in later growth stage was due to the decomposition and non-availability of substrates depending on temperature (Fig 2). Higher cumulative CH_4_ emissions were observed under CF than under AWD in both seasons because the anoxic conditions increased the methanogen population and favoured methane production. The methane emission was higher in the dry season than in the wet season because it depended on the availability of indigenous soil carbon and decomposition process favoured by high temperature in the dry season, and depletion of soil carbon and previous crop resides decomposition hindered by low temperature in wet season (Fig 2). Relatively high nitrous oxide emissions were observed in the early growth stage, and reduced emissions were observed in the later growth stage in both seasons (Figs 8 and 9). This was due to the indigenous soil nitrogen content in the early stage, and depletion of nutrients and the available nitrogen content for nitrification and denitrification in the later stage. Higher N_2_O emissions were observed under AWD than CF in both seasons [54, 69, 70]. AWD increased N_2_O emissions by 135% compared to CF in dry season and 88% in wet season. Similar to the CH_4_ emission pattern, higher N_2_O emissions were observed in the dry season than in the wet season because of the soil nitrogen availability and the favoured soil condition (high temperature and low rainfall) for nitrification and denitrification in the dry season [71, 72].

**Fig 12:**
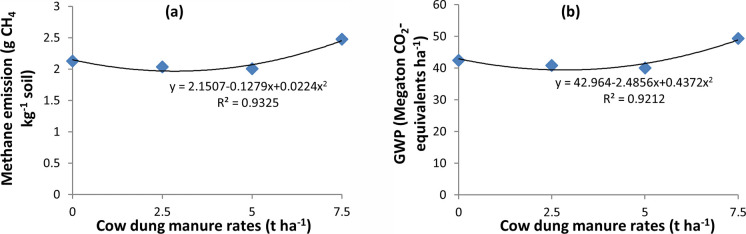
Relationship between methane emission and different cow dung manure rates (a) and between GWP and different cow dung manure rates (b) in dry season, 2017.

Generally higher rates of cow dung manure resulted in higher CH_4_ and N_2_O emission because it provided carbon and nitrogen sources for methanogenesis and nitrification and denitrification process. Methane is produced by methanogenic bacteria during the anaerobic digestion of organic substrates [4] and N_2_O production is observed by soil water content and availability of substrates (nitrate and easily degradable organic matter) for denitrification [73]. However, the polynomial distribution of methane emission with different cow dung manure rates (Fig 12A) and polynomial distribution of GWP with different cow dung manure rates (Fig 12B) were observed in dry season. The polynomial regression equation for methane emission is y = 2.1507–0.1279x+0.0224x^2^. The equation showed that the methane emission decreased in the rate of 0.1279 g CH_4_ kg^-1^ soil at every increased unit (ton) of cow dung manure applied, after that increased in the rate of 0.0224 g CH_4_ kg^-1^ soil at every increased unit (ton) of cow dung manure applied. The coefficient of determination (R^2^) showed that 93% of variation in methane emission could be accounted by the quadratic regression equation of different cow dung manure rates. The polynomial regression equation for GWP is y = 42.964–2.4856x+0.4372x^2^. The equation showed that the GWP decreased in the rate of 2.4856 Mt CO_2_-equivalent ha^-1^ at every increased unit (ton) of cow dung manure applied, after that increased in the rate of 0.4372 Mt CO_2_-equivalent ha^-1^ at every increased unit (ton) of cow dung manure applied. The coefficient of determination (R^2^) showed that 92% of variation in GWP could be accounted by the quadratic regression equation of different cow dung manure rates. According to these results, it would be recommended that 5 t ha^-1^ should be applied for improving soil fertility and reduced greenhouse gas emission. No interactive effect between water management and the application of different cow dung manure rates was found on CH_4_ emissions in either season. However, the effect of the application of different cow dung manure rates on N_2_O emissions was influenced by alternate wetting and drying irrigation practices in the dry season but not in the wet season. Therefore, the nitrification and denitrification processes of soil are influenced by the soil moisture content [74–77].

A higher GWP was observed under CF in accordance with the higher methane emissions in both seasons (Fig 10). Methane emission mainly contributes to the GWP from paddy production. Many studies [39, 54, 78–82] reported that N_2_O emissions contribute much less to the global warming potential than those of CH_4_. Therefore, the water regime in paddy production is the main factor controlling CH_4_ emissions from rice fields [39, 54, 83].

AWD resulted in a higher grain yield per plant than did CF in both seasons (Table 3) because it strengthened the air exchange between the soil and atmosphere and supplied sufficient oxygen to the root system to accelerate soil organic matter mineralization which increase soil fertility and favour rice growth [84, 85]. Yang and Zhang [86] reported an increase in paddy yield under AWD due to the increase in the proportion of productive tillers. In this study, AWD saved water about 10% over CF in dry season and 19% in wet season. Zhang et al. [87] also indicated water saving of 35% under AWD with a 10% yield increase relative to that under CF. Liu et al. [88], Ye et al. [89], and Djaman et al. [90] found that grain yield increased with reduced water input by AWD. Grain yield was not affected by the different rates of cow dung manure application because its decomposition was influenced by biotic and soil-environmental factors. Relative to the CF water management, AWD produced comparable grain yields, increased by 1.5% and decreased GHGI by 69% (Table 5). This suggests that by adopting alternate wetting and drying irrigation, it would be possible to achieve the dual goals of maintaining productivity while minimizing the global warming potential of rice cultivation.

## Conclusion

For sustainable agriculture, organic manures should be added at a recommended amount to improve the rice yield and reduce greenhouse gas emissions. From our findings, the application of cow dung manure can be recommended in paddy production since it mitigated the global warming potential compared to that of the control and compost groups, although it resulted in a lower yield potential. Additionally, the short duration rice variety had a lower GWP and a lower GHGI value while maintaining the potential rice yield. Manure-induced greenhouse gas emissions were suppressed by AWD irrigation practices, with significant GHGI values. Thus, short duration varieties are highly recommended with AWD irrigation and application of 5 t ha^-1^ cow dung manure to reduce greenhouse gas emissions and maintain rice yield under the soil-environmental conditions of Myanmar. In our study, there was no relationship between GWP and rice yield. Therefore, the choice of rice varieties should be combined with soil-environmental factors and cultivation systems to mitigate greenhouse gas emissions while increasing rice yields for sustainable rice production. Further studies under field condition are needed to measure the effect of manure and mineral fertilizer on greenhouse gas emission, global warming potential and rice yield under water management conditions for better understanding of emission mechanisms.
