# Correction: Influence of rice varieties, organic manure and water management on greenhouse gas emissions from paddy rice soils

**DOI:** 10.1371/journal.pone.0263554

**Published:** 2022-01-31

**Authors:** Ei Phyu Win, Kyaw Kyaw Win, Sonoko D. Bellingrath-Kimura, Aung Zaw Oo

The first author, Ei Phyu Win, is incorrectly noted as the corresponding author. The correct corresponding author is Sonoko D. Bellingrath-Kimura. Sonoko D. Bellingrath-Kimura’s email address is: sonoko.bellingrath-kimura@zalf.de.

There are errors in the Author Contributions. The correct contributions are:

**Conceptualization:** Ei Phyu Win

**Data curation:** Ei Phyu Win

**Formal analysis:** Ei Phyu Win

**Funding acquisition:** Ei Phyu Win

**Investigation:** Ei Phyu Win

**Methodology:** Ei Phyu Win

**Project administration:** Kyaw Kyaw Win

**Resources:** Ei Phyu Win

**Software:** Ei Phyu Win

**Supervision:** Sonoko D. Bellingrath-Kimura, Aung Zaw Oo

**Validation:** Sonoko D. Bellingrath-Kimura, Aung Zaw Oo

**Visualization:** Sonoko D. Bellingrath-Kimura, Aung Zaw Oo

**Writing–original draft:** Ei Phyu Win

**Writing–review & editing:** Sonoko D. Bellingrath-Kimura, Aung Zaw Oo
