# Supplementary material for: Influence of rice varieties, organic manure and water management on greenhouse gas emissions from paddy rice soils
Source: PLoS One. 2021 Jun 30;16(6):e0253755. doi: 10.1371/journal.pone.0253755 (PMC8244889; doi:10.1371/journal.pone.0253755)
Supplement: Attachment — Submitted filename: Reviewer _Lucy.pdf [file pone.0253755.s001.pdf]

This study is interesting, however the design has a number of deficiencies, for example one part considers wet and dry season, the other part only considers one season. All plot received recommended fertilizer, then manure/compost was added what was the purpose of adding manure/compost. Why were the nutrients from manure/nutrients ignored? Tables reported data for C, N etc then in the methods there is no mention of how these nutrients were analyzed. The discussion was poorly thought through and is inconsistent with series of statements that are not supported by data or literature. The discussion will need to be rewritten. I would recommend not publishing this paper in the status it is but have the author do major changes for consideration, which will include re-write abstract, discussion and include the methods missing. In addition to other suggestions below.

ABSTRACT

Poorly constructed abstract, inconsistent and not flowing, has no concluding remarks and the findings are not tied together well.

INTRODUCTION

The first part of the paper did not have line numbers, so it was difficult to reference here

Paragraph 7 (see pdf document) you have "Gathorne-Hardy 2013a", but I dont see "Gathorne-Hardy 2013b" was this just a copy and paste?

Paragraph 7 (see pdf document)Replace "due to lack" with " in absence of"

Paragraph 7 (see pdf document) What do you mean by off-site?

MATERIALS AND METHODS

There is so much missing in the methods

- Table 2.1: How was N,P, K and O.C determined? Which analysis methods?
- In soils did you just determine organic carbon? What about total carbon?
- How was alternate wetting and drying done?

Paragraph 1 Why did you consider wet season only, while the second study you covered both wet and dry seasons?

Paragraph 1 Did the application dependent on carbon content or nitrogen content? clarify

Paragraph 2 If each plot received recommended fertilizer, what was the purpose of adding manure?

RESULTS

Line replace not significantly different

DISCUSSION

Line 241 Did you consider how availability of different acceptors would affect CH4 production

Line 247 Do you have data to support that cow dung was humified organic matter than others (compost)? Or this is an assumption? Also the difference was not significant.

Line 253 This should be a complement to your observation of low CH4 in the initial stage because nitrification occuring could mean presence of oxygen and denitrification

could mean utilization of NO3 as electron acceptor, refer to the point I made above

Line 280 This is an assumption without a reference?

Line 282 You cant have a paragraph that is one statement

Line 285 This was just a repetition of the results without explaining why Manure reduced GWP and composed increased GWP

Line 293 As I indicated in the methods section, If each plot received recommended fertilizer, what was the purpose of adding manure? Why was the nutrient in

the manure not considered, does it mean manure just added extra nutriments that plant did not need for production?

Line 296 This is not consistent with your finding, in your finding it was inorganic fertilizer that recorded higher grain yield but not organic+inorganic fertilizer.

This is not consistent.

Line 308 Does the dry season affect the water level?

Line 311 Did you just copy and paste this citation, I see 2016b but there is no 2016a

Line 332 Does this contrast your statement in line 247

Line 340 Is this a suggestion? or did you test for it? or do you have a citation to support it?

Line 351 What do you mean by this? You throw in statements without clarity

CONCLUSION

Line 372 But this study indicated that grain yield was in the order of control> compost and cow dung? And GHGI was in the order of cowdung> compost > control

Table 2.1 and Table 2.2 do you have P values or SEM?

- Put letter on bar graphs to show if they are significantly different
