# Supplementary material for: Influence of rice varieties, organic manure and water management on greenhouse gas emissions from paddy rice soils
Source: PLoS One. 2021 Jun 30;16(6):e0253755. doi: 10.1371/journal.pone.0253755 (PMC8244889; doi:10.1371/journal.pone.0253755)
Supplement: Attachment — Submitted filename: PONE-D-20-31281_reviewer 1.pdf [file pone.0253755.s004.pdf]

PLOS ONE

Influence of rice varieties, organic manure and water management on greenhouse gas emissions from paddy rice soils

--Manuscript Draft--

**Manuscript Number:**	PONE-D-20-31281
**Article Type:**	Research Article
**Full Title:**	Influence of rice varieties, organic manure and water management on greenhouse gas emissions from paddy rice soils
**Short Title:**	Rice varieties, organic manure and water management on greenhouse gas emissions
**Corresponding Author:**	Ei Phyu Win, Ph.D.<br>Yezin Agricultural University<br>Nay Pyi Taw, Nay Pyi Taw MYANMAR
**Keywords:**	mitigation; greenhouse gas emissions; rice varieties; organic fertilizer; water management
**Abstract:**	To assess the effect of manure and water management practices and rice varieties on greenhouse gas emissions, two pot experiments were conducted. The first pot experiment was conducted at the Department of Agronomy, Yezin Agricultural University, Myanmar, in the wet season from June to October 2016. Three different organic manures, control (no manure), compost and cow dung, and two rice varieties, Manawthukha (135 days) and IR-50 (115 days), were tested. The results showed that cumulative CH_4 emission from Manawthukha (759.1 kg CH_4 ha^{-1}) was significantly higher than that from IR-50 (478.7 kg CH_4 ha^{-1}) in accordance with the yield because of the longer growth duration of the former. In contrast, significantly higher cumulative nitrous oxide emissions were found for IR-50 (1.85 kg N_2O ha^{-1}) than for Manawthukha (1.81 kg N_2O ha^{-1}). However, IR-50 showed less global warming potential (GWP) than Manawthukha. High CH_4 emissions (665.8 kg CH_4 ha^{-1}) were observed from the compost, and high nitrous oxide emissions (2.25 kg N_2O ha^{-1}) were observed from the control (no manure). Although not significant, the numerically lowest CH_4 and N_2O emissions were observed in the cow dung manure treatment (565.8 kg CH_4 ha^{-1}, 1.49 kg N_2O ha^{-1}) compared to those of the control and compost. To determine the effect of water management and organic manures on greenhouse gas emissions, second pot experiments were conducted in Madaya township during the dry and wet seasons from February to October 2017. Two water management practices (continuous flooding (CF) and alternate wetting and drying (AWD)) and four cow dung manure rates ((1) 0 (2) 2.5 t ha^{-1} (3) 5 t ha^{-1} (4) 7.5 t ha^{-1}) were tested. The different cow dung manure rates did not significantly affect grain yield or greenhouse gas emissions in this experiment. Across the manure treatments, AWD irrigation significantly reduced CH_4 emissions by 70% during the dry season and 66% during the wet season. Although a relative increase in N_2O emissions under AWD was observed in both rice seasons, the effect was not significant. As expected, the AWD irrigation significantly reduced the GWP due to reduced CH_4 emissions without affecting the rice yield.
**Order of Authors:**	Ei Phyu Win, Ph.D.
	Kyaw Kyaw Win
	Sonoko D. Bellingrath-Kimura
	Aung Zaw Oo
**Additional Information:**	
**Question**	**Response**
**Financial Disclosure**<br><br>Enter a financial disclosure statement that describes the sources of funding for the	Ei Phyu Win<br>one grant<br>Cuomo Foundation<br>https://www.cuomo.foundation/

work included in this submission. Review the submission guidelines for detailed requirements. View published research articles from *PLOS ONE* for specific examples.<br><br>This statement is required for submission and **will appear in the published article** if the submission is accepted. Please make sure it is accurate.<br><br>**Unfunded studies**<br>Enter: *The author(s) received no specific funding for this work.*<br><br>**Funded studies**<br>Enter a statement with the following details:<br>• Initials of the authors who received each award<br>• Grant numbers awarded to each author<br>• The full name of each funder<br>• URL of each funder website<br>• Did the sponsors or funders play any role in the study design, data collection and analysis, decision to publish, or preparation of the manuscript?<br>• **NO** - Include this sentence at the end of your statement: *The funders had no role in study design, data collection and analysis, decision to publish, or preparation of the manuscript.*<br>• **YES** - Specify the role(s) played.<br><br>* typeset	No
**Competing Interests**<br><br>Use the instructions below to enter a competing interest statement for this submission. On behalf of all authors, disclose any competing interests that could be perceived to bias this work—acknowledging all financial support and any other relevant financial or non-financial competing interests.<br><br>This statement **will appear in the published article** if the submission is accepted. Please make sure it is accurate. View published research articles from *PLOS ONE* for specific examples.	The authors have declared that no competing interests exit.

**NO authors have competing interests**<br><br>Enter: *The authors have declared that no competing interests exist.*<br><br>**Authors with competing interests**<br><br>Enter competing interest details beginning with this statement:<br><br>*I have read the journal's policy and the authors of this manuscript have the following competing interests: [insert competing interests here]*<br><br>* typeset	
**Ethics Statement**<br><br>Enter an ethics statement for this submission. This statement is required if the study involved:<br><br>• Human participants<br>• Human specimens or tissue<br>• Vertebrate animals or cephalopods<br>• Vertebrate embryos or tissues<br>• Field research<br><br>Write "N/A" if the submission does not require an ethics statement.<br><br>General guidance is provided below. Consult the submission guidelines for detailed instructions. **Make sure that all information entered here is included in the Methods section of the manuscript.**	N/A

**Format for specific study types**<br><br>**Human Subject Research (involving human participants and/or tissue)**<br>• Give the name of the institutional review board or ethics committee that approved the study<br>• Include the approval number and/or a statement indicating approval of this research<br>• Indicate the form of consent obtained (written/oral) or the reason that consent was not obtained (e.g. the data were analyzed anonymously)<br><br>**Animal Research (involving vertebrate animals, embryos or tissues)**<br>• Provide the name of the Institutional Animal Care and Use Committee (IACUC) or other relevant ethics board that reviewed the study protocol, and indicate whether they approved this research or granted a formal waiver of ethical approval<br>• Include an approval number if one was obtained<br>• If the study involved *non-human primates*, add *additional details* about animal welfare and steps taken to ameliorate suffering<br>• If anesthesia, euthanasia, or any kind of animal sacrifice is part of the study, include briefly which substances and/or methods were applied<br><br>**Field Research**<br><br>Include the following details if this study involves the collection of plant, animal, or other materials from a natural setting:<br>• Field permit number<br>• Name of the institution or relevant body that granted permission	
**Data Availability**<br><br>Authors are required to make all data underlying the findings described fully available, without restriction, and from the time of publication. PLOS allows rare exceptions to address legal and ethical concerns. See the PLOS Data Policy and FAQ for detailed information.	Yes - all data are fully available without restriction

A Data Availability Statement describing where the data can be found is required at submission. Your answers to this question constitute the Data Availability Statement and **will be published in the article**, if accepted.<br><br>**Important:** Stating 'data available on request from the author' is not sufficient. If your data are only available upon request, select 'No' for the first question and explain your exceptional situation in the text box.<br><br>Do the authors confirm that all data underlying the findings described in their manuscript are fully available without restriction?	
**Describe where the data may be found in full sentences. If you are copying our sample text, replace any instances of XXX with the appropriate details.**<br><br>• If the data are **held or will be held in a public repository**, include URLs, accession numbers or DOIs. If this information will only be available after acceptance, indicate this by ticking the box below. For example: *All XXX files are available from the XXX database (accession number(s) XXX, XXX.).*<br>• If the data are all contained **within the manuscript and/or Supporting Information files**, enter the following: *All relevant data are within the manuscript and its Supporting Information files.*<br>• If neither of these applies but you are able to provide **details of access elsewhere**, with or without limitations, please do so. For example:<br><br>*Data cannot be shared publicly because of [XXX]. Data are available from the XXX Institutional Data Access / Ethics Committee (contact via XXX) for researchers who meet the criteria for access to confidential data.*<br><br>*The data underlying the results presented in the study are available from (include the name of the third party*	All relevant data are withing the manuscript file.

and contact information or URL).<br>• This text is appropriate if the data are owned by a third party and authors do not have permission to share the data.<br><br>* typeset	
Additional data availability information:	Tick here if your circumstances are not covered by the questions above and you need the journal’s help to make your data available.

Powered by Editorial Manager® and ProduXion Manager® from Aries Systems Corporation

Manuscript

Click here to access/download;Manuscript;3102020-PLOS.docx

Influence of rice varieties, organic manure and water management on greenhouse gas emissions from paddy rice soils

Ei Phyu Win[1], Kyaw Kyaw Win[1], Sonoko D. Bellingrath-Kimura[2], Aung Zaw Oo[3]

[1]Department of Agronomy, Yezin Agricultural University, Myanmar
[2]Institute of Land Use Systems, Faculty of Life Science, Humboldt-Universität zu Berlin, Germany
[3]Japan International Research Center for Agricultural Sciences, 1-1 Ohwashi, Tsukuba, Ibaraki 3058686, Japan

Abstract

To assess the effect of manure and water management practices and rice varieties on greenhouse gas emissions, two pot experiments were conducted. The first pot experiment was conducted at the Department of Agronomy, Yezin Agricultural University, Myanmar, in the wet season from June to October 2016. Three different organic manures, control (no manure), compost and cow dung, and two rice varieties, Manawthukha (135 days) and IR-50 (115 days), were tested. The results showed that cumulative CH_4 emission from Manawthukha (759.1 kg CH_4 ha^{-1}) was significantly higher than that from IR-50 (478.7 kg CH_4 ha^{-1}) in accordance with the yield because of the longer growth duration of the former. In contrast, significantly higher cumulative nitrous oxide emissions were found for IR-50 (1.85 kg N_2O ha^{-1}) than for Manawthukha (1.81 kg N_2O ha^{-1}). However, IR-50 showed less global warming potential (GWP) than Manawthukha. High CH_4 emissions (665.8 kg CH_4 ha^{-1}) were observed from the compost, and high nitrous oxide emissions (2.25 kg N_2O ha^{-1}) were observed from the control (no manure). Although not significant, the numerically lowest CH_4 and N_2O emissions were observed in the cow dung manure treatment (565.8 kg CH_4 ha^{-1}, 1.49 kg N_2O ha^{-1}) compared to those of the control and compost. To determine the effect of water management and organic manures on greenhouse gas emissions, second pot experiments were conducted in Madaya township during the dry and wet seasons from February to October 2017. Two water management practices (continuous flooding (CF) and alternate wetting and drying (AWD)) and four cow dung manure rates ((1) 0 (2) 2.5 t ha^{-1} (3) 5 t ha^{-1} (4) 7.5 t ha^{-1}) were tested. The different cow dung manure rates did not significantly affect grain yield or greenhouse gas emissions in this experiment. Across the manure treatments, AWD irrigation significantly reduced CH_4 emissions by 70% during the dry season and 66% during the wet season. Although a relative increase in N_2O emissions under AWD was observed in both rice seasons, the effect was not significant. As expected, the AWD irrigation significantly reduced the GWP due to reduced CH_4 emissions without affecting the rice yield.

Keywords: mitigation, greenhouse gas emissions, rice varieties, organic fertilizer, water management

I. Introduction

Developing new strategies is necessary to achieve the dual goals of ensuring food security and protecting natural resources and the environment through reduced greenhouse gas (GHG) emissions (Burney et al. 2010: Tilman et al. 2011). It is estimated that N_2O and CH_4 emissions may increase by 35-60% and 60%, respectively, by 2030 (Smith et al. 2007). Flooded rice soils are an important source of global CH_4 emissions (Le Mer and Roger, 2001; Yan et al. 2009), and rice-based cropping systems can emit substantial amounts of N_2O (Zou et al. 2009) during the rice season itself (Wang et al. 2011).

Rice is one of the most important cereal grains and staple food crops globally and is particularly important in Asia (Meijide et al., 2011). Rice paddies contribute to the emission of the two most important GHGs: methane and nitrous oxide. Hussain et al. (2015) reported that rice fields contribute approximately 30% and 11% of CH_4 and N_2O emissions, respectively. With a linearly increasing rate of 0.26% per year during the recent few decades, the atmospheric N_2O concentration has increased by 18% compared to the preindustrial level. Methane and nitrous oxide have long atmospheric lifetimes of 12 and 114 years, respectively, and account for 20% and 7%, respectively, of global radiative forcing (Forster et al. 2007). The high global warming potential (GWP) of CH_4 and N_2O, 34 and 298 times that of CO_2 at a 100-year time horizon, makes them major contributors to climate change (IPCC 2013). In recent years, suitable management practices have been developed for achieving both improvement in rice yields and mitigation of GHG emissions, which include the development of new rice varieties (Ma et al. 2012), the application of exotic materials such as rice straw (Muller-Stover et al. 2012), the selection of appropriate cultivation methods (Liu et al. 2013) and the timing of drainage (Ma et al. 2013).

The magnitude of CH_4 emissions from rice plants is regulated by complex and dynamic interactions among the plants, environment, and microorganisms (Das and Baruah 2008). Methane produced in flooded rice soils is emitted to the atmosphere by molecular diffusion, ebullition or plant-mediated transport. Approximately 80-90% of the total CH_4 flux is emitted to the atmosphere from the rhizosphere via the rice plant (Setyanto et al. 2004). An increase in plant biomass (Eller and Frenzel 2001) and tiller number (Dubey and Singh 2000) enhanced CH_4 oxidization activity by enlarging the volume of aerenchyma and enhancing O_2 transport from the atmosphere to the rhizosphere. Ma et al. (2010) revealed that a hybrid rice variety with 50–60% higher shoot biomass emitted less CH_4 than an indica rice variety, possibly due to higher CH_4 oxidization activity.

Nitrous oxide is produced as a by-product of nitrification, denitrification, nitrifier denitrification, etc. and moisture content is a key factor governing N_2O production in soils (Wang et al. 2016). Ciarlo et al. (2007) found that N_2O emission was greatest when the water-filled pore space in soils was 80%, as most of the N_2O would be further reduced to nitrogen if the soils were wetter than this level.

Selecting a rice variety that has high productivity and low GHG emissions is crucial for improving crop yield and mitigating climate change; however, research examining the effects of rice varieties has mostly focused on CH_4 flux so far (Liou et al. 2003; Ma et al. 2012; Chen et al. 2013), with little focus on N_2O flux (Gogoi and Baruah 2012). Previous studies suggested that the effect of rice varieties on methane emissions is mostly related to rice growth performance, i.e., the number of plant tillers and plant above- and belowground biomasses (Mariko et al. 1991; Wang et al. 1997; Xu et al. 1999; Aulakh et al. 2000; Oo et al., 2016). Although many studies have found a significant positive relationship between rice biomass and methane fluxes (Singh et al. 1997; Khosa et al. 2010), a comparison of rice varieties has produced different results (Yu et al. 2013; Qin et al. 2014).

Organic residue amendments have been practised to improve soil fertility in paddy production. The organic matter in paddy fields originates from both direct by-products of rice production (such as sloughed-off root cells and root exudates) and added materials (manures and crop residues). The addition of organic carbon to the soil, whether it comes from the disposal of crop residues or as organic fertilizer, appears to be the most important factor in methane production (Khosa et al., 2010). Waterlogged conditions are ideal conditions for the decomposition of organic matter in paddy fields. The addition of cow manure as a source of organic material can also increase methane production from rice soil (Kongchum, 2005). Nitrous oxide emissions from applied fertilizer and manures can vary with differences in environmental factors (e.g., climate and soil conditions), crop factors (e.g., crop type and crop residues), and management factors (e.g., type of manure and fertilizer, application rate, time of application) (Jan et al. 2011).

Although there is a wide range of factors influencing methane emission from paddy soil (Adhya et al. 2000; Gathorne-Hardy 2013a; Yan et al. 2005), irrigation and organic amendment are the two main drivers of methane production. Under anaerobic soil conditions, methanogens (methane-producing bacteria) produce methane as a by-product of their respiration of organic matter. However, direct N_2O emissions from flooded paddy fields are minimal, as nitrification does not occur under anaerobic conditions, and consequently, neither can de-nitrification occur due to lack of NO_3^- in the soil (Qin et al. 2010). Although minimal

N_2O emissions are likely from flooded soils, some off-site (indirect) N_2O emissions are likely from irrigated rice production due to the addition of nitrogen fertilizer to fields (Alfred et al. 2013).

Myanmar ranks sixth in the world in rice production. Rice is the country's most important crop and is grown on 7.3 million ha, ~~or more than half of its arable land~~ (Myanmar Agriculture Sector in Brief 2019). The conventional rice production method ~~is~~ commonly used ~~among farmers~~ in Myanmar ~~and~~ includes transplanting old seedlings under continuous flooding conditions and the intensive use of organic fertilizers such as manure or compost. However, there is very limited information on methane emissions from the flooded rice fields of Myanmar, although more than half of the cultivated area is devoted to rice production (Oo et al. 2015; Win et al. 2020). Thus, the objectives of this study were a) to assess the effect of different types of manure amendments and rice varieties on greenhouse gas emissions and b) to determine the optimum manure application rate to increase rice yield while mitigating GHG emissions under alternate wetting and drying irrigation in paddy rice production.

.

II. Materials and Methods

The first pot experiment was conducted at the Department of Agronomy, Yezin Agricultural University (19° 45'N and 96° 6'E), Myanmar, during the wet season (June – October), 2016. A split plot design was used with 3 replications. The main factor was assigned into three categories of organic manure: 1) control (no manure), 2) compost, and 3) cow dung manure. The amount of organic manure for each treatment was based on the carbon content of the organic manure analysis that depends on the recommended amount of N was applied. The recommended chemical fertilizer amounts are 60 kg N ha^{-1}, 30 kg P_2O_5 ha^{-1}, and 20 kg K_2O ha^{-1}. The subplot factor was two types of rice varieties: Manawthukha (135 days) and IR-50 (115 days). ~~Twenty-one-day-old seedlings of each of~~ the two rice varieties were grown in a concrete pot 52.5 cm in diameter and 45 cm in height. The soil was collected from a lowland irrigated rice field. Twenty-one-day-old seedlings were transplanted with two seedlings per pot. Compost (3.9 t ha^{-1}) and cow dung (4.8 t ha^{-1}) were broadcasted at 14 days before transplanting to avoid transplanting shock due to the manure decomposition process. The soil and organic manures were analysed at the Water Science Section, Water Utilization and Agricultural Engineering Division, Department of Agricultural Research (DAR) (Table 2.1). The recommended amount of T-super (30 kg P_2O_5 ha^{-1}) was applied as basal fertilizer, and the recommended amount of potash (20 kg K_2O ha^{-1}) was applied with two split applications (as basal fertilizer and at the panicle initiation stage). The water level was

maintained at 5 cm throughout the rice growing period except during the drying period before harvest. During the rice growing season, weather data were recorded at the Department of Agronomy and are shown in Figure 2.1. The average minimum and maximum values during the rice growing season were 23.9 and 31.8°C, respectively, with 739 mm of rainfall.

The second pot experiment was conducted in a farmer's field, Madaya Township (22° 13' 0" N and 96° 7' 0" E), Myanmar, during the dry and wet seasons (February – October 2017). The pots were arranged in a split-plot design with three replications. Water management (continuous flooding (CF) and alternate wetting and drying (AWD)) was arranged as the main factor. Different rates of organic manure were assigned as subplot factors. In this study, cow dung manure was applied as an organic manure. The cow dung manure treatments (OM_0 = no cow dung, OM_1 = half of the recommended cow dung (2.5 t ha^{-1}), OM_2 = the recommended rate of cow dung (5 t ha^{-1}) and OM_3 = one and a half times the recommended rate of cow dung (7.5 t ha^{-1}), were applied seven days before transplanting. The recommended rate of cow dung manure is 5 t ha^{-1}. Each pot received the recommended fertilizer at the rates of 90 kg N ha^{-1}, 30 kg P_2O_5 ha^{-1}, and 20 kg K_2O ha^{-1}. Urea, T-super and potash were used as nutrient ~~element~~ sources. Urea was applied as three equal split applications at the active tillering, panicle initiation and heading growth stages. T-super was applied only as a basal fertilizer at one day before transplanting, and potash fertilizer was applied in two equal split applications as a basal fertilizer and at panicle initiation.

Just after transplanting, a base was placed around the plants used for gas sampling to avoid disturbing the environmental conditions around the rice plants during chamber deployment in both experiments. The base was equipped with a water seal to ensure a gas-tight closure. The base remained embedded in the soil throughout the rice growing period. Water tubes were installed in the AWD pots at a depth of 15 cm below the soil surface between the seedlings and the base just after transplanting. For AWD pots, whenever there was no water in the water tube, irrigation water was applied to a 5 cm depth above the soil surface. The irritation interval ranged from 4 to 9 days, and the amount ranged from 7 to 13 litres depending on the different rates of cow dung manure in the AWD pots. Withdrawal of water was started one week before the harvest period in all water-treated plots. During the rice growing seasons, weather data were collected from the Department of Agricultural Research, Madaya and are shown in Figure 2.2. The average minimum and maximum temperatures were 21.8°C and 35.6°C during the dry season and 26.8°C and 35.7°C during

Table 2.1 Physiochemical properties of experimental soil and organic manures used in the first pot experiment

Item	Total N%	Total P%	Total K%	O.C%	C:N
Cowdung	1.2	1.0	2.1	23.3	20.3
Compost	2.0	2.9	1.9	24.5	25:1

Item	pH	EC (dS/m)	Total N%	OM%	SO[illegible] S	Texture %		
						Sand	Silt	Clay
Soil	6.7	0.4	0.2	2.7	12	79.74	13.28	6.98

Table 2.2 Physiochemical properties of experimental soil and cowdung manure used in the second pot experiment

Analytical Item	Unit	Analytical Result	
Soil pH		7.4	Moderately alkaline
Available N	mg kg^{-1}	50	Low
Available P	mg kg^{-1}	13	Medium
Available K	mg kg^{-1}	78	Low
Total N	%	0.17	
Organic matter	%	1.8	Low
CEC	$cmol_c$ kg^{-1}	11	Low
Sand	%	87	
Silt	%	4	
Clay	%	9	
Textural class			Loamy sand
**Cowdung manure**		**Dry season**	**Wet season**
Total N	%	1.32	1.2
Organic carbon	%	16	23.3

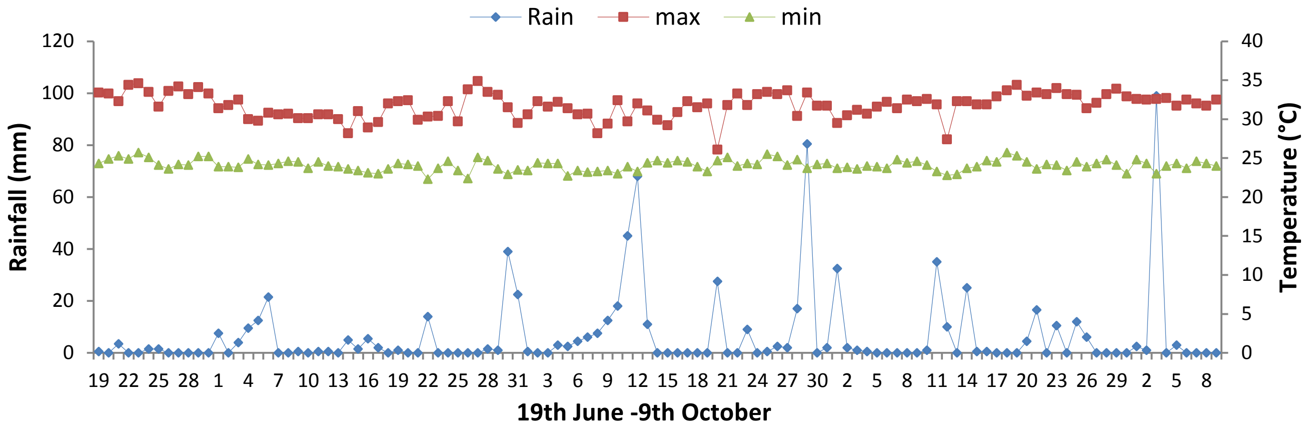

Figure 2.1 Daily rainfall, maximum and minimum temperatures in Yezin Agricultural University, Myanmar during wet season, 2016

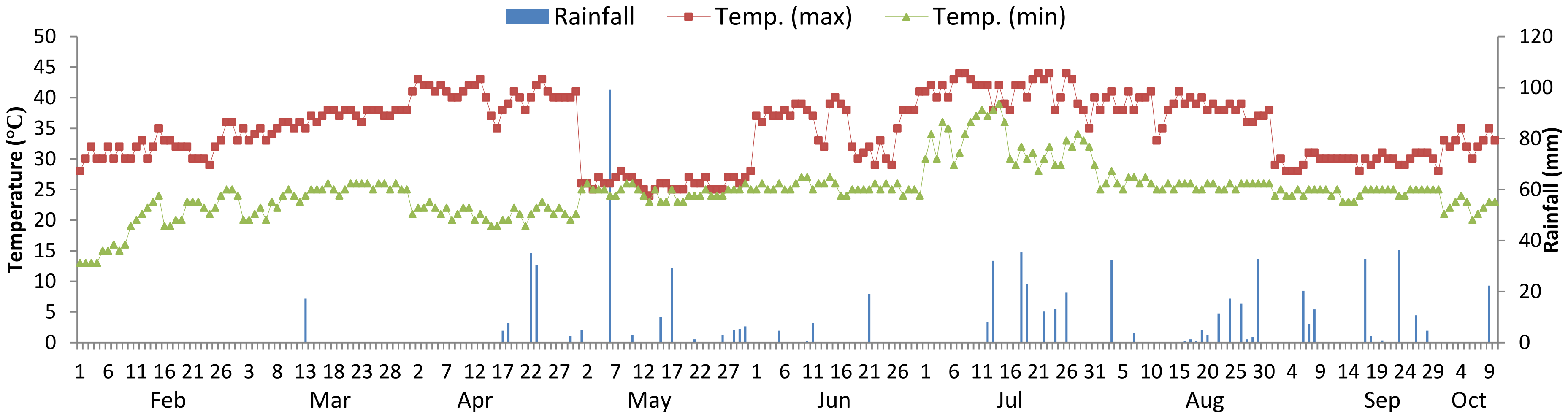

Figure 2.2 Daily rainfall, maximum and minimum temperatures in Madaya township, Myanmar during dry and wet seasons, 2017

the wet season, respectively. The total rainfall amounts were 201.7 mm during the dry season and 420.6 mm during the wet season.

Gas sample collection, analysis and calculation

A two-bonded chamber with a total capacity of 77 L (93 cm height) was used for collecting the gas sample. To thoroughly mix the gases in the chamber, the chamber was equipped with a small 12 volt DC fan connected with three 9-volt dry cells (Ali et al. 2015). For CH_4 calculation, the temperature was recorded with a digital thermometer (TT-508 Tanita, Tokyo, Japan). To compensate for the air pressure changes between the increased temperature and gas sampling, an air buffer bag (1-L Tedlar bag) was attached to the chamber. The silicon rubber tube, together with the soft vinyl tube attached with three-way stop corks, was inserted airtight into a hole on the chamber. The gas sample was taken with an airtight 50 ml syringe by connecting it to the three-way stop cock and then transferred to a 20 ml vacuum glass vial, which was evacuated.

Gas sampling was performed at 7-day intervals starting from 1 day after transplanting until harvest by the closed chamber method in pot experiment 1. In pot experiment 2, the first two gas samplings were performed at one-week intervals after transplanting, and later gas samplings were performed at 10-day intervals. The gas samples were collected from 9:00 am until 12:00 am and three times (0, 15, 30 min) for each treatment for gas flux calculation.

Methane and N_2O concentrations were analysed with a gas chromatograph (GC 2014, Shimadzu Corporation, Kyoto, Japan) equipped with a flame ionization detector (FID) and an electron capture detector (ECD). The amount of CH_4 and N_2O fluxes was calculated by using the following equation:

$$Q = (V/A) \times (\Delta c/\Delta t) \times (M/22.4) \times (273/K)$$

where Q = the flux of gas (mg m^{-2} min^{-1})

V = the volume of the chamber (m^3)

A = the base area of the chamber (m^2)

($\Delta c/\Delta t$) = the rate of increase or decrease in the gas concentration (mg m^{-3}) per unit time (min)

M = the molar weight of the gas

K = Kelvin temperature of the air temperature inside the chamber

Total emissions were calculated by multiplying the daily gas flux at each measurement for the time interval and summing up the values for the growing period. In this study, the IPCC factors were used to calculate the combined GWPs for 100 years (GWP= ($25\times CH_4$) +

(298×N_2O)) in kg CO_2-equivalents ha^{-1} (IPCC 2007) for CH_4 and N_2O. The yield was recorded from each pot. The grains were threshed, cleaned and sun-dried. Yields were adjusted at 14% moisture by using the following formula to remove the error due to the different moisture content, and grain moistures were measured by using a grain moisture metre (model: GMK-303RS).

Adjusted grain weight at 14% moisture level = A x W

where A = Adjustment coefficient

W = Weight of harvested grains

$$A = \frac{100\text{-moisture\%}}{86}$$

The greenhouse gas intensity (GHGI) was calculated by dividing the GWP by the rice grain yield (Xiong et al. 2015; Pittelkow et al. 2014; Van et al. 2010).

$$GHGI = GWP/\text{grain yield } (kg\ CO_2\text{-eqv } kg^{-1} \text{ grain})$$

Statistical analysis

The data were analysed by using Statistix (version 8.0). Mean comparisons were performed by least significant difference (LSD) test at the 5% level.

III. Results

First pot experiment during the wet season, 2016

Methane emission: During the early growth stage, low CH_4 emission was observed for both varieties until 36 DAT, and then CH_4 emission flux gradually increased with some fluctuations until harvest (Figure 3.1). The mean cumulative CH_4 emissions from the control, compost, and cow dung treatments were 625.1, 665.8, and 565.8 kg CH_4 ha^{-1}, respectively. Although the effect was not significant, cow dung amendment reduced cumulative CH_4 emissions by 9.5% compared with the control. When comparing the two rice varieties, the total cumulative CH_4 emissions were significantly higher in the Manawthukha variety (759.1 kg CH_4 ha^{-1}) than in the IR-50 variety (478.7 kg CH_4 ha^{-1}) ($P<0.0106$) (Table 3.1). The IR-50 variety reduced cumulative CH_4 emissions by 36.9% compared to the Manawthukha variety. During the rice growing season, cumulative CH_4 emissions were higher in later growth stages than in the vegetative growth stage.

Nitrous oxide emission: High nitrous oxide emission was observed during very early growth until 15 DAT in both varieties. After that, a small amount of N_2O emission was found until harvest (Figure 3.2). Although there was no significant difference in N_2O emissions among the manure treatments, higher emissions (2.25 kg ha^{-1}) were recorded from the control (no

manure) plot compared to the compost (1.74 kg ha^{-1}) and cow dung plots (1.49 kg ha^{-1}) (Table 3.1). Cow dung manure reduced cumulative N_2O emissions by 33.8% compared with the control. There was no significant difference in cumulative N_2O emissions among the tested varieties: Manawthukha variety (1.81 kg ha^{-1}), IR-50 (1.85 kg ha^{-1}) (Table 3.1).

Global warming potential (GWP): GWP was not significantly different among manure treatments, although higher GWP (17.2 t CO_2-equ. ha^{-1}) was observed in the compost treatment followed by the control (16.3 t CO_2-eq. ha^{-1}) and cow dung treatment (14.6 t CO_2-eq. ha^{-1}) (Figure 3.3a). There was a significant difference in GWP among the varieties (Pr<0.0110). A higher GWP was observed for Manawthukha (19.5 t CO_2-eq.ha^{-1}) than for IR-50 (12.5 t CO_2-eq. ha^{-1}).

Rice yield: Grain yield was significantly affected by the manure treatments (P<0.0172). The highest grain yield (122.4 g $plant^{-1}$) was recorded from the control (no manure), followed by the compost (102.7 g $plant^{-1}$) and cow dung treatments (79.7 g $plant^{-1}$). However, there was no significant difference in grain yield between the varieties Manawthukha (121.5 g $plant^{-1}$) and IR-50 (81.6 g $plant^{-1}$).

Greenhouse gas intensity: Greenhouse gas intensity was affected by manure management (P<0.0617) (Table 3.1). Cow dung manure gave the highest GHGI (4.3 kg CO_2- eqv kg^{-1} grain), followed by compost (3.8 kg CO_2- eqv kg^{-1} grain), and the control gave the lowest GHGI (2.9 kg CO_2- eqv kg^{-1} grain). The two rice varieties were not significantly different in GHGI values. However, across manure management treatments, higher GHGI values were found in Manawthukha than in IR-50 (Figure 3.3b). There was no interaction between manure and rice varieties on the GHGI.

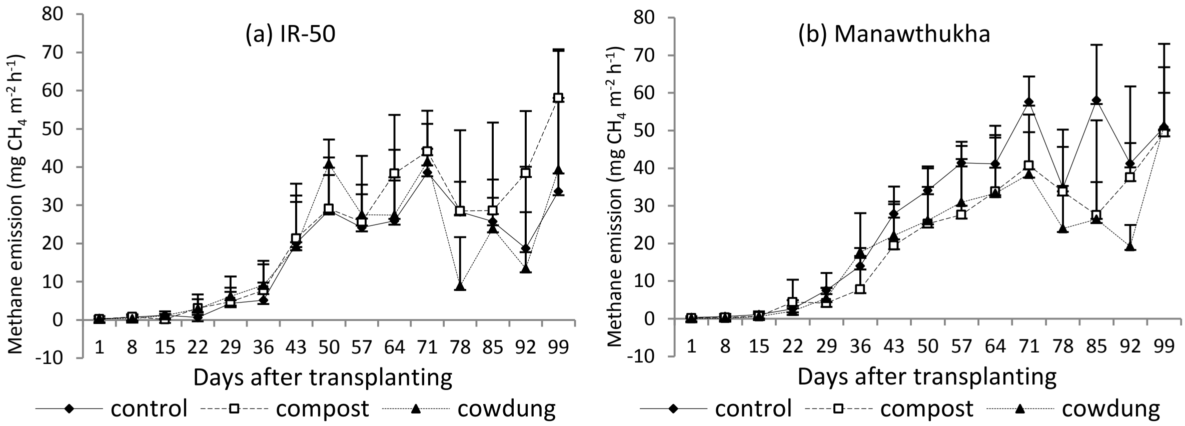

Figure 3.1 Methane emission of rice varieties at Yezin Agricultural University during the wet season, 2016. Mean value±standard deviation (n=3).

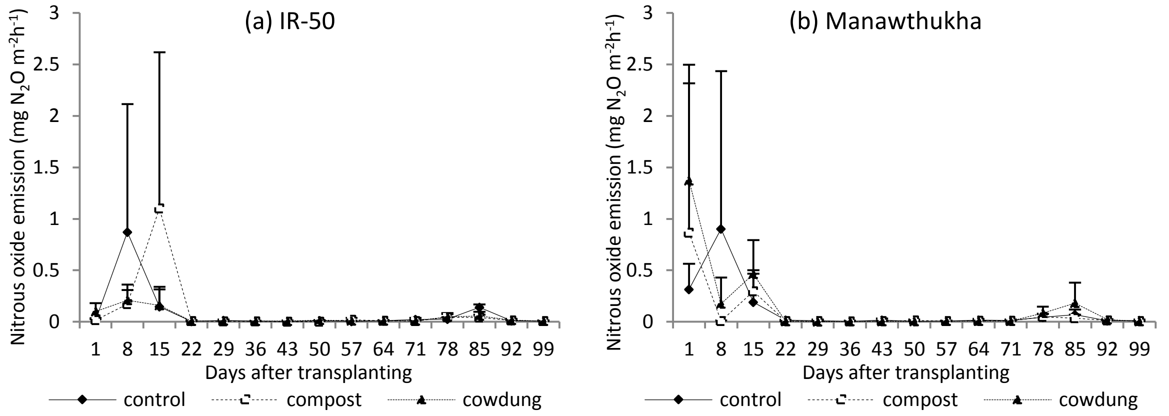

Figure 3.2 Nitrous oxide emission of rice varieties at Yezin Agricultural University during the wet season, 2016. Mean value±standard deviation (n=3).

Table 3.1 Effects of manure and rice variety on greenhouse gases emission and grain yield of rice during wet season, 2016

Treatment	CH_4 (kg ha^{-1})	N_2O (kg ha^{-1})	Grain yield (g plant^{-1})	GHGI (kg CO_2-eqv kg^{-1} grain)
Manure				
Control	625.1	2.25	122.4 a	2.9 b
Compost	665.8	1.74	102.7 ab	3.8 ab
Cowdung	565.8	1.49	79.7 b	4.3 a
LSD $_{0.05}$	147.60	4.4563	23.037	1.18
Variety				
Manawthukha	759.1 a	1.81	121.5	3.8
IR-50	478.7 b	1.85	81.6	3.5
LSD $_{0.05}$	187.41	1.2726	40.545	0.53
Pr>F				
Manure	0.2788	0.8934	0.0172	0.0617
Variety	0.0106	0.9396	0.0526	0.2296
Manure*Variety	0.6541	0.2461	0.7395	0.9367
CV_a (%)	14.88	151.86	14.15	20.17
CV_b (%)	26.25	60.26	34.60	12.55

Within each column, values with different alphabets indicate significant differences among the treatments at 5% of LSD test.

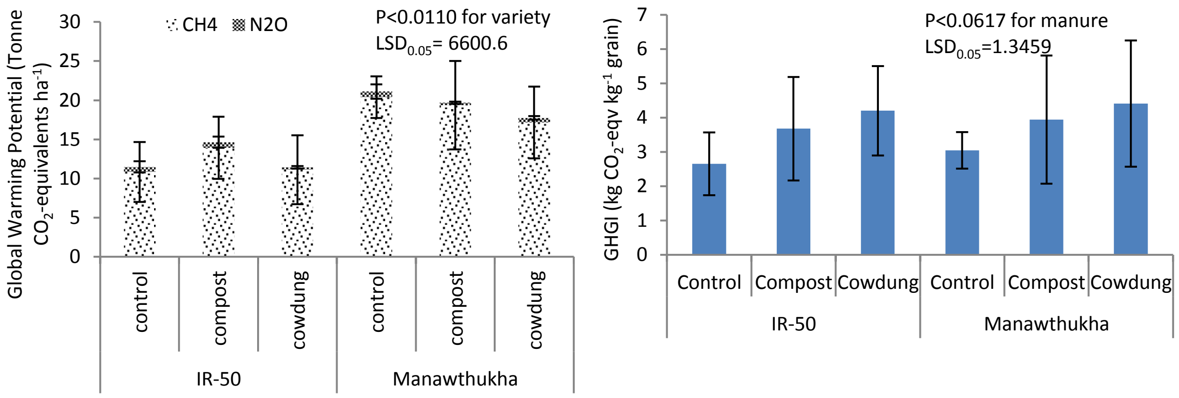

(a) Global warming potential (b) Greenhouse gas intensity

Figure 3.3 Effect of organic manure and rice varieties on (a) global warming potential and (b) greenhouse gas intensity during wet season, 2016. Mean value±standard deviation (n=3).

Second pot experiment during the dry and wet seasons, 2017

Methane emission: The seasonal methane emissions of rice are shown in Figure 3.4 (dry season) and Figure 3.5 (wet season). In the dry season, high methane emissions were observed in the early growth stage and then decreased until harvest under both water regimes. In the wet season, a slight increase in emissions was recorded in the early growth stage, emissions peaked in the middle stage, and gradually decreased until harvest. There were significant differences in cumulative CH_4 emissions among water management practices ($P<0.05$) (Table 3.2). Higher cumulative CH_4 emissions were observed under CF than AWD. Despite no significant difference among the cow dung manure rates, a generally higher amount of cow dung manure produced more methane emissions than lower rates.

Nitrous oxide emission: The seasonal nitrous oxide emissions of rice are shown in Figure 3.6 (dry season) and Figure 3.7 (wet season). Relatively high nitrous oxide emissions were found in the early growth stage, and reduced emissions were found in the later growth stage in both seasons. There were no significant differences in nitrous oxide emissions among the tested factors (water and manure) in either season. However, numerically higher N_2O emissions were observed under AWD than CF (Table 3.2). AWD increased N2O emissions by 124% compared to CF.

Global warming potential (GWP): The GWP was significantly different among water management practices ($P<0.05$). A higher GWP (66.3 t CO_2-eq ha^{-1}) was observed under CF than under AWD (20.1 t CO_2-eq ha^{-1}) in the dry season (Figure 3.8a), and a higher GWP (40.0 t CO_2-eq ha^{-1}) was observed under CF than AWD (13.7 t CO_2-eq ha^{-1}) in the wet season (Figure 3.8b). Generally, the large application of cow dung manure resulted in a higher GWP in both seasons. The different rates of cow dung manure had no significant effect on the GWP in either season.

Rice yield: In this pot experiment, the grain yield was not significantly affected by water management and manure amendment in either rice season (Table 3.3). However, higher grain yields per plant were recorded with AWD than CF in both seasons. The manure rates had no significant effect on rice yield.

Greenhouse gas intensity: Greenhouse gas intensities were significantly different among the water management practices in both seasons ($P<0.05$) (Table 3.3). Significantly higher GHGI values were found under CF than under AWD in both seasons (Figure 3.9). No significant differences were found in GHGI values among the manure management practices. However, the higher amount of cow dung manure affected the GHGI values under CF irrigation, but the effect of manure was suppressed by AWD.

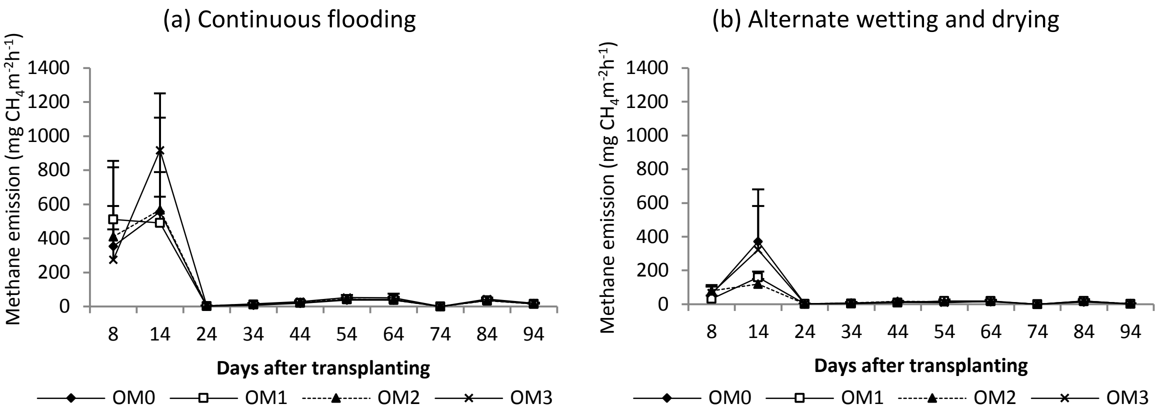

Figure 3.4 Methane emission from rice under (a) continuous flooding and (b) alternate wetting and drying during the dry season, 2017. Mean value±standard deviation (n=3).

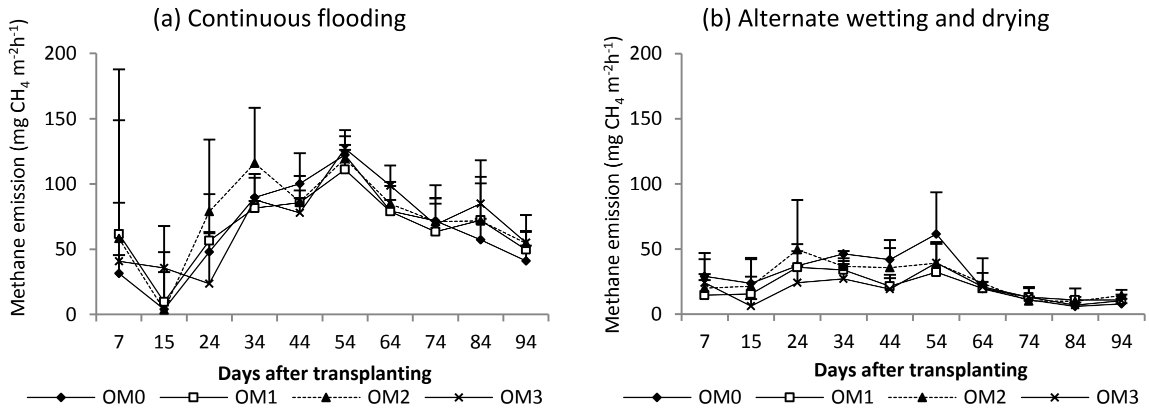

Figure 3.5 Methane emission from rice under (a) continuous flooding and (b) alternate wetting and drying during the wet season, 2017. Mean value±standard deviation (n=3).

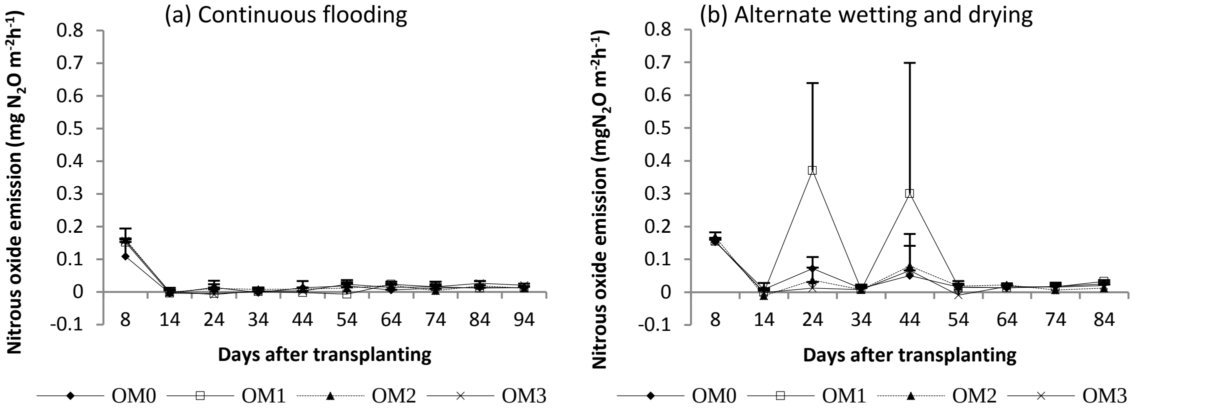

Figure 3.6 Nitrous oxide emission from rice under (a) continuous flooding and (b) alternate wetting and drying during the dry season, 2017. Mean value±standard deviation (n=3).

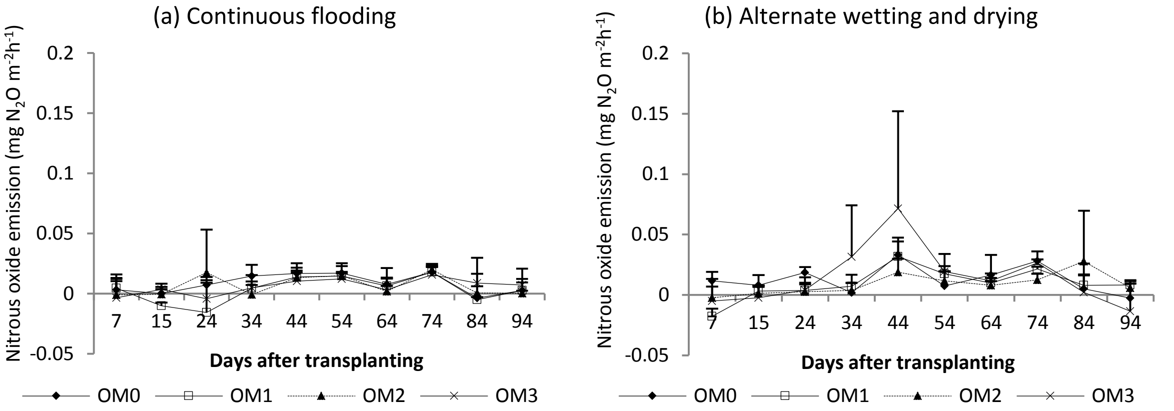

Figure 3.7 Nitrous oxide emission of rice (a) continuous flooding and (b) alternate wetting and drying during the wet season, 2017. Mean value±standard deviation (n=3).

Table 3.2 Mean effects of water and manure management on greenhouse gases emission of rice during dry and wet seasons, 2017

Treatment	Cumulative methane emission (kg CH_4 ha^{-1})		Cumulative nitrous oxide emission (kg N_2O ha^{-1})	
	Dry season	Wet season	Dry season	Wet season
Water				
CF	2645.6	1597.6 a	0.4695	0.1459
AWD	792.2	542.7 b	1.1072	0.2737
LSD $_{0.05}$	1967.1	737.19	1.1021	0.2528
Manure				
OM_0 (0 t ha^{-1})	1692.7	1082.2	0.6256 ab	0.2434
OM_1 (2.5 t ha^{-1})	1619.2	1016.9	1.2890 a	0.1604
OM_2 (5 t ha^{-1})	1595.7	1161.2	0.6501 ab	0.1865
OM_3 (7.5 t ha^{-1})	1968.0	1020.3	0.5887 b	0.2488
LSD $_{0.05}$	905.3	372.1	0.6994	0.1781
Pr>F				
Water	0.0558	0.0254	0.1305	0.1617
Manure	0.7974	0.8147	0.1440	0.6502
Water x Manure	0.7822	0.7545	0.0844	0.8041
CV_a (%)	65.15	39.22	79.59	68.60
CV_b (%)	41.87	27.64	70.53	67.51

Within each column, values with different alphabets indicate significant differences among the treatments at 5% of LSD test.

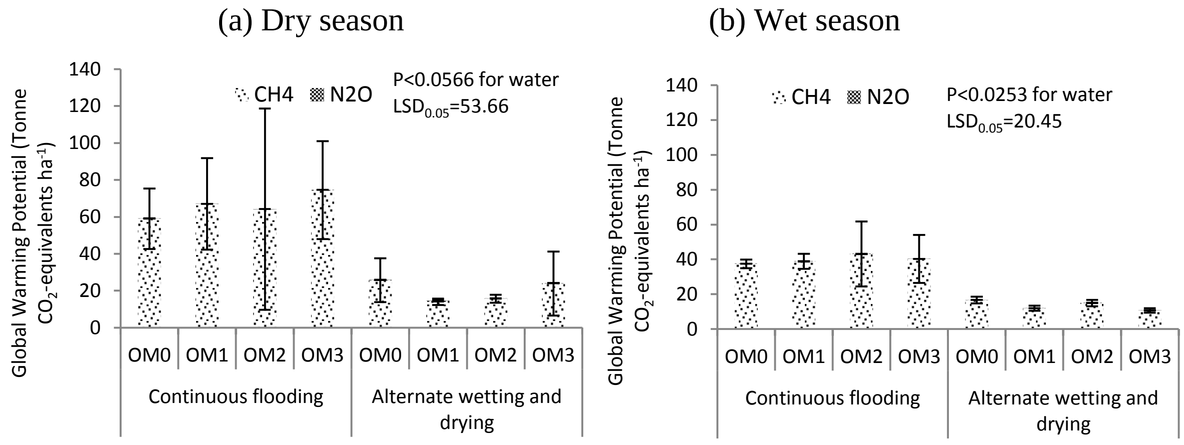

Figure 3.8 Effect of water and cowdung manure management on global warming potential of potted rice during dry (a) and wet (b) seasons, 2017. OM_0-no cowdung, OM_1-cowdung 2.5 t ha^{-1}, OM_2- cowdung 5.0 t ha^{-1}, OM_3- cowdung 7.5 t ha^{-1}. Mean value±standard deviation (n=3).

Table 3.3 Mean effects of water and manure management on rice yield during dry and wet seasons, 2017

Treatment	Yield (g $plant^{-1}$)		GHGI (kg CO_2-eqv kg^{-1} grain)	
	Dry season	Wet season	Dry season	Wet season
Water				
CF	175.06	180.88	7.47	4.34 a
AWD	177.73	183.47	2.19	1.47 b
$LSD_{0.05}$	34.16	11.19	5.71	1.94
Manure				
OM_0 (0 t ha^{-1})	181.54	183.70	4.52	2.94
OM_1 (2.5 t ha^{-1})	171.79	178.81	4.72	2.75
OM_2 (5 t ha^{-1})	178.08	179.20	4.39	3.17
OM_3 (7.5 t ha^{-1})	174.16	187.01	5.67	2.75
$LSD_{0.05}$	12.45	22.75	2.46	1.02
Pr>F				
Water	0.7684	0.4246	0.0579	0.0240
Manure	0.3742	0.8384	0.6711	0.7780
Water x Manure	0.1292	0.7134	0.5745	0.7548
CV_a (%)	11.02	3.50	67.38	38.09
CV_b (%)	5.61	9.93	40.44	27.91

Within each column, values with different alphabets indicate significant differences among the treatments at 5% of LSD test.

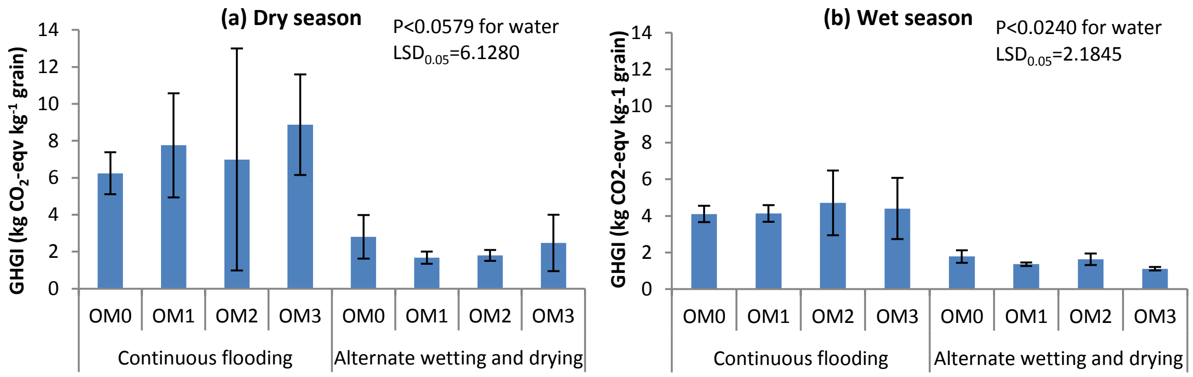

Figure 3.9 Effect of water and cowdung manure management on greenhouse gas intensity of potted rice during dry (a) and wet (b) seasons, 2017. OM_0-no cowdung, OM_1- cowdung 2.5 t ha^{-1}, OM_2- cowdung 5.0 t ha^{-1}, OM_3- cowdung 7.5 t ha^{-1}. Mean value±standard deviation (n=3).

IV. Discussion

Effect of manure and rice varieties on greenhouse gas emissions, global warming potential and rice yield

Methane production and emissions from flooded paddies are highly affected by the addition of organic matter (Jean and Pierre 2001). The higher CH_4 emission in this study was found in later growth stages (Figure 3.1) because the CH_4 emission was associated with higher soil organic matter turnover due to increased microbial activities, decomposition of decaying plant residues from fallen leaves and root turnover, and higher availability of root exudates in the rhizosphere (Oo et al. 2013). Although there was no significant difference based on manure management, cow dung manure reduced methane emissions compared to the control (no manure) and compost (Table 3.1) because the incorporation of humified organic matter could minimize CH_4 emissions from rice fields with concomitant benefits of increased soil fertility and crop productivity (Khosa et al. 2010). This study also agrees with that of Oo et al. (2016), who reported that the lower CH_4 emissions from pots treated with well-decomposed cattle manure could be explained by fewer carbon substrates with a depletion of potential CH_4 precursors resulting from the preceding fermentation. In contrast to CH_4 emissions, high N_2O emissions were recorded in the early growth stage, and small amounts of emissions were recorded in the later growth stage (Figure 3.2). This could be due to the rapid nitrification and denitrification affected by certain weather conditions, e.g., rainfall and temperature (Figure 2.1), and availability of soil nitrogen (Table 2.1). After emission peaked initially, the rates of N_2O emission were generally low due to continuous flooding. Another study reported that the consistently low soil redox potential under continuous flooding resulted in more complete denitrification and thus reduced N_2O emissions (Oo et al., 2018). The control (no manure) treatment resulted in higher N_2O emissions than the compost and cow dung treatments. This is explained by the composition of the manure and residues, specifically the high organic carbon percent and high carbon nitrogen ratio (Table 2.1). This study agrees with that of Shan and Yan (2013), who reported that N_2O emissions were significantly reduced by crop residue return combined with synthetic N fertilizers compared with emissions from treatments that only received synthetic N fertilizer.

The selection of suitable rice varieties might play a significant role in regulating CH_4 emissions from rice fields (Oo et al., 2018). The results of this study showed that there was a significant difference between the tested rice varieties (Figure 3.1 and Table 3.1). The result was in agreement with other findings, which highlighted that there were substantial

differences in the rates of CH_4 emission among different rice varieties (Kumar and Viyol 2009, Koga and Tajima 2011, Oo et al., 2016). As ninety percent of methane emissions to the atmosphere are through rice plants (Wang et al., 2000), the Manawthukha variety, which has a longer growth duration and thus higher biomass, emitted more CH_4 than IR-50, which has a shorter growth duration and lower biomass. Previous studies (Cai et al. 2000; Satyanto et al. 2000; Yang et al. 2010; Oo et al. 2015) have also reported that the CH_4 flux from late maturing rice is higher than that from early maturing rice. According to this research finding, a shorter growth duration (IR-50) variety can be used to reduce methane emissions. When compared to the two rice varieties, IR-50 resulted in higher N_2O emissions than the Manawthukha variety. This could be due to the favourable effect of root exudates of IR-50 on the nitrification process in the soil.

There was no interaction between manure and rice varieties. This result indicated that the effect of rice varieties on greenhouse gas emissions (CH_4 and N_2O) was not affected by the application of manure.

Cow dung manure resulted in a 10.4% reduction in GWP compared to the control (no manure), while an increased GWP (5.5%) was found for the compost compared to the control. Manawthukha had a higher GWP (56%) than IR-50. These results were supported by Zheng et al. (2014), who reported that yield-scaled GWP at 80-90 days of growth duration after transplanting was 87% higher than that at 70-80 days of growth duration after transplanting. Feng et al. (2013) also reported that yield-scaled GWP in late rice in a double-rice cropping system was 73% higher than that for varieties with 90-100 days of growth duration after transplanting.

A significantly high grain yield of rice was recorded from the control due to the mineralization of indigenous soil organic matter and inorganic fertilizer (Table 3.1). Oo et al. (2010) reported that a high grain yield under inorganic sources of nutrients might be due to the immediate release and availability of nutrients. This study was also in agreement with that of Banik et al. (2006), who stated that organic fertilizer with inorganic fertilizer increased the fertilizer use efficiency and improved the physical and chemical properties of soil, which would be a reason for the increased yield. Relative to IR-50, Manawthukha had a higher grain yield by 48.9% and increased GHGI by 8.6% because of its longer growth duration and grain filling period. Therefore, considering the GWP and GHGI, IR-50 could be recommended for rice cultivation in the study area of Myanmar.

Effect of water management and manure application on greenhouse gas emissions, global warming potential and rice yield

In the dry season, high methane emissions were observed in the early growth stage and then decreased until harvest in both water regimes (Figure 3.4). The early increase in CH_4 emission was due to the indigenous soil carbon content and availability of substrates, and the decrease in the later growth stage was due to the senescence of older leaves and non-availability of substrate as the crop approached maturity (Das and Adhya 2014; Borah and Baruah 2016b; Baruah et al. 2018). In the wet season, a slight increase in emission was recorded in the early growth stage, and emission was highest in the middle stage and gradually decreased until harvest (Figure 3.5). A higher rate of CH_4 production is attributed to the availability of organic substrates from the previous crop residues in the form of plant-derived C through processes such as root exudation and release of fallen leaves and intensively reduced conditions in the rice rhizosphere (Kell 2012; Oo et al. 2018). The gradual decrease in CH_4 emission was due to the decomposition and non-availability of substrates depending on weather conditions (Figure 2.2). Higher cumulative CH_4 emissions were observed under CF than under AWD because the anoxic conditions increased the methanogen population and favoured methane production. The methane emission was higher in the dry season than in the wet season because it depended on the availability of indigenous soil carbon and was restricted by the environment (pot). Relatively high nitrous oxide emissions were observed in the early growth stage, and reduced emissions were observed in the later growth stage in both seasons (Figure 3.6 and 3.7). This was due to the indigenous soil nitrogen content in the early stage, depletion of nutrients, factors inhibiting the nitrification and denitrification processes (e.g., moisture and weather, see Figure 2.2) and the available nitrogen content. Higher N_2O emissions were observed under AWD than CF in both seasons (Hou et al. 2000; Jain et al. 2014; Oo et al. 2018). AWD increased N2O emissions by 124% compared to CF for both seasons. Similar to the CH_4 emission pattern, higher N_2O emissions were observed in the dry season than in the wet season because of the soil nitrogen availability and the controlled soil environment (pot).

Higher rates of cow dung manure resulted in higher CH_4 emission because it provided carbon substrates to methanogens. Generally, higher N_2O emission was observed with higher rates of cow dung manure application. This was associated with the high total N% of manure (Table 2.2) and nitrification and denitrification processes of manure under favourable soil-environmental factors, specifically soil moisture.

No interactive effect between water management and the application of different cow dung manure rates was found on CH_4 emissions in either season. However, the effect of the application of different cow dung manure rates on N_2O emissions was influenced by alternate wetting and drying irrigation practices in the dry season but not in the wet season. Therefore, the nitrification and denitrification processes of nutrients are influenced by the soil moisture content.

A higher GWP was observed under CF in accordance with the higher methane emissions in both seasons (Figure 3.8). Methane emission mainly contributes to the GWP from paddy production. Many studies (Yan et al. 2005; Kurosawa et al. 2007; Itoh et al. 2011; Pittelkow et al. 2013; Sander et al. 2015; Tarlera et al. 2016, Oo et al. 2018) reported that N_2O emissions contribute much less to the global warming potential than those of CH_4. Therefore, the water regime in paddy production is the main factor controlling CH_4 emissions from rice fields (Yan et al. 2005; Oo et al. 2018; Wu et al. 2018). Generally, it can be concluded that the higher application of cow dung manure resulted in a higher GWP because it provided more substrates for methanogens for the production of methane; however, this effect is also weather dependent.

AWD resulted in a higher grain yield per plant than did CF in both seasons (Table 3.3) because it strengthened the air exchange between the soil and atmosphere and supplied sufficient oxygen to the root system to accelerate soil organic matter mineralization, all of which should increase soil fertility and favour rice growth (Dong et al. 2012; Tan et al. 2013). Yang and Zhang (2010) reported an increase in paddy yield under AWD due to the increase in the proportion of productive tillers. Zhang et al. (2009) also indicated an irrigation water saving of 35% under AWD with a 10% yield increase relative to that under CF. Liu et al. (2013), Ye et al. (2013), and Djaman et al. (2018) found that grain yield increased with reduced water input by AWD. Grain yield was not affected by the different rates of cow dung manure application because its decomposition was influenced by biotic and soil-environmental factors. Relative to the CF water management, AWD produced comparable grain yields, decreased only by 1.5% but resulted in a decreased GHGI by 69% (Table 3.3). This suggests that by adopting alternate wetting and drying irrigation, it would be possible to achieve the dual goals of maintaining productivity while minimizing the global warming potential of rice cultivation.

V. Conclusion

For sustainable agriculture, organic manures should be added at a reasonable amount to increase rice yield and reduce greenhouse gas emissions. From our findings, the application of cow dung manure can be recommended in paddy production since it mitigated the global warming potential compared to that of the control and compost groups, although it resulted in a lower yield potential. Additionally, the short duration rice variety had a lower GWP and a lower GHGI value while maintaining the potential rice yield. Manure-induced greenhouse gas emissions were suppressed by AWD irrigation practices, with significant GHGI values. Thus, short duration varieties are highly recommended with AWD irrigation and application of 5 t ha^{-1} cow dung manure to reduce greenhouse gas emissions and maintain rice yield under the soil-environmental conditions of Myanmar. In our study, there was no relationship between GWP and rice yield. Therefore, the choice of rice varieties should be combined with soil-environmental factors and cultivation systems to mitigate greenhouse gas emissions while increasing rice yields for sustainable rice production.

Acknowledgements

We thank Sonoko D. Bellingrath-Kimura and Aung Zaw Oo for technical advice and editing a previous manuscript version. IPCC and Cuomo Foundation are highly appreciated for their financial support to carry out these researches.

Reference

Adhya, T.K., Bharati, K., Mohanty, S.R., Ramakrishnan, B., Rao, V.R., Sethunathan, N., and Wassmann, R. 2000. Methane emission from rice fields at Cuttack, India. Nutrient Cycling in Agroecosystems, 58, 95-105.

Alfred, G.H., Duv, N.R., Venkatanarayana, M., Barbara, H.W. 2013. A Life Cycle Assessment (LCA) of greenhouse gas emissions from SRI and flooded rice production in SE India. Taiwan Water Conservancy, 61:4, 110-125.

Ali MA, Kim PJ and Inubushi K, Mitigating yield-scaled greenhouse gas emissions through combined application of soil amendments: A comparative study between temperate and subtropical rice paddy soils. *Science of the Total Environment* 529:140–148 (2015).

Aulakh MS, Bodenbender J, Wassmann R, Rennenberg H. 2000. Methane transport capacity of rice plants, II. Variations among different rice cultivars and relationship with morphological characteristics. NutrCycling Agroecosyst. 58:367–375.

Banik, P., P.K. Ghosal, T.K. Sasmal, S. Bhattacharya, B.K. Sarkar and D.K. Bagchi, 2006. Effect of organic and inorganic nutrients for soil quality conservation and yield of rainfed low land rice in sub-tropical plateau region. J. Agron. Crop Sci., 192: 331-343.

Baruah, K.K., Gorh, D., Gogoi, N. 2018. Analysis of greenhouse gas (Methane and Nitrous oxide) emission and global warming potential from rice fields: with reference to biological mitigation of climate change. SDRP Journal of Earth Sciences & Environmental Studies, 3:2, 1-13.

Borah L, Baruah KK (2016b) Effects of foliar applica-tion of plant growth hormone on methane emission from tropical rice paddy. Agriculture, Ecosystem and Environment 233:75–84.

Burney, J.A., Davis, S.J., Lobell, D.B., 2010. Greenhouse gas mitigation by agricultural intensification. Proc. Nat. Acad. Sci. U. S. A. 107, 12052–12057.

Cai, Z.C., Tsuruta, H., Minami, K., 2000. Methane emission from rice fields in China: measurements and influencing factors. J. Geophys. Res. 105 (D13), 17231–17242.

Chen, J, Qin, G, Wang, J, Yu, J, Shen, B, Li, S, Ren, Y, Zuo, L, Shen, W, Das, B. 2013. One-step fabrication of sub-10-nm plasmonic nanogaps for reliable SERS sensing of microorganisms. Biosens Bioelectron 44, 191-197.

Ciarlo, E., Conti, M., Bartoloni, N., Rubio, G. 2007. The effect of moisture on nitrous oxide emissions from soil and the N2O/(N2O+N2) ratio under laboratory conditions. Biol. Fertil. Soils 43, 675-681.

Das S, Adhya TK (2014) Effect of combine applica-tion of organic manure and inorganic fertilizer on me-thane and nitrous oxide emissions from a tropical flooded soil planted to rice. Geoderma 213:185192.

Das, K. and Baruah, K. K. 2008. Association between contrasting methane emissions of two rice (*Oryza sativa* L.) cultivars from the irrigated agroecosystem of northeast India and their growth and photosynthetic characteristics. Acta Physiologiae Plantarum, 30, 569-578.

Djaman, K., Mel, V.C., Diop, L., Sow, A., El-Namaky, R., Manneh, B., Saito, K., Futakuchi, K., and Irmak, S. 2018. Effects of AlternateWetting and Drying Irrigation Regime and Nitrogen Fertilizer on Yield and Nitrogen Use Efficiency of Irrigated Rice in the Sahel. Water, 10, 711.

Dong, N.M.; Brandt, K.K.; Sørensen, J.; Hung, N.N.; Hach, C.V.; Tan, P.S.; Dalsgaard, T. Effects of alternating wetting and drying versus continuous flooding on fertilizer

nitrogen fate in rice fields in the Mekong Delta, Vietnam. *Soil Biol. Biochem*. 2012, *47*, 166–174.

Dubey SK, Singh JS (2000) Spatio-temporal variation and effect of urea fertilization on methanotrophs in a tropical dryland rice field. Soil Biol Biochem 32:221–226

Eller G, Frenzel P (2001) Changes in activity and community structure of methane oxidizing bacteria over the growth period of rice. Appl Environ Microbiol 67:2395–2403.

Feng, J. F., Chen, C. Q., Zhang, Y., Song, Z. W., Deng, A. X., Zheng, C. Y., and Zhang,W. J.: Impacts of cropping practices on yield-scaled greenhouse gas emissions from rice fields in China: a meta-analysis, Agr. Ecosyst. Environ., 164, 220–228, 2013.

Forster P, Ramaswamy V, Artaxo P, Berntsen T, Betts R, Fahey DW, Haywood J, Lean J, Lowe DC, Myhre G, Nganga J, Prinn R, Raga G, Schulz M, Van Dor-land R. 2007. Changes in atmospheric constituents and in radiative forcing. In: Solomon S, Qin D, Man-ning M, Chen Z, Marquis M, Averyt K, Tignor M, Miller H (Eds) Climate Change 2007: the physical science basis. Contribution of Working Group I to the Fourth Assessment Report of the Intergovernmental Panel on Climate Change. Cambridge University Press: UK and New York pp 129–234.

Gathorne-Hardy, A. 2013a. Greenhouse gas emissions from rice, Working paper for South Asia Program, School of Interdisciplinary Area Studies, Oxford University, UK. http://www.southasia.ox.ac.uk/sites/sias/files/documents/GHG%20emissions%20from%20rice%20-%20%20working%20paper.pdf

Gogoi, T.K. and Baruah, D. C. 2012. Performance and energy analyses of a diesel engine fuelled with Koroch seed oil methyl ester and its diesel fuel blends. International Journal of Energy Technology and policy, 7:5/6, 433-454.

Hou, A.X., Chen, G.X., Wang, Z.P., Van Cleemput, O., Patrick Jr., W.H., 2000. Methane and nitrous oxide emissions from a rice field in relation to soil redox and microbiological process. Soil Sci. Soc. Am. J. 64, 2180–2186.

Hussain S, Peng S, Fahad S, Khaliq A, Huang J, Cui K, Nie L. 2015. Rice management interventions to mitigate greenhouse gas emissions: a review. Environ. Sci. Pollut. Res. 22:3342-3360. PMid:25354441 View Article PubMed/NCBI

IPCC. Climate change 2007: synthesis report *Contribution of Working Groups I, II and III to the Fourth Assessment Report of the Intergovernmental Panel on Climate Change*, ed. By Pachauri, RK and Reisinger A. IPCC, Geneva, Switzerland pp. 104 (2007).

IPCC: Intergovernment Panel on Climate Change. 2013. Fifth Assessment Report (AR5) Contribution of Working Group III to Climate Change 2014: Miti-gation of Climate Change.

Itoh, M., Sudo, S., Mori, S., Saito, H., Yoshida, T., Shiratori, Y., Suga, S., Yoshikawa, N., Suzue, Y., Mizukami, H., Mochida, T., Yagi, K., 2011. Mitigation of methane emissions from paddy fields by prolonging midseason drainage. Agric. Ecosyst. Environ. 141, 359–372.

Jain, N., Dubey, R., Dubey, D.S., Singh, J., Khanna, M., Pathak, H., Bhatia, A., 2014. Mitigation of greenhouse gas emission with system of rice intensification in the Indo-Gangetic Plains. Paddy Water Environ. 12 (3), 355–363.

Jan, P.L., Gerard, L.V., Wim, de V., Johannes, K. 2011. Differentiation of nitrous oxide emission factors for agricultural soils. Environmental Pollution, 159, 3215-3222.

Jean, L.M. and R. Pierre. 2001. Production, oxidation, emission and consumption of methane by soils: A review. Eur. J. Soil Biol., 37, 25-50.

Kell DB (2012) Large-scale sequestration of atmos-pheric carbon via plant roots in natural and agricultural ecosystems: why and how. Philos. Trans. R. Soc. B. 367:1589–1597.

Khosa MK, Sidhu BS, Benbi DK. 2010. Effect of organic materials and rice cultivars on methane emission from rice field. J Environ Biol. 31:281–285.

Koga, N. and Tajima, R. 2011. Assessing energy efficiencies and greenhouse gas emissions under bioethanol-oriented paddy rice production in northern Japan. Journal of Environmental Management, 92(3), 967-973.

Kongchum, M. 2005. Effect of Plant Residue and Water Management Practices on Soil Redox Chemistry, Methane Emission, and Rice Productivity. A Dissertation, he Graduate Faculty of the Louisiana State University and Agricultural and Mechanical College.

Kumar, J.I.N. and Viyol, S. 2009. Short term diurnal and temporal measurement of methane emission in relation to organic carbon, phosphate and sulphate content of two rice fields of central Gujarat, India. Journal of Environmental Biology, 30(2), 241-246.

Kurosawa, M., Yamashiki, Y., Tezuka, T., 2007. Estimation of energy consumption and environmental impact reduction by introducing sustainable agriculture to rice paddy field cultivation. J. Life Cycle Ass. Japan 3 (4), 232–238.

Le Mer, J., Roger, P., 2001. Production, oxidation, emission and consumption of methane by soils: a review. Eur. J. Soil Biol. 37, 25–50.

Liou, S.M., Lo, S.L., Wang, S.H. 2003. A generalized water quality index for Taiwan. Environmental Monitoring and Assessment, 96, 35-52.

Liu, L.; Chen, T.; Wang, Z.; Zhang, H.; Yang, J.; Zhang, J. Combination of site-specific nitrogen management and alternate wetting and drying irrigation increases grain yield and nitrogen and water use efficiency in super rice. Field Crops Res. 2013, 154, 226–235.

Liu, S., Zhang, Y., Lin, F., Zhang, L., Zou, J. 2013. Methane and nitrous oxide emissions from direct-seeded and seedling-transplanted rice paddies in southeast China. Plant Soil, http://dx.doi.org/10.1007/s11104-013-1878-7.

Ma KE, Qiu Q, Lu Y (2010) Microbial mechanism for rice variety control on methane emission from rice field soil. Global Change Biol. doi:10.1111/j13652486200902145

Ma, J., Ji, Y., Zhang, G., Xu, H., Yagi, K. 2013. Timing of midseason aeration to reduce CH4 and N2O emissions from double rice cultivation in China. Soil Sci. Plant Nutr. 59, 35-45.

Ma, Y., Wang, J., Zhou, W., Yan, X., Xiong, Z. 2012. Greenhouse gas emissions during the seedling stage of rice agriculture as affected by cultivar type and crop density. Biol. Fertil. Soils 48, 589-595.

Mariko S, Harazono Y, Owa N, Nouchi I. 1991. Methane in flooded soil water and the emission through rice plants to the atmosphere. Environ Exp Bot. 31:343–350.

Meijide, A., G. Manca, I. Goded, V. Magliulo, P. di Tommasi, G. Seufert, and A. Cescatti. 2011. Seasonal trends and environmental controls of methane emissions in a rice paddy field in Northern Italy. Biogeosciences, 8, 3809-3821.

Muller-Stover, D., Ahrenfeldt, J., Holm, J.K. 2012. Soil application of ash produced by low-temperature fluidized bed gasification: effects on soil nutrient dynamics and crop response. Nutr. Cycl. Agroecosyst. 94, 193-207.

Myanmar Agriculture Sector in Brief. 2019. Crop Sub-Sector. Department of planning, the Ministry of agriculture, livestock and irrigation, Nay Pyi Taw, Myanmar.

Oo, A. Z., K. T. Win, T. Motobayashi and Sonoko Dorothea Bellingrath-Kimura. 2016. Effect of cattle manure amendment and rice cultivars on methane emission from paddy rice soil under continuously flooded conditions. J. Environmental Biology, 37 (5): 1029-1036.

Oo, A.N., Banterng, P., Polthanee, A. and Trelo-Ges, V. 2010. The Effect of Different Fertilizers Management Strategies on Growth and Yield of Upland Black Glutinous Rice and Soil Property. *Asian Journal of Plant Sciences, 9: 414-422.*

Oo, A.Z., L. Nguyen, K.T. Win, G. Cadisch and S.D. Bellingrath-Kimura. 2013. Toposequential variation in methane emissions from double-cropping paddy rice in Northwest Vietnam. Geoderma, 209-210, 41-49.

Oo, A.Z., Sudo, S., Inubushi, K., Chellappan, U., Yamamoto, A., Ono, K., Mano, M., Hayashida, S., Koothan, K., Osawa, T., Terao, Y., Palanisamy, J., Palanisamy, E., Ravi, V. 2018. Mitigation Potential and Yield-Scaled Global Warming Potential of Early-Season Drainage from a Rice Paddy in Tamil Nadu, India. *Agronomy* 2018, *8*, 202; doi:10.3390/agronomy8100202

Oo, A.Z., Sudo, S., Inubushi, K., Mano, M., Yamamoto, A., Ono, K., Osawa, T., Hayashida, S., Patra, P.K., Terao, Y., Elayakumar, P., Vanitha, K., Umamageswari, C., Jothimani, P., Ravi, V. 2018. Methane and nitrous oxide emission from conventional and modified rice cultivation systems in South India. Agriculture, Ecosystems and Environment 252, 148-158.

Oo, A.Z., Win, K.T., Bellingrath-Kimura, S.D., 2015. Within field spatial variation in methane emissions from lowland rice in Myanmar. Springer Plus. 4, 145. http://dx.doi.org/10.1186/s40064-015-0901-2.

Pittelkow, C.M., Adviento-Borbe, M.A., Hill, J.E., Six, J., van Kassel, C., Linquist, B., 2013. Yield-scaled global warming potential of annual nitrous oxide and methane emissions from continuously flooded rice in response to nitrogen input. Agric. Ecosyst. Environ. 177, 10–20.

Qin XB, Li YE, Wan YF, Liao YL, Fan MR, Gao QZ, Liu S, Ma X. 2014. Effect of tillage and rice residue return on CH4 and N2O emission from double rice field. Trans Chin Soc Agric Eng. 30:216–224 (in Chinese with English abstract).

Qin, Y., Liu, S., Guo, Y., Liu, Q. and Zou, J. 2010. Methane and nitrous oxide emissions from organic and conventional rice cropping systems in Southeast China. Biology and Fertility of Soils, 46, 825-834.

Sander, B.O., Wassmann, R., Siopongco, J.D.L.C., 2015. Mitigating greenhouse gas emissions from rice production through water-saving techniques: potential, adoption and empirical evidence. In: Hoanh, C., Smakhtin, V., Johnston, R. (Eds.), Climate Change and Agricultural Water Management in Developing Countries. CAB International, Oxfordshire, UK, pp. 193–207.

Satyanto P. Makarim A K, Fagi A M, Wassmann R & Buendia LV. Crop management affecting methane emissions from irrigated and rainfed rice in Central Jawa (Indonesia), Nutr Cycling Agroecosys, 58 (2000) 85-93.

Setyanto, P., A.B. Rosenani, R. Boer, C.I. Fauziah and M.J. Khanif: The effect of rice cultivars on methane emission from irrigated rice field. Indonesian J Agric Sci., 5, 20-31 (2004).

Shan, J. and Yan, X. 2013. Effects of crop residue returning on nitrous oxide emissions in agricultural soils. Atmospheric Environment, 71, 170-175.

Singh S, Kumar S, Jain MC. 1997. Methane emission from two Indian soils planted with different rice cultivars. Biol Fert Soils. 25:285–289.

Smith, P., Martino, D., Cai, Z., Gwary, D., Janzen, H., Kumar, P., McCarl, B., Ogle, S., O'Mara, F., Rice, C., Scholes, B., Sirotenko, O., 2007. Agriculture. In: Metz, B., Davidson, O.R., Bosch, P.R., Dave, R., Meyer, L.A. (Eds.), Climate Change 2007: Mitigation. Contribution of Working Group III to the Fourth Assessment Report of the Intergovernmental Panel on Climate Change. Cambridge University Press, Cambridge, UK and New York, NY, USA, pp. 497–540.

Tan, X., Shao, D., Liu, H., Yang, F., Xaio, C., Yang, H. Effects of alternate wetting and drying irrigation on percolation and nitrogen leaching in paddy fields. *Paddy Water Env*. 2013, *11*, *1–15*.

Tarlera, S., Capurro, M.C., Irisarri, P., Scavino, A.F., Cantou, G., Roel, A., 2016. Yieldscaled global warming potential of two irrigation management systems in a highly productive rice system. Sci. Agric. 2, 43–50.

Tilman, D., Balzer, C., Hill, J., Befort, B.L., 2011. Global food demand and the sustainable intensification of agriculture. Proc. Nat. Acad. Sci. U. S. A. 108, 20260–20264.

Wang B, Neue HU, Samonte HP. 1997. Effect of cultivar difference ('IR72', 'IR65598' and 'Dular') on methane emission. Agric Ecosyst Environ. 62:31–40.

Wang Z Y, Xu Y C, Li Z, Guo Y X, Wassmann R. Neue N LANTIN R S, Buendia L V, Ding Y P & Wang Z Z. 2000. A four year record of methane emission from irrigated rice fields in Beijing region of China, Nutr Cycling Agroecosys, 58, 55-63.

Wang, J.Y., Jia, J.X., Xiong, Z.Q., Khalil, M.A.K., Xing, G.X., 2011. Water regime-nitrogen fertilizer-straw incorporation interaction: field study on nitrous oxide emissions from a rice agroecosystem in Nanjing. China. Agric. Ecosyst. Environ. 141, 437–446.

Wang, W., Lai, D.Y.F., Wang, C., Tong, C., Zeng, C. 2016. Effects of inorganic amendments, rice cultivars and cultivation methods on greenhouse gas emissions and rice productivity in a subtropical paddy field. Ecological Engineering 95, 770-778.

Win, E.P., Win, K.K., Bellingrath-Kimura, S.D., Oo, A.Z. 2020. Greenhouse gas emissions, grain yield and water productivity: a paddy rice field case study based in Myanmar. Greenhouse Gases: Science and Technology, 0: 1-14.

Xu YC, Wang ZY, Li Z, Wang BJ. 1999. Effect of rice cultivars on methame emission from Beijing rice field. Plant Nutr Fert Sci. 5:93–96 (in Chinese with English abstract).

Yan, X., Akiyama, H., Yagi, K., Akimoto, H., 2009. Global estimations of the inventory and mitigation potential of methane emissions from rice cultivation conducted using the 2006 Intergovernmental Panel on Climate Change Guidelines. Global Biogeochem. Cyc. 23, GB2002.

Yan, X., Yagi, K., Akiyama, H., Akimoto, H., 2005. Statistical analysis of the major variables controlling methane emission from rice fields. Global Change Biol. 11 (7), 1131–1141.

Yang, J.-C.; Zhang, J.-H. Grain-filling problem in ‘super’ rice. J. Exp. Bot. 2010, 61, 1–5.

Yang, X., Shang, Q., Wu, P., Liu, J., Shen, Q., Guo, S., Xiong, Z., 2010. Methane emissions from double rice agriculture under long-term fertilizing systems in Hunan, China. Agric. Ecosyst. Environ. 137, 308–316.

Ye, M.; Song, Y.; Long, J.; Wang, R.; Baerson, S.R.; Pan, Z.; Zhu-Salzman, K.; Xie, J.; Cai, K.; Luo, S.; et al. Priming of jasmonate-mediated antiherbivore defense responses in rice by silicon. Proc. Natl. Acad. Sci. USA 2013, 110, E3631–E3639.

Yu J, Wang LL, Yan XJ, Tian YL, Deng AX, Zhang WJ. 2013. Super rice cropping will enhance rice yield and reduce CH4 emission: a case study in Nanjing. Chin Rice Sci. 20:427–433.

Zhang, H.; Xue, Y.;Wang, Z.; Yang, J.; Zhang, J. Alternate wetting and moderate soil drying improves root and shoot growth in rice. Crop Sci. 2009, 49, 2246–2260.

Zheng, H., Huang, H., Yao, L., Liu, J., He, H., Tang, J. 2014. Impacts of rice varieties and management on yield-scaled greenhouse gas emissions from rice fields in China: A meta-analysis. Biogeosciences, 11, 3685-3693.

Zou, J., Huang, Y., Qin, Y., Liu, S., Shen, Q., Pan, G., Lu, Y., Liu, Q., 2009. Changes in fertilizer-induced direct N2O emissions from paddy fields during ricegrowing season in China between 1950 and 1990. Global Change Biol. 15, 229–242.

Xiong, Z., Yinglie, L., Wu, Z., Huang, T. Differences in net global warming potential and greenhouse gas intensity between major rice-based cropping systems in China. *Scientific Report* **5**, 17774 (2015).

Pittelkow, C. M., Adviento-Borbe, M. A., Kessel, C., Hill, J. E. & Linquist, B. A. Optimizing rice yields while minimizing yield-scaled global warming potential. *Global Change Biol.* **20**, 1382–1393 (2014).

Van Groenigen, J. W., Velthof, G. L., Oenema, O., Van Groenigen, K. J. & Van Kessel, C. Towards an agronomic assessment of N2O emissions: a case study for arable crops. *Eur. J. Soil Sci.* 61, 903–913 (2010).

Wu, X., Wang, W., Xie, X., Yin, C., Hou, H., Yan, W., Wang, G. 2018. Net global warming potential and greenhouse gas intensity as affected by different water management strategies in Chinese double rice-cropping systems. Scientific reports, 8: 779.

Click here to access/download
Supporting Information
NZSGJQ41_200929_GHG_text_final.docx
