# Supplementary material for: Influence of rice varieties, organic manure and water management on greenhouse gas emissions from paddy rice soils
Source: PLoS One. 2021 Jun 30;16(6):e0253755. doi: 10.1371/journal.pone.0253755 (PMC8244889; doi:10.1371/journal.pone.0253755)
Supplement: Attachment — Submitted filename: PONE-D-20-31281_R1-comments.pdf [file pone.0253755.s006.pdf]

PLOS ONE

Influence of rice varieties, organic manure and water management on greenhouse gas emissions from paddy rice soils

--Manuscript Draft--

**Manuscript Number:**	PONE-D-20-31281R1
**Article Type:**	Research Article
**Full Title:**	Influence of rice varieties, organic manure and water management on greenhouse gas emissions from paddy rice soils
**Short Title:**	Rice varieties, organic manure and water management on greenhouse gas emissions
**Corresponding Author:**	Ei Phyu Win, Ph.D.<br>Yezin Agricultural University<br>Nay Pyi Taw, Nay Pyi Taw MYANMAR
**Keywords:**	mitigation; Greenhouse Gas Emissions; rice varieties; organic fertilizer; Water Management
**Abstract:**	The study is focused on impact of manure application, rice varieties and water management on greenhouse gas (GHG) emissions from paddy rice soil in pot experiment. The objectives of this study were a) to assess the effect of different types of manure amendments and rice varieties on greenhouse gas emissions and b) to determine the optimum manure application rate to increase rice yield while mitigating GHG emissions under alternate wetting and drying irrigation in paddy rice production. The first pot experiment was conducted at the Department of Agronomy, Yezin Agricultural University, Myanmar, in the wet season from June to October 2016. Two different organic manures (compost and cow dung) and control (no manure), and two rice varieties; Manawthukha (135 days) and IR-50 (115 days), were tested. The results showed that cumulative CH_4 emission from Manawthukha (1.084 g CH_4 kg^{-1} soil) was significantly higher than that from IR-50 (0.683 g CH_4 kg^{-1} soil) ($P<0.0046$) with yield increase ($P<0.0164$) because of the longer growth duration of the former. In contrast, higher cumulative nitrous oxide emissions were found for IR-50 (2.644 mg N_2O kg^{-1} soil) than for Manawthukha (2.585 mg N_2O kg^{-1} soil). However, IR-50 showed less global warming potential (GWP) than Manawthukha ($P<0.0050$). Although not significant, the numerically lowest CH_4 and N_2O emissions were observed in the cow dung manure treatment (0.808 g CH_4 kg^{-1} soil, 2.135 mg N_2O kg^{-1} soil) compared to those of the control and compost. To determine the effect of water management and organic manures on greenhouse gas emissions, second pot experiments were conducted in Madaya township during the dry and wet seasons from February to October 2017. Two water management practices {continuous flooding (CF) and alternate wetting and drying (AWD)} and four cow dung manure rates {(1) 0 (2) 2.5 t ha^{-1} (3) 5 t ha^{-1} (4) 7.5 t ha^{-1} } were tested. The different cow dung manure rates did not significantly affect grain yield or greenhouse gas emissions in this experiment. Across the manure treatments, AWD irrigation significantly reduced CH_4 emissions by 70% during the dry season and 66% during the wet season. Although a relative increase in N_2O emissions under AWD was observed in both rice seasons, the global warming potential was significantly reduced in AWD compared to CF in both seasons ($P<0.0002$, $P<0.0000$) according to reduced emission in CH_4. Therefore, AWD is the effective mitigation practice for reducing GWP without compromising rice yield while manure amendment had no significant effect on GHG emission from paddy rice field. Besides, AWD saved water about 10% in dry season and 19% in wet season.
**Order of Authors:**	Ei Phyu Win, Ph.D.
	Kyaw Kyaw Win
	Sonoko D. Bellingrath-Kimura
	Aung Zaw Oo
**Response to Reviewers:**	Dear Sir,<br>I really thank you very much for your valuable and constructive comments on this manuscript. I have revised all reviewers' comments and may I know if you have more

Powered by Editorial Manager® and ProduXion Manager® from Aries Systems Corporation

comments and suggestions to be manuscript improved.
Reviewer #1: Some statistical analysis information is missing especially in the tables but this can be corrected if the authors have data. Also more information on the analysis methods have been requested in the attached reviewer notes.
Answer: I have revised them.

Reviewer #2: Comments to the author and editors
The study is focused on impact of manure application, rice verities and water management on GHG emission from paddy soil putted in pot experiment. The objectives of this study were a) to assess the effect of different types of manure amendments and rice varieties on greenhouse gas emissions and b) to determine the optimum manure application rate to increase rice yield while mitigating GHG emissions under alternate wetting and drying irrigation in paddy rice production. I appreciate the effort of the worker for reporting data, however, the study has been conducted in the pot and data has reported for only one season which is not adequate to draw sound conclusion. It would have been more appropriate if at least two year data might have been generated. There are many research works already reported on this aspect with long duration of experiment from field trial. The manuscript also lack proper synthesis and sound discussion. It may be suitable for publication in local journal of the study area. The some comments has been made in the pdf of the manuscript and some broad comments are enlisted below
1. The readability of the manuscript is poor
2. The abstract lack genesis, objective and conclusion of the research
3. Material and methods lack: information on feed-stock of compost, clear statement regarding control and N-fertilization, intercultural operation, harvesting date, dimension of gas chamber and duration of gas sampling. Cumulative CH4 emission is highly dependent on duration of gas sampling and biomass yield especially number of tiller. No data has been presented on vegetative growth of the two rice varieties.
4. The clear difference in compost and cow dung manure has not been stated: the method of preparation, feed-stock used etc.
5. Table 2.1 and 2.2 should be similar in terms of parameters reported
6. The result section and discussion need re-synthesis to draw final conclusion. Control has shown lowest GHG intensity in first experiment, but Cow dung application has been recommended. Need proper discussion
7. In second experiment, increasing rate of cow dung has reduced CH4 emission as compared to control (control> C 2.5 t/ha > C 5 t/ha < c 7.5 t/ha) up to 5 t/ha then it increased. Need detailed discussion on this trend
Answer: Sir, thank you very much for your constructive comments and valuable usage of words. I had revised the abstract with your comments and suggestions and I substituted your comments to our manuscript to be improved of readability of it. I have revised all comments. Please may I know more comments and suggestions to be improved of our manuscript and I really thank you and appreciate your valuable comments.
Reviewer #3: The statistical design used in the not suitable for this study. The authors have to use CRD design for analysing their data. Split plot design used by authors is not for pot experiments. This type of design is used for field experiments. This is why I feed the the manuscript is not technically sound.
I suggest the authors to revise the results based on revised statistical analysis and modified reporting of pot experiment data as pot experiment result are not reported on per hectare basis
Answer: Sir, I revised the manuscript with your suggestion. I revised the statistical analysis to two factors with CRD design. Thank you very much for your constructive and valuable comments. Please may I know more comments and suggestions and I really appreciate your fruitful comments.

Additional Information:	
**Question**	**Response**
**Financial Disclosure**<br><br>Enter a financial disclosure statement that describes the sources of funding for the work included in this submission. Review	Ei Phyu Win<br>one grant<br>Cuomo Foundation<br>https://www.cuomo.foundation/<br>No

Powered by Editorial Manager® and ProduXion Manager® from Aries Systems Corporation

the submission guidelines for detailed requirements. View published research articles from *PLOS ONE* for specific examples.<br><br>This statement is required for submission and **will appear in the published article** if the submission is accepted. Please make sure it is accurate.<br><br>**Unfunded studies**<br>Enter: *The author(s) received no specific funding for this work.*<br><br>**Funded studies**<br>Enter a statement with the following details:<br>• Initials of the authors who received each award<br>• Grant numbers awarded to each author<br>• The full name of each funder<br>• URL of each funder website<br>• Did the sponsors or funders play any role in the study design, data collection and analysis, decision to publish, or preparation of the manuscript?<br>• **NO** - Include this sentence at the end of your statement: *The funders had no role in study design, data collection and analysis, decision to publish, or preparation of the manuscript.*<br>• **YES** - Specify the role(s) played.<br><br>* typeset	
**Competing Interests**<br><br>Use the instructions below to enter a competing interest statement for this submission. On behalf of all authors, disclose any competing interests that could be perceived to bias this work—acknowledging all financial support and any other relevant financial or non-financial competing interests.<br><br>This statement **will appear in the published article** if the submission is accepted. Please make sure it is accurate. View published research articles from *PLOS ONE* for specific examples.	The authors have declared that no competing interests exit.

**NO authors have competing interests**<br><br>Enter: *The authors have declared that no competing interests exist.*<br><br>**Authors with competing interests**<br><br>Enter competing interest details beginning with this statement:<br><br>*I have read the journal's policy and the authors of this manuscript have the following competing interests: [insert competing interests here]*<br><br>* typeset	
**Ethics Statement**<br><br>Enter an ethics statement for this submission. This statement is required if the study involved:<br><br>• Human participants<br>• Human specimens or tissue<br>• Vertebrate animals or cephalopods<br>• Vertebrate embryos or tissues<br>• Field research<br><br>Write "N/A" if the submission does not require an ethics statement.<br><br>General guidance is provided below. Consult the submission guidelines for detailed instructions. **Make sure that all information entered here is included in the Methods section of the manuscript.**	N/A

Powered by Editorial Manager® and ProduXion Manager® from Aries Systems Corporation

**Format for specific study types**<br><br>**Human Subject Research (involving human participants and/or tissue)**<br>• Give the name of the institutional review board or ethics committee that approved the study<br>• Include the approval number and/or a statement indicating approval of this research<br>• Indicate the form of consent obtained (written/oral) or the reason that consent was not obtained (e.g. the data were analyzed anonymously)<br><br>**Animal Research (involving vertebrate animals, embryos or tissues)**<br>• Provide the name of the Institutional Animal Care and Use Committee (IACUC) or other relevant ethics board that reviewed the study protocol, and indicate whether they approved this research or granted a formal waiver of ethical approval<br>• Include an approval number if one was obtained<br>• If the study involved *non-human primates*, add *additional details* about animal welfare and steps taken to ameliorate suffering<br>• If anesthesia, euthanasia, or any kind of animal sacrifice is part of the study, include briefly which substances and/or methods were applied<br><br>**Field Research**<br><br>Include the following details if this study involves the collection of plant, animal, or other materials from a natural setting:<br>• Field permit number<br>• Name of the institution or relevant body that granted permission	
**Data Availability**<br><br>Authors are required to make all data underlying the findings described fully available, without restriction, and from the time of publication. PLOS allows rare exceptions to address legal and ethical concerns. See the PLOS Data Policy and FAQ for detailed information.	Yes - all data are fully available without restriction

A Data Availability Statement describing where the data can be found is required at submission. Your answers to this question constitute the Data Availability Statement and **will be published in the article**, if accepted.<br><br>**Important:** Stating 'data available on request from the author' is not sufficient. If your data are only available upon request, select 'No' for the first question and explain your exceptional situation in the text box.<br><br>Do the authors confirm that all data underlying the findings described in their manuscript are fully available without restriction?	
**Describe where the data may be found in full sentences. If you are copying our sample text, replace any instances of XXX with the appropriate details.**<br><br>• If the data are **held or will be held in a public repository**, include URLs, accession numbers or DOIs. If this information will only be available after acceptance, indicate this by ticking the box below. For example: *All XXX files are available from the XXX database (accession number(s) XXX, XXX.*).<br>• If the data are all contained **within the manuscript and/or Supporting Information files**, enter the following: *All relevant data are within the manuscript and its Supporting Information files.*<br>• If neither of these applies but you are able to provide **details of access elsewhere**, with or without limitations, please do so. For example:<br><br>*Data cannot be shared publicly because of [XXX]. Data are available from the XXX Institutional Data Access / Ethics Committee (contact via XXX) for researchers who meet the criteria for access to confidential data.*<br><br>*The data underlying the results presented in the study are available from (include the name of the third party*	All relevant data are withing the manuscript file.

Powered by Editorial Manager® and ProduXion Manager® from Aries Systems Corporation

and contact information or URL).<br>• This text is appropriate if the data are owned by a third party and authors do not have permission to share the data.<br><br>* typeset	
Additional data availability information:	Tick here if your circumstances are not covered by the questions above and you need the journal’s help to make your data available.

Revised Manuscript
Click here to access/download;Manuscript;Revised Manuscript.docx

Influence of rice varieties, organic manure and water management on greenhouse gas emissions from paddy rice soils

Ei Phyu Win[1*], Kyaw Kyaw Win[1], Sonoko D. Bellingrath-Kimura[2], Aung Zaw Oo[3]

[1]Department of Agronomy, Yezin Agricultural University, Myanmar
[2]Institute of Land Use Systems, Faculty of Life Science, Humboldt-Universität zu Berlin, Germany
[3]Japan International Research Center for Agricultural Sciences, Ohwashi, Tsukuba, Ibaraki, Japan

Abstract

The study is focused on impact of manure application, rice varieties and water management on greenhouse gas (GHG) emissions from paddy rice soil in pot experiment. The objectives of this study were a) to assess the effect of different types of manure amendments and rice varieties on greenhouse gas emissions and b) to determine the optimum manure application rate to increase rice yield while mitigating GHG emissions under alternate wetting and drying irrigation in paddy rice production. The first pot experiment was conducted at the Department of Agronomy, Yezin Agricultural University, Myanmar, in the wet season from June to October 2016. Two different organic manures (compost and cow dung) and control (no manure), and two rice varieties; Manawthukha (135 days) and IR-50 (115 days), were tested. The results showed that cumulative CH_4 emission from Manawthukha (1.084 g CH_4 kg^{-1} soil) was significantly higher than that from IR-50 (0.683 g CH_4 kg^{-1} soil) ($P<0.0046$) with yield increase ($P<0.0164$) because of the longer growth duration of the former. In contrast, higher cumulative nitrous oxide emissions were found for IR-50 (2.644 mg N_2O kg^{-1} soil) than for Manawthukha (2.585 mg N_2O kg^{-1} soil). However, IR-50 showed less global warming potential (GWP) than Manawthukha ($P<0.0050$). Although not significant, the numerically lowest CH_4 and N_2O emissions were observed in the cow dung manure treatment (0.808 g CH_4 kg^{-1} soil, 2.135 mg N_2O kg^{-1} soil) compared to those of the control and compost. To determine the effect of water management and organic manures on greenhouse gas emissions, second pot experiments were conducted in Madaya township during the dry and wet seasons from February to October 2017. Two water

management practices {continuous flooding (CF) and alternate wetting and drying (AWD)} and four cow dung manure rates {(1) 0 (2) 2.5 t ha^{-1} (3) 5 t ha^{-1} (4) 7.5 t ha^{-1}} were tested. The different cow dung manure rates did not significantly affect grain yield or greenhouse gas emissions in this experiment. Across the manure treatments, AWD irrigation significantly reduced CH_4 emissions by 70% during the dry season and 66% during the wet season. Although a relative increase in N_2O emissions under AWD was observed in both rice seasons, the global warming potential was significantly reduced in AWD compared to CF in both seasons ($P<0.0002$, $P<0.0000$) according to reduced emission in CH_4. Therefore, AWD is the effective mitigation practice for reducing GWP without compromising rice yield while manure amendment had no significant effect on GHG emission from paddy rice field. Besides, AWD saved water about 10% in dry season and 19% in wet season.

Keywords: mitigation, greenhouse gas emissions, rice varieties, organic fertilizer, water management

*Corresponding author

E-mail: eiphyuwin@yau.edu.mm (EP)

Introduction

Developing new strategies is necessary to achieve the dual goals of ensuring food security and protecting natural resources and the environment through reduced greenhouse gas (GHG) emissions [1, 2]. It is estimated that nitrous oxide (N_2O) and methane (CH_4) emissions may increase by 35-60% and 60%, respectively, by 2030 [3]. Flooded rice soils are an important source of global CH_4 emissions [4, 5], and rice-based cropping systems can emit substantial amounts of N_2O [6] during the rice season itself [7].

Rice is one of the most important cereal grains and staple food crops globally and is particularly important in Asia [8]. Rice paddies contribute to the emission of the two most important GHGs; methane and nitrous oxide. IPCC [9] reported that rice fields contribute about 30% and 11% of global agricultural CH_4 and N_2O emissions, respectively. With a

linearly increasing rate of 0.26% per year during the recent few decades, the atmospheric N_2O concentration has increased by 18% compared to the preindustrial level. Methane and nitrous oxide have long atmospheric lifetimes of 12 and 114 years, respectively, and account for 20% and 7%, respectively, of global radiative forcing [10]. The high global warming potential (GWP) of CH_4 and N_2O, 34 and 298 times that of CO_2 at a 100-year time horizon, makes them major contributors to climate change [11]. In recent years, suitable management practices have been developed for achieving both improvement in rice yields and mitigation of GHG emissions, which include the development of new rice varieties [12], the application of manure such as cow dung [13], the selection of appropriate cultivation methods [14] and the timing of drainage [15].

The magnitude of CH_4 emissions from rice plants is regulated by complex and dynamic interactions among the plants, environment, and microorganisms [16]. Methane produced in flooded rice soils is emitted to the atmosphere by molecular diffusion, ebullition or plant-mediated transport. Approximately 80-90% of the total CH_4 flux is emitted to the atmosphere from the rhizosphere via the rice plant [17]. An increase in plant biomass [18] and tiller number [19] enhanced CH_4 oxidization activity by enlarging the volume of aerenchyma and enhancing O_2 transport from the atmosphere to the rhizosphere. Ma et al. [20] revealed that a hybrid rice variety with 50–60% higher shoot biomass emitted less CH_4 than an indica rice variety, possibly due to higher CH_4 oxidization activity.

Nitrous oxide is produced as a by-product of nitrification, denitrification, nitrifier denitrification, etc. and moisture content is a key factor governing N_2O production in soils [21]. Ciarlo et al. [22] found that denitrification is dominant pathway for N_2O emission when the water-filled pore space in soils is high (80%), and if the soils were saturated than this level, most of N_2O would be reduced to nitrogen.

Selecting a rice variety that has high productivity and low GHG emissions is crucial for improving crop yield and mitigating climate change; however, research examining the effects of rice varieties has mostly focused on CH_4 flux so far [12, 23, 24], with little focus on N_2O flux [25]. Many studies reported that the effect of rice varieties on methane emissions is mostly related to rice growth performance, i.e., the number of plant tillers and above- and belowground biomass, root exudates and root arenchyma [26-30]. Although significant positive relationship have been found between rice biomass and methane fluxes [31, 32], a comparison of rice varieties has produced different results [33, 34].

Organic residue amendments have been practised to improve soil fertility in paddy production. The organic matter in paddy fields originates from both direct by-products of rice production (such as sloughed-off root cells and root exudates) and added materials (manures and previous crop residues). The addition of organic carbon to the soil, whether it comes from the disposal of crop residues or as organic fertilizer, appears to be the most important factor in methane production [32]. Waterlogged conditions are ideal for the decomposition of organic matter in paddy fields. The methane production from rice soil can be increased by addition of cow manure as a source of organic material [35]. Nitrous oxide emissions from applied fertilizer and manures can vary with different environmental factors (e.g., climate and soil conditions), crop factors (e.g., crop type and crop residues), and management practices (e.g., type of manure and fertilizer, application rate, time of application) [36].

Although there are wide range of factors influencing methane emission from paddy soil [37-39], water management and organic amendment are the two main drivers of methane production. Under anaerobic soil conditions, methanogens (methane-producing bacteria) produce methane by oxidation of organic matter during anaerobic respiration. Flooded rice soils are known to have strong denitrification activity emitting some amount of N_2O from rice soils. However, it is also guided by the water management conditions of the rice field. Nitrate

and nitrite in rice soils is limited due to submerged conditions. The oxygen supply due to decomposition of organic matter, roots, and also through vascular transport via tillers may help in production of nitrate in rice soils. Sometimes due to prolonged submergence of the rice fields, the soil nitrate and nitrite N (available due to mineralization of organic matter) is completely reduced to N_2 gas thereby resulting in low N_2O emission. This varies with the prevailing conditions of the rice field. Although minimal N_2O emissions are likely from flooded soils, some off-site (indirect) N_2O emissions are likely from irrigated rice production due to the addition of nitrogen fertilizer to fields [40].

Myanmar ranks the sixth largest production for rice in the world. Rice is the country's most important crop and is grown on 7.3 million ha [41]. The conventional rice production method commonly used by the farmers in Myanmar includes transplanting old seedlings (30-45) under continuous flooding conditions and the intensive use of organic fertilizers such as manure or compost. However, there is very limited information on methane emissions from the flooded rice fields of Myanmar, although more than half of the cultivated area is devoted to rice production [13, 42]. Thus, the objectives of this study were a) to assess the effect of different types of manure amendments and rice varieties on greenhouse gas emissions and b) to determine the optimum manure application rate to increase rice yield while mitigating GHG emissions under alternate wetting and drying irrigation in paddy rice production.

Materials and methods

The first pot experiment was conducted at open field, Department of Agronomy, Yezin Agricultural University (19° 45'N and 96° 6'E), Myanmar, during the wet season (June – October), 2016 to study the local production potential of GHG emission in this area. A two-factor factorial experiment with completely randomized design was used with 3 replications. The factor A was assigned into two categories of organic manure (compost and cow dung) and control (no manure). The compost is collected from straw compost making process and

stored for ten months. The cow dung is resulted from farmer traditional heap method for ten months. The amount of organic manure for cow dung and compost treatments was based on the nitrogen content of the organic manure analysis. The recommended chemical fertilizer amounts are 60 kg N ha^{-1}, 30 kg P_2O_5 ha^{-1}, and 20 kg K_2O ha^{-1} for all treatments. The recommended amounts of nitrogen fertilizer of compost and cow dung treatments were replaced by compost and cow dung manures which was based on same amount of carbon content. Therefore, 3 t ha^{-1} of compost was applied to compost treatment, and 3.15 ton cow dung + chemical fertilizer (22.15 kg N ha^{-1}) was applied to cow dung treatment to get the recommended amount of nitrogen fertilizer based on their nitrogen analysis. The control treatment received only recommended amount of chemical nitrogen fertilizer. The factor B was two types of rice varieties: Manawthukha (135 days) and IR-50 (115 days). The two rice varieties were grown in a concrete pot (52.5 cm in diameter and 45 cm in height). The soil was collected from a lowland irrigated rice field. Twenty-one-day-old seedlings were transplanted with two seedlings per pot. Compost and cow dung manures were broadcasted at 14 days before transplanting to avoid transplanting shock due to the manure decomposition process. The soil was analysed for pH (1:5 soil: water suspension), electrical conductivity (1:5 soil: water suspension), total N% (Kjaldehl distillation method), organic matter% (Tyurin's method), calcium chloride extractable SO_4-S (Turbidity method) and Texture (pipette method). The manures were analysed for total N% (Kjaldehl distillation method), total P% (Molybivanado phosphoric acid method), total K% (Wet digestion with HNO3: HCLO4 (4:1), total S% (Turbidity method) and organic carbon % (Tyurin's method). Table 1 shows the physiochemical properties of the soil and manures. The recommended amount of T-super (30 kg P_2O_5 ha^{-1}) was applied as basal fertilizer, and the recommended amount of potash (20 kg K_2O ha^{-1}) was applied with two split applications (as basal fertilizer and at the panicle initiation stage) to all treated pots. The water level was maintained at 5 cm throughout

the rice growing period except during the drying period before harvest. During the rice growing season, weather data were recorded at the Department of Agronomy and are shown in Fig 1. The average minimum and maximum temperatures during the rice growing season (wet season) were 23.9 and 31.8°C, respectively, with 739 mm of rainfall.

The second pot experiment was conducted in a farmer's field, Madaya Township (22° 13' 0'' N and 96° 7' 0'' E), Myanmar, during the dry and wet seasons (February – October 2017) to assess the effect of cow dung manure and water management on greenhouse gases emissions from paddy rice soils. The pots were arranged in a two-factor factorial experiment with completely randomized design with three replications. Water management (continuous flooding (CF) and alternate wetting and drying (AWD) was arranged as factor A. Different rates of organic manure were assigned as factor B. In this study, cow dung manure was applied as an organic source based on the reduced global warming potential (GWP) value in previous study and widely used in the study area. The cow dung manure treatments (OM_0 = no cow dung, OM_1 = half of the recommended cow dung (2.5 t ha^{-1}), OM_2 = the recommended rate of cow dung (5 t ha^{-1}) and OM_3 = one and a half times the recommended rate of cow dung (7.5 t ha^{-1}), were applied seven days before transplanting. The recommended rate of cow dung manure is 5 t ha^{-1}. Each pot received the recommended fertilizer at the rates of 90 kg N ha^{-1}, 30 kg P_2O_5 ha^{-1}, and 20 kg K_2O ha^{-1}. Urea, T-super and potash were used as nutrient sources. Urea was applied as three equal split applications at the active tillering, panicle initiation and heading growth stages. T-super was applied only as a basal fertilizer at one day before transplanting, and potash fertilizer was applied in two equal split applications as a basal fertilizer and at panicle initiation. The soil was collected from a lowland irrigated rice field and analysed for pH (1:5 soil: water suspension), available N (Alkaline permanganate method), available P (9C-Olsen’s P-Malachite green), available K (1N Ammonium acetate extraction), total N% (Kjaldehl distillation method), organic matter%

(Tyurin's method), cation exchange capacity (CEC) (Leaching method) and texture (Pipette method). The cow dung manure was collected from farmer traditional heap method stored for ten months, and analysed for total N% (Kjaldehl distillation method) and organic carbon % (Tyurin's method). Table 2 shows the physiochemical properties of the soil and cow dung.

IR-50 rice variety (115 days) was used based on reduced emission in previous study and widely grown in the study area. Dry-season rice was transplanted on 1st February 2017 and harvested on 14 May 2017. Wet-season rice was transplanted on 8 July 2017 and harvested on 11st October 2017. Just after transplanting, a base (40 cm in diameter with 2.5 cm water seal, 5 cm in height) was placed around the plants used for gas sampling to avoid disturbing the environmental conditions around the rice plants during chamber deployment in both experiments. The base was equipped with a water seal to ensure a gas-tight closure. The base remained embedded in the soil throughout the rice growing period. Water tubes (PVC pipe-25 cm height with six row holes each 2.5 cm apart) were installed in the AWD pots at a depth of 15 cm below the soil surface between the seedlings and the base just after transplanting. For AWD pots, whenever there was no water in the water tube, irrigation water was applied to a 5 cm depth above the soil surface. The irrigation interval ranged from 4 to 9 days, and the amount ranged from 7 to 13 litres depending on the different rates of cow dung manure in the AWD pots. Withdrawal of water was started one week before the harvest period in all irrigated pots. During the rice growing seasons, weather data were collected from the Department of Agricultural Research, Madaya and are shown in Fig 2. The average minimum and maximum temperatures were 21.8°C and 35.6°C during the dry season and 26.8°C and 35.7°C during the wet season, respectively. The total rainfall amounts were 201.7 mm during the dry season and 420.6 mm during the wet season.

Table 1. Physiochemical properties of experimental soil and organic manures used in the first pot experiment

Item	Total N%	Total P%	Total K%	O.C%				
Cow dung	1.2	1.0	2.1	23.3				
Compost	2.0	2.9	1.9	24.5				
**Item**	**pH**	**EC (dS/m)**	**Total N%**	**OM%**	**SO4-S (mg/kg)**	**Texture %**		
						**Sand**	**Silt**	**Clay**
Soil	6.7	0.4	0.2	2.7	12	79.74	13.28	6.98

Table 2. Physiochemical properties of experimental soil and cow dung manure used in the second pot experiment

Analytical Item	Unit	Analytical Result	
Soil pH		7.4	Moderately alkaline
Available N	mg kg^{-1}	50	Low
Available P	mg kg^{-1}	13	Medium
Available K	mg kg^{-1}	78	Low
Total N	%	0.17	
Organic matter	%	1.8	Low
CEC	$cmol_c$ kg^{-1}	11	Low
Sand	%	87	
Silt	%	4	
Clay	%	9	
Textural class			Loamy sand
**Cow dung manure**		**Dry season**	**Wet season**
Total N	%	1.32	1.2
Organic carbon	%	16	23.3

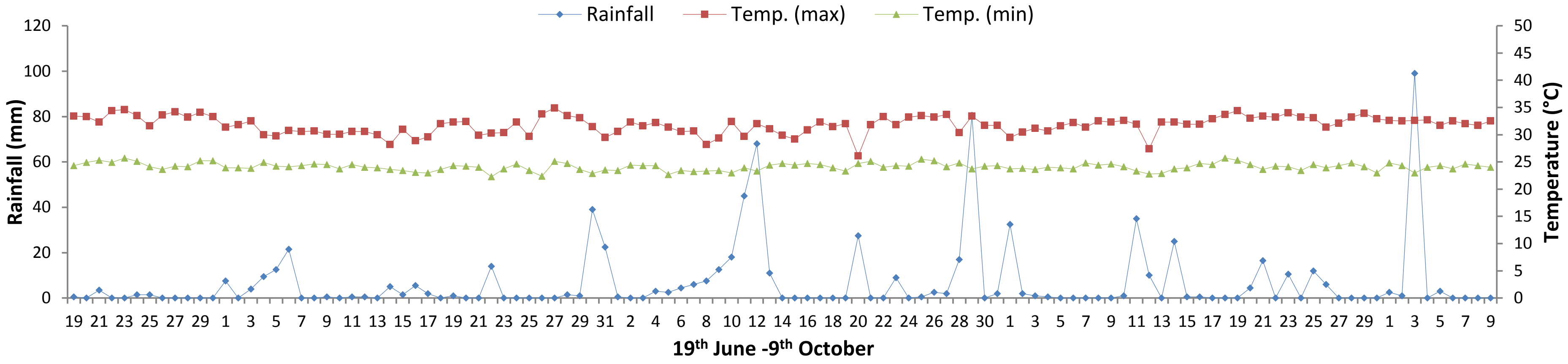

Fig 1. Daily rainfall, maximum and minimum temperatures in Yezin Agricultural University, Myanmar during wet season, 2016

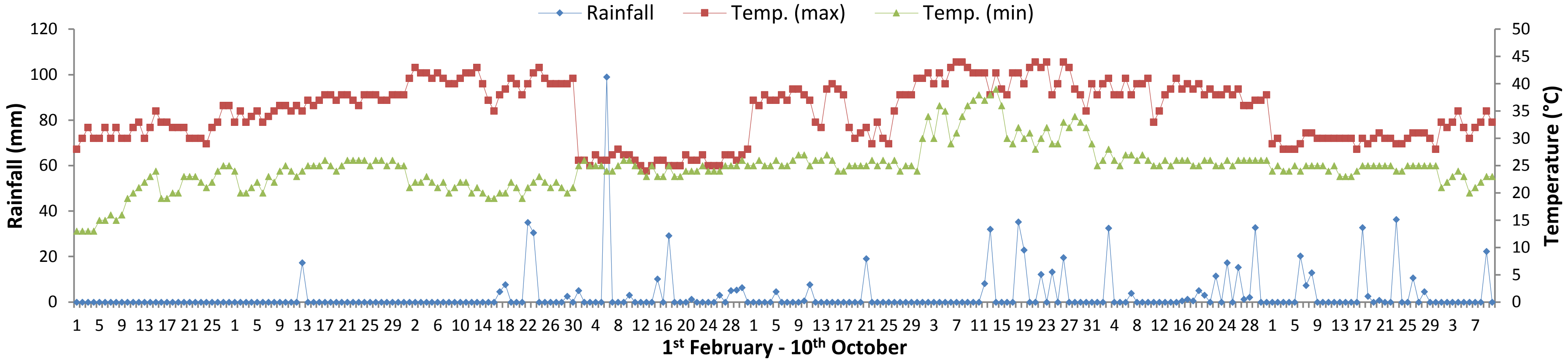

Fig 2. Daily rainfall, maximum and minimum temperatures in Madaya township, Myanmar during dry and wet seasons, 2017

Gas sample collection, analysis and calculation

A two-bonded chamber with a total capacity of 77 L (93 cm height) was used for collecting the gas sample. To thoroughly mix the gases in the chamber, the chamber was equipped with a small 12 volt DC fan connected with three 9-volt dry battery [43]. For CH_4 calculation, the temperature was recorded with a digital thermometer (TT-508 Tanita, Tokyo, Japan). To compensate for the air pressure changes between the increased temperature and gas sampling, an air buffer bag (1-L Tedlar bag) was attached to the chamber. The silicon rubber tube connected with three-way stop cock was inserted airtight into a hole on the chamber. The gas sample was taken with an airtight 50 ml syringe by connecting it to the three-way stop cock and then transferred to a 20 ml pre-evacuated glass vial.

Gas sampling was performed at 7-day intervals starting from 1 day after transplanting until harvest by the closed chamber method in pot experiment 1. In pot experiment 2, the first two gas samplings were performed at one-week intervals after transplanting, and later gas samplings were performed at 10-day intervals. The gas samples were collected from 9:00 am until 12:00 am and three times (0, 15, 30 min) for each treatment for gas flux calculation.

Methane and N_2O concentrations were analysed with a gas chromatograph (GC 2014, Shimadzu Corporation, Kyoto, Japan) equipped with a flame ionization detector (FID) and an electron capture detector (ECD). The amount of CH_4 and N_2O fluxes was calculated by using the following equation:

$$Q = (V/A) \text{ x } (\Delta c/\Delta t) \text{ x } (M/22.4) \text{ x } (273/K)$$

where Q = the flux of gas (mg m^{-2} min^{-1})

V = the volume of the chamber (m^3)

A = the base area of the chamber (m^2)

$(\Delta c/\Delta t)$ = the rate of increase or decrease in the gas concentration (mg m^{-3}) per unit time (min)

M = the molar weight of the gas

K = Kelvin temperature of the air temperature inside the chamber

Total emissions were calculated by interpolation method of sample gas analysis at each gas measurement for the growing period. In this study, the IPCC factors were used to calculate the combined GWPs for 100 years (GWP= (25×CH_4) + (298×N_2O)) in kg CO_2-equivalents ha^{-1} [9] for CH_4 and N_2O. The yield was recorded from each pot. The grains were threshed, cleaned and sun-dried. Yields were adjusted at 14% moisture by using the following formula to remove the error due to the different moisture content, and grain moistures were measured by using a grain moisture metre (model: GMK-303RS).

Adjusted grain weight at 14% moisture level = A x W

where A = Adjustment coefficient

W = Weight of harvested grains

$$A= \frac{100\text{-moisture}\%}{86}$$

In both water management practices, the water was applied with 1.2 L water cup. The total amount of water applied throughout the growing season was recorded and water saving in AWD was calculated.

The greenhouse gas intensity (GHGI) was calculated by dividing the GWP by the rice grain yield [44-46].

GHGI = GWP/grain yield (kg CO_2-eq. kg^{-1} grain)

Statistical analysis

The data were analysed by using Statistix (version 8.0). Mean comparisons were performed by least significant difference (LSD) test at the 5% level.

Results

First pot experiment during the wet season, 2016

Methane emission: During the early growth stage, low CH_4 emission was observed for both varieties until 36 days after transplanting (DAT), and then CH_4 emission flux gradually increased with some fluctuations until harvest (Fig 3). The mean cumulative CH_4 emissions from the control, compost, and cow dung treatments were 0.893, 0.951 and 0.808 g CH_4 kg^{-1} soil, respectively. Although the effect was not significant, cow dung amendment reduced cumulative CH_4 emissions by 9.5% compared with the control and 15% compared to compost. When comparing the two rice varieties, the total cumulative CH_4 emissions were significantly higher in the Manawthukha variety (1.084 g CH_4 kg^{-1} soil) than in the IR-50 variety (0.683 g CH_4 kg^{-1} soil) ($P<0.0046$) (Table 3). The IR-50 variety reduced cumulative CH_4 emissions by 37% compared to the Manawthukha variety. During the rice growing season, cumulative CH_4 emissions were higher in later growth stages (reproductive and ripening) than in the vegetative growth stage.

Nitrous oxide emission: High nitrous oxide emission was observed during very early growth until 15 DAT in both varieties. After that, a small amount of N_2O emission was found until harvest (Fig 4). Although there was no significant difference in N_2O emissions among the manure treatments, higher emission (3.218 mg N_2O kg^{-1} soil) was recorded from the control (no manure) compared to the compost (2.491 mg N_2O kg^{-1} soil) and cow dung (2.135 mg N_2O kg^{-1} soil) (Table 1). Cow dung manure reduced cumulative N_2O emissions by 33.7% compared with the control and 14.3% compared to compost. There was also no significant difference in cumulative N_2O emissions among the tested varieties: Manawthukha variety (2.585 mg N_2O kg^{-1} soil), IR-50 (2.644 mg N_2O kg^{-1} soil) (Table 3).

Global warming potential (GWP): GWP was not significantly different among manure treatments, although higher GWP (17.2 Mt CO_2-eq. ha^{-1}) was observed in the compost treatment followed by the control (16.3 Mt CO_2-eq. ha^{-1}) and cow dung treatment (14.6 Mt CO_2-eq. ha^{-1}) (Fig 5a). There was a significant difference in GWP among the varieties ($P<0.0050$). A higher GWP was observed for Manawthukha (19.5 Mt CO_2-eq.ha^{-1}) than for IR-50 (12.5 Mt CO_2-eq. ha^{-1}).

Rice yield: Grain yield was not significantly affected by the manure treatments. However, the numerically highest grain yield (122.3±2.4 g $plant^{-1}$) was recorded from the control (no manure), followed by the compost (102.6±13.6 g $plant^{-1}$) and cow dung treatments (79.6±15.6 g $plant^{-1}$). There was significant different in grain yield between the varieties Manawthukha (121.5±15.0 g $plant^{-1}$) and IR-50 (81.6±8.0 g $plant^{-1}$) ($P<0.0164$) (Table 3).

Greenhouse gas intensity: Greenhouse gas intensity was not affected by manure management and varieties (Table 3). However, across manure management treatments, higher GHGI values were found in Manawthukha (3.8 kg CO_2-eq. kg^{-1} grain) than in IR-50 (3.5 kg CO_2-eq. kg^{-1} grain) (Fig 5b). There was no interaction between manure and rice varieties on the GHGI.

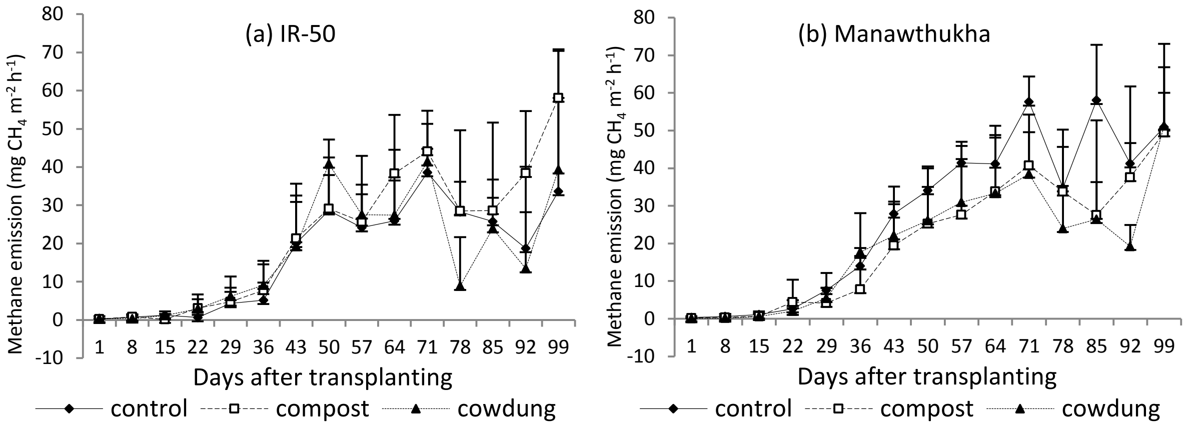

Fig 3. Methane emission of rice varieties at Yezin Agricultural University during the wet season, 2016. Mean value±standard deviation (n=3).

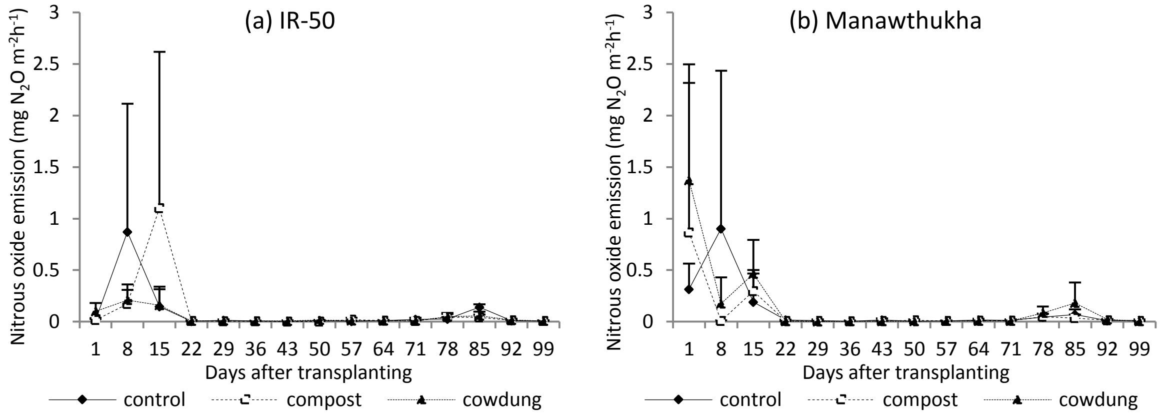

Fig 4. Nitrous oxide emission of rice varieties at Yezin Agricultural University during the wet season, 2016. Mean value±standard deviation (n=3).

Table 3. Effects of manure and rice variety on greenhouse gases emission and grain yield of rice during wet season, 2016

Treatment	CH_4 (g kg^{-1} soil)	N_2O (mg kg^{-1} soil)	Grain yield (g plant^{-1})	GHGI (kg CO_2-eqv kg^{-1} grain)
Manure				
Control	0.893	3.218	122.3± 2.4 a	2.8
Compost	0.951	2.491	102.6±13.6 ab	3.8
Cow dung	0.808	2.135	79.6±15.6 b	4.3
LSD $_{0.05}$	0.307	3.490	38.2	1.7
Variety				
Manawthukha	1.084 a	2.585	121.5±15.0 a	3.8
IR-50	0.683 b	2.644	81.6±8.0 b	3.5
LSD $_{0.05}$	0.251	2.849	31.2	1.4
Pr>F				
Manure	0.6091	0.7922	0.0901	0.2332
Variety	0.0046	0.9649	0.0164	0.6719
Manure*Variety	0.6724	0.5766	0.6633	0.9931
CV (%)	27.67	106.09	29.92	38.66

Within each column, values with different alphabets indicate significant differences among the treatments at 5% of LSD test.

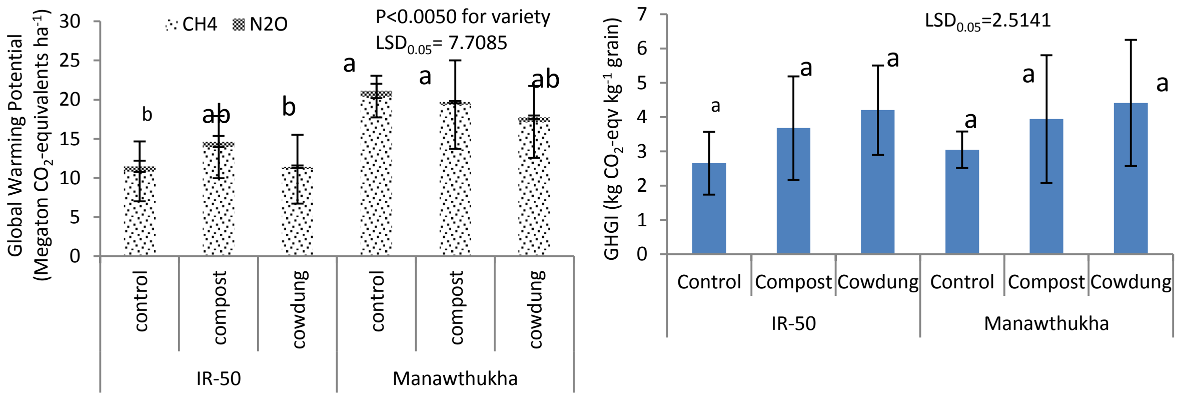

(a) Global warming potential (b) Greenhouse gas intensity

Fig 5. Effect of organic manure and rice varieties on (a) global warming potential and (b) greenhouse gas intensity during wet season, 2016. Mean value±standard deviation (n=3).

Second pot experiment during the dry and wet seasons, 2017

Methane emission*:* The seasonal methane emissions of rice are shown in Fig 6 (dry season) and Fig 7 (wet season). In the dry season, high methane emissions were observed in the early growth stage and then decreased until harvest under both water regimes. In the wet season, a slight increase in emissions was recorded in the early growth stage, emissions peaked in the middle stage, and gradually decreased until harvest. There were significant differences in cumulative CH_4 emissions among water management practices ($P<0.0003$) (Table 4). Higher cumulative CH_4 emissions were observed under CF than AWD. Despite no significant difference among the cow dung manure rates, a generally higher amount of cow dung manure produced more methane emissions than lower rates (Fig 12a).

Nitrous oxide emission*:* The seasonal nitrous oxide emissions of rice are shown in Fig 8 (dry season) and Fig 9 (wet season). Relatively high nitrous oxide emissions were found in the early growth stage, and reduced emissions were found in the later growth stage in both seasons. There were significant different in nitrous oxide emissions among the water management practices in dry season ($P<0.0159$) but not significant in wet season (Table 4). AWD gave a relative high N_2O emission in both seasons. No significant difference was observed in N_2O emission among the manure rates in either season.

Global warming potential (GWP)*:* The GWP was significantly different among water management practices in both seasons ($P<0.0004$, $P<0.0000$). A higher GWPs (66.3 Mt CO_2-eq. ha^{-1} and 40.0 Mt CO_2-eq. ha^{-1}) were observed under CF than under AWD (20.1 Mt CO_2-eq. ha^{-1} and 13.7 Mt CO_2-eq. ha^{-1}) in the dry (Fig 10a) and wet seasons (Fig 10b), respectively. Generally, the large application of cow dung manure resulted in a higher GWP in both seasons (Fig 12b). The different rates of cow dung manure had no significant effect on the GWP in either season.

Rice yield: In this pot experiment, the grain yield was not significantly affected by water management and manure amendment in either rice season (Table 5). However, higher grain yields per plant were recorded with AWD than CF in both seasons. The manure rates had no significant effect on rice yield.

Greenhouse gas intensity: Greenhouse gas intensities were significantly different among the water management practices in both seasons ($P<0.0002$, $P<0.0000$) (Table 5). Significantly higher GHGI values were found under CF than under AWD in both seasons (Fig 11). No significant differences were found in GHGI values among the manure management practices. However, the higher amount of cow dung manure affected the GHGI values under CF irrigation, but the effect of manure was suppressed by AWD.

Water input and water saving: Water inputs of rice as affected by water and manure management are shown in Table 6. There was significantly different of water input among the water management practices in either rice season ($P<0.0001$, $P<0.0000$). CF was irrigated more than AWD. The organic manure increases the water holding capacity of the soil. Accordingly, the higher amount of cow dung manure used less water. Water saving of treatments is shown in Table 7. AWD saved water 13.6% over CF in no cow dung manure in dry season and 19.1% in wet season. Across the manure management, the water saving of rice mainly depends on water management practices.

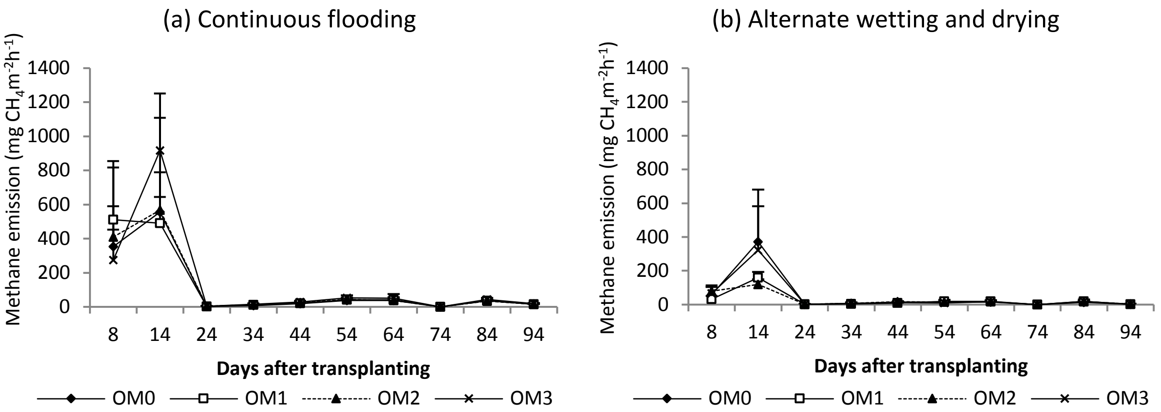

Fig 6. Methane emission from rice under (a) continuous flooding and (b) alternate wetting and drying during the dry season, 2017. Mean value±standard deviation (n=3).

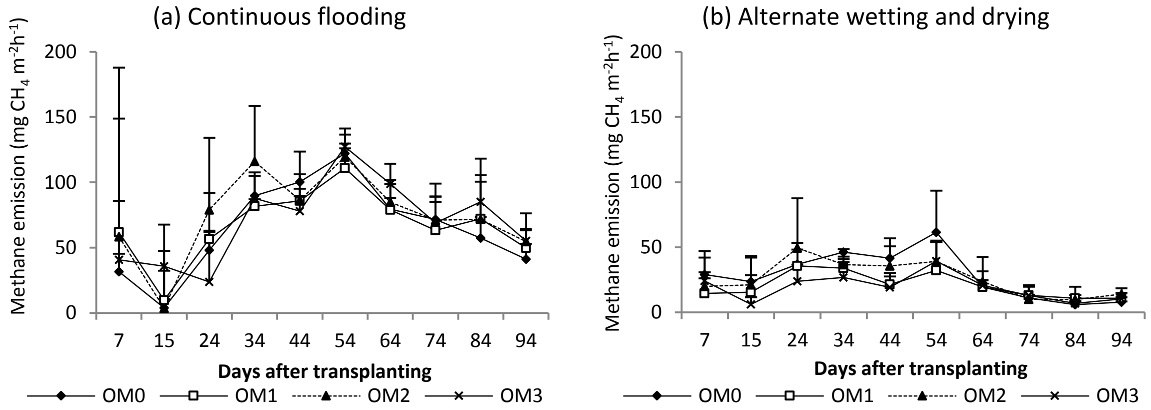

Fig 7. Methane emission from rice under (a) continuous flooding and (b) alternate wetting and drying during the wet season, 2017. Mean value±standard deviation (n=3).

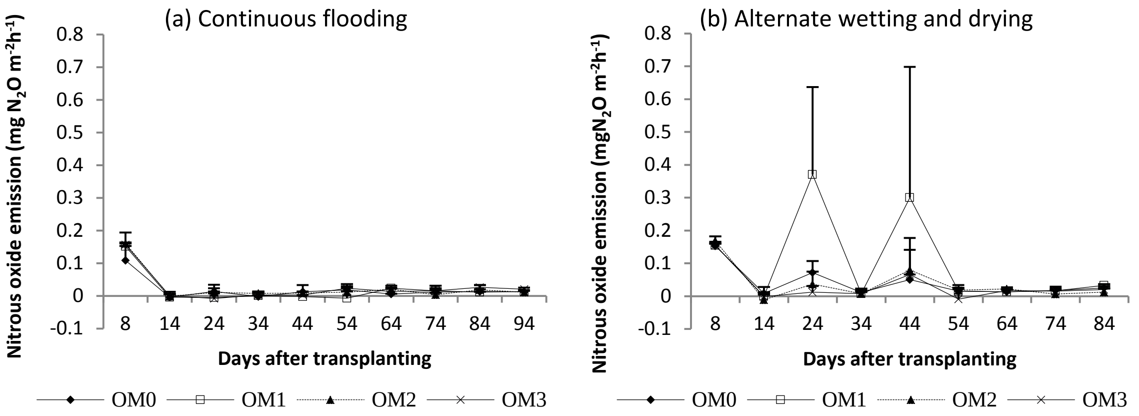

Fig 8. Nitrous oxide emission from rice under (a) continuous flooding and (b) alternate wetting and drying during the dry season, 2017. Mean value±standard deviation (n=3).

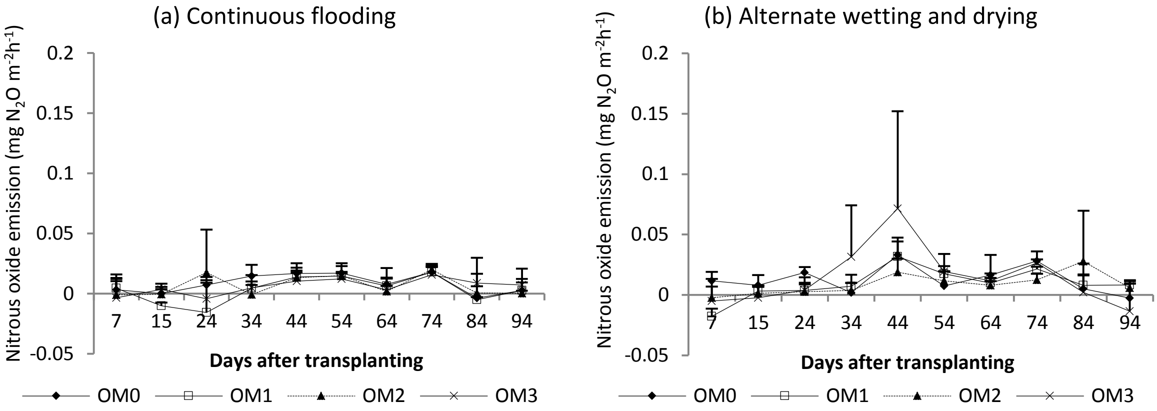

Fig 9. Nitrous oxide emission of rice under (a) continuous flooding and (b) alternate wetting and drying during the wet season, 2017. Mean value±standard deviation (n=3).

Table 4. Mean effects of water and manure management on greenhouse gases emission of rice during dry and wet seasons, 2017

Treatment	Cumulative methane emission ($g\ CH_4\ kg^{-1}$ soil)		Cumulative nitrous oxide emission ($mg\ N_2O\ kg^{-1}$ soil)	
	Dry season	Wet season	Dry season	Wet season
Water				
CF	3.327 a	2.009 a	0.590 b	0.183
AWD	0.996 b	0.682 b	1.392 a	0.344
$LSD_{0.05}$	1.089	0.369	0.631	0.176
Manure				
OM_0 ($0\ t\ ha^{-1}$)	2.129	1.361	0.786	0.306
OM_1 ($2.5\ t\ ha^{-1}$)	2.036	1.279	1.621	0.201
OM_2 ($5\ t\ ha^{-1}$)	2.007	1.460	0.817	0.234
OM_3 ($7.5\ t\ ha^{-1}$)	2.475	1.283	0.740	0.312
$LSD_{0.05}$	1.540	0.522	0.892	0.248
Pr>F				
Water	0.0003	0.0000	0.0159	0.0709
Manure	0.9114	0.8682	0.1546	0.7335
Water x Manure	0.9038	0.8217	0.0890	0.8582
CV (%)	58.21	31.74	73.53	77.08

Within each column, values with different alphabets indicate significant differences among the treatments at 5% of LSD test.

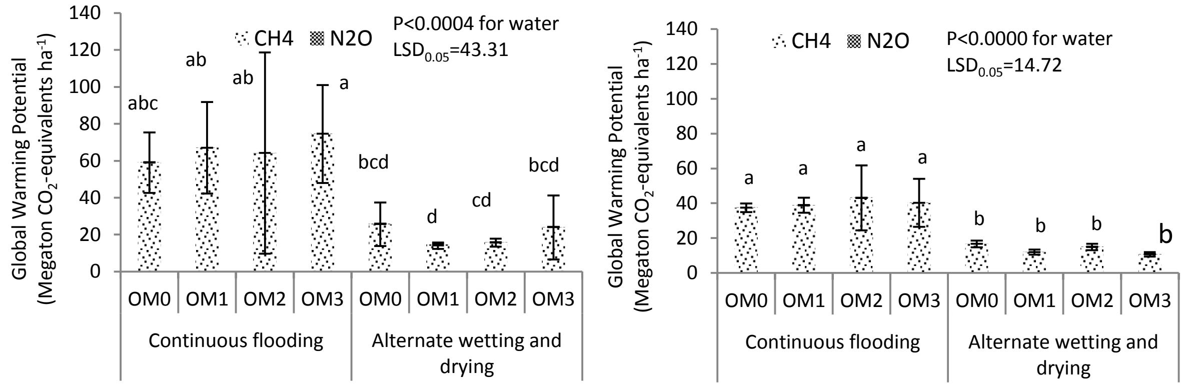

Fig 10. Effect of water and cow dung manure management on global warming potential of potted rice during dry (a) and wet (b) seasons, 2017. OM_0-no cow dung, OM_1- cow dung 2.5 t ha^{-1}, OM_2- cow dung 5.0 t ha^{-1}, OM_3- cow dung 7.5 t ha^{-1}. Mean value±standard deviation (n=3).

Table 5. Mean effects of water and manure management on rice yield during dry and wet seasons, 2017

Treatment	Yield (g $plant^{-1}$)		GHGI (kg CO_2-eq. kg^{-1} grain)	
	Dry season	Wet season	Dry season	Wet season
Water				
CF	175.06±6.3	180.88±4.9	7.4 a	4.3 a
AWD	177.73±6.8	183.47±6.7	2.1 b	1.4 b
$LSD_{0.05}$	12.67	14.27	2.3	0.7
Manure				
OM_0 (0 t ha^{-1})	181.54±9.4	183.70±3.1	4.5	2.9
OM_1 (2.5 t ha^{-1})	171.79±3.2	178.81±0.1	4.7	2.7
OM_2 (5 t ha^{-1})	178.08±11.0	179.20±0.1	4.3	3.1
OM_3 (7.5 t ha^{-1})	174.16±5.0	187.01±7.6	5.6	2.7
$LSD_{0.05}$	17.92	20.18	3.2	1.1
Pr>F				
Water	0.6608	0.7060	0.0002	0.0000
Manure	0.6758	0.7984	0.8359	0.8283
Water x Manure	0.3976	0.6511	0.7742	0.8092
CV (%)	8.30	9.05	55.13	31.13

Within each column, values with different alphabets indicate significant differences among the treatments at 5% of LSD test.

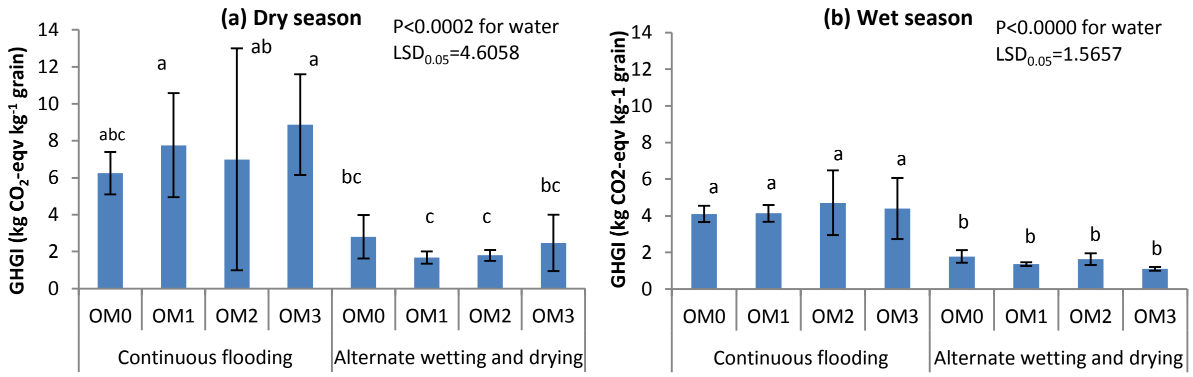

Fig 11. Effect of water and cow dung manure management on greenhouse gas intensity of potted rice during dry (a) and wet (b) seasons, 2017. OM_0-no cow dung, OM_1-cow dung 2.5 t ha^{-1}, OM_2- cow dung 5.0 t ha^{-1}, OM_3- cow dung 7.5 t ha^{-1}. Mean value±standard deviation (n=3).

Table 6. Mean effects of water and manure management on water input of rice during dry and wet seasons, 2017

Treatment	Water input $_{(I+R)}$ (mm)	
	Dry season	Wet season
Water		
CF	1075.5 a	842.1 a
AWD	969.8 b	681.4 b
LSD $_{0.05}$	42.4	32.7
Manure		
OM_0	1030.5	775.0
OM_1	1017.6	750.5
OM_2	1015.4	740.6
OM_3	1027.1	780.9
LSD $_{0.05}$	60.0	46.2
Pr>F		
Water	0.0001	0.0000
Manure	0.9385	0.2372
Water x Manure	0.0955	0.7619
CV (%)	4.80	4.96

Within each column, values with different alphabets indicate significant differences among the treatments at 5% of LSD test.

Table 7. Comparison of water saving of rice as affected by water and cow dung manure management during dry and wet seasons, 2017

Treatment		Water saving (%)	
Water	Cow dung manure	Dry season	Wet season
CF	OM_0		
CF	OM_1		
CF	OM_2		
CF	OM_3		
AWD	OM_0	13.6	19.1
AWD	OM_1	7.5	18.7
AWD	OM_2	2.8	17.1
AWD	OM_3	14.9	21.2

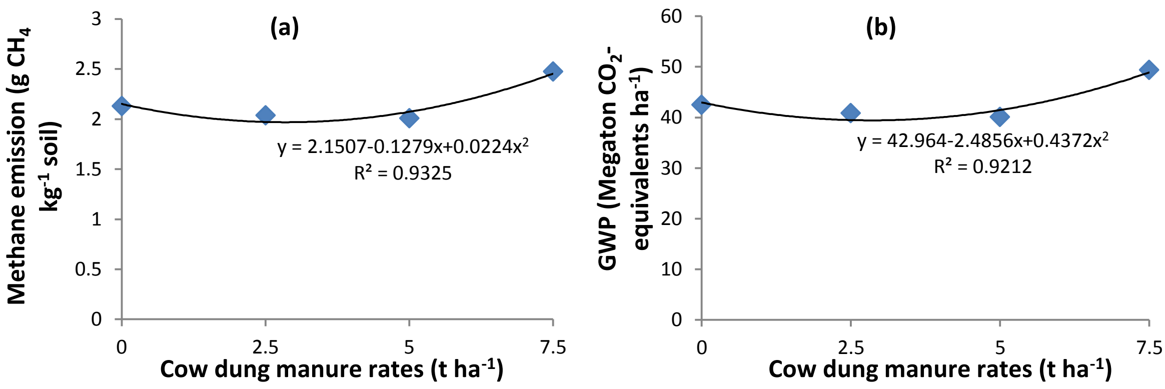

Fig 12. Relationship between methane emission and different cow dung manure rates (a) and between GWP and different cow dung manure rates (b) in dry season, 2017

Discussion

Effect of manure and rice varieties on greenhouse gas emissions, global warming potential and rice yield

Methane production and emissions from flooded paddies are highly affected by the addition of organic matter [47]. The higher CH_4 emission in this study was found in later growth stages (Fig 3) because the CH_4 emission was associated with higher soil organic matter with increased microbial activities, decomposition of plant residues from fallen leaves and decayed roots, and higher availability of root exudates in the rhizosphere [48]. Although there was no significant difference based on manure management, cow dung manure reduced methane emissions. This study also agrees with that of Oo et al. [30], who reported the lower CH_4 emissions from pots treated with well-decomposed cattle manure due to fewer carbon substrates with a reduction of potential CH_4 precursors resulting from the preceding decompostion. In contrast to CH_4 emissions, high N_2O emissions were recorded in the early growth stage, and small amounts of emissions were recorded in the later growth stage (Fig 4). This could be due to the rapid nitrification with the presence of oxygen and denitrification with the utilization of NO_3^- as electron acceptor in the initial stage with high temperature and low rainfall (Fig 1), and indigenous soil nitrogen (Table 1). After emission peaked initially, the rates of N_2O emission were generally low due to continuous flooding. Another study reported that the consistently low soil redox potential under continuous flooding resulted in more complete denitrification and thus reduced N_2O emissions [49]. The control (no manure) treatment resulted in higher N_2O emissions than the compost and cow dung treatments. Lower nitrous oxide emission was resulted by incorporation of organic inputs due to nitrogen immobilization [50-52]. This study agrees with that of Shan and Yan [53], who reported that N_2O emissions were significantly reduced by crop residue return combined with synthetic N fertilizers compared with emissions from treatments only received synthetic N fertilizer.

The selection of suitable rice varieties might play a significant role in regulating CH_4 emissions from rice fields [54]. The results of this study showed that there was a significant difference between the tested rice varieties (Fig 3 and Table 3). The result was in agreement with other findings, which highlighted that there were substantial differences in the rates of CH_4 emission among different rice varieties [30, 55, 56]. As ninety percent of methane emissions to the atmosphere are through rice plants [57], the Manawthukha variety, which has a longer growth duration, emitted more CH_4 than IR-50, which has a shorter growth duration. Previous studies [42, 58-60] have also reported that the CH_4 flux from late maturing rice is higher than that from early maturing rice. According to this research finding, a shorter growth duration (IR-50) variety can be used to reduce methane emissions. When compared to the two rice varieties, IR-50 resulted in higher N_2O emissions than the Manawthukha variety. This could be due to the favourable effect of root exudates of IR-50 on the nitrification process in the soil. Gogoi and Baruah [25] reported that the main driving forces influencing N_2O emission in rice were soil NO_3-N, soil organic carbon. There was no interaction between manure and rice varieties. The compost and Manawthukha variety emitted higher CH_4 due to longer decomposing time and support of substrates for methanogens. The control treatment and IR-50 variety resulted in higher N_2O emission due to nitrification and denitrification of inorganic nutrient in the favour of root exudates (Fig 5a).

Cow dung manure resulted in a 10.4% reduction in GWP compared to the control (no manure) with reduced CH_4 and N_2O emission. Combination of decomposed cow dung manure + mineral fertilizer might suppress the available carbon and nitrogen for CH_4 and N_2O production. Unfortunately, in our study, we couldn't measure the carbon and nitrogen at every gas sampling and their mechanisms. While an increased GWP (5.5%) was found in the compost compared to the control. Huang et al. [61] reported that incorporation of organic residues provides a source of readily available C and N in the soil and subsequently

influences N_2O emissions. Manawthukha had a higher GWP (56%) than IR-50. These results were supported by Zheng et al. [62], who reported that yield-scaled GWP at 80-90 days of growth duration after transplanting was 87% higher than that at 70-80 days of growth duration after transplanting. Feng et al. [63] also reported that yield-scaled GWP in late rice in a double-rice cropping system was 73% higher than that for varieties with 90-100 days of growth duration after transplanting.

A high grain yield of rice was recorded from the control due to the mineralization of indigenous soil organic matter and inorganic fertilizer (Table 3). Oo et al. [64] reported a high grain yield under inorganic sources of nutrients due to the immediate release and availability of nutrients. Relative to IR-50, Manawthukha had a higher grain yield by 48.9% whereas the GWP was reduced in IR-50 than in Manawthukha. Therefore, considering the mitigation practice for GWP from rice production, IR-50 and cow dung manure was experimented in 2017 rice seasons.

Effect of water management and manure application on greenhouse gas emissions, global warming potential and rice yield

In the dry season, high methane emissions were observed in the early growth stage and then decreased until harvest in both water regimes (Fig 6). The early increase in CH_4 emission was due to the indigenous soil carbon content and availability of substrates, and the decrease in the later growth stage was due to the senescence of older leaves and non-availability of substrate as the crop approached maturity [65-67]. In the wet season, a slight increase in emission was recorded in the early growth stage, and emission was highest in the middle stage and gradually decreased until harvest (Fig 7). A higher rate of CH_4 production is attributed to the availability of organic substrates from the previous crop residues in the form

of plant-derived C through processes such as root exudation and release of fallen leaves and intensively reduced conditions in the rice rhizosphere [54, 68]. The gradual decrease in CH_4 emission in later growth stage was due to the decomposition and non-availability of substrates depending on temperature (Fig 2). Higher cumulative CH_4 emissions were observed under CF than under AWD in both seasons because the anoxic conditions increased the methanogen population and favoured methane production. The methane emission was higher in the dry season than in the wet season because it depended on the availability of indigenous soil carbon and decomposition process favoured by high temperature in the dry season, and depletion of soil carbon and previous crop resides decomposition hindered by low temperature in wet season (Fig 2). Relatively high nitrous oxide emissions were observed in the early growth stage, and reduced emissions were observed in the later growth stage in both seasons (Fig 8 and 9). This was due to the indigenous soil nitrogen content in the early stage, and depletion of nutrients and the available nitrogen content for nitrification and denitrification in the later stage. Higher N_2O emissions were observed under AWD than CF in both seasons [54, 69, 70]. AWD increased N_2O emissions by 135% compared to CF in dry season and 88% in wet season. Similar to the CH_4 emission pattern, higher N_2O emissions were observed in the dry season than in the wet season because of the soil nitrogen availability and the favoured soil condition (high temperature and low rainfall) for nitrification and denitrification in the dry season [71, 72].

Generally higher rates of cow dung manure resulted in higher CH_4 and N_2O emission because it provided carbon and nitrogen sources for methanogenesis and nitrification and denitrification process. Methane is produced by methanogenic bacteria during the anaerobic digestion of organic substrates [4] and N_2O production is observed by soil water content and availability of substrates (nitrate and easily degradable organic matter) for denitrification [73]. However, the polynomial distribution of methane emission with different cow dung

manure rates (Fig 12a) and polynomial distribution of GWP with different cow dung manure rates (Fig 12b) were observed in dry season. The polynomial regression equation for methane emission is $y = 2.1507-0.1279x+0.0224x^2$. The equation showed that the methane emission decreased in the rate of 0.1279 g CH_4 kg^{-1} soil at every increased unit (ton) of cow dung manure applied, after that increased in the rate of 0.0224 g CH_4 kg^{-1} soil at every increased unit (ton) of cow dung manure applied. The coefficient of determination (R^2) showed that 93% of variation in methane emission could be accounted by the quadratic regression equation of different cow dung manure rates. The polynomial regression equation for GWP is $y = 42.964-2.4856x+0.4372x^2$. The equation showed that the GWP decreased in the rate of 2.4856 Mt CO_2-equivalent ha^{-1} at every increased unit (ton) of cow dung manure applied, after that increased in the rate of 0.4372 Mt CO_2-equivalent ha^{-1} at every increased unit (ton) of cow dung manure applied. The coefficient of determination (R^2) showed that 92% of variation in GWP could be accounted by the quadratic regression equation of different cow dung manure rates. According to these results, it would be recommended that 5 t ha^{-1} should be applied for improving soil fertility and reduced greenhouse gas emission. No interactive effect between water management and the application of different cow dung manure rates was found on CH_4 emissions in either season. However, the effect of the application of different cow dung manure rates on N_2O emissions was influenced by alternate wetting and drying irrigation practices in the dry season but not in the wet season. Therefore, the nitrification and denitrification processes of soil are influenced by the soil moisture content [74-77].

A higher GWP was observed under CF in accordance with the higher methane emissions in both seasons (Fig 10). Methane emission mainly contributes to the GWP from paddy production. Many studies [39, 54, 78-82] reported that N_2O emissions contribute much

less to the global warming potential than those of CH_4. Therefore, the water regime in paddy production is the main factor controlling CH_4 emissions from rice fields [39, 54, 83].

AWD resulted in a higher grain yield per plant than did CF in both seasons (Table 3.3) because it strengthened the air exchange between the soil and atmosphere and supplied sufficient oxygen to the root system to accelerate soil organic matter mineralization which increase soil fertility and favour rice growth [84, 85]. Yang and Zhang [86] reported an increase in paddy yield under AWD due to the increase in the proportion of productive tillers. In this study, AWD saved water about 10% over CF in dry season and 19% in wet season. Zhang et al. [87] also indicated water saving of 35% under AWD with a 10% yield increase relative to that under CF. Liu et al. [88], Ye et al. [89], and Djaman et al. [90] found that grain yield increased with reduced water input by AWD. Grain yield was not affected by the different rates of cow dung manure application because its decomposition was influenced by biotic and soil-environmental factors. Relative to the CF water management, AWD produced comparable grain yields, increased by 1.5% and decreased GHGI by 69% (Table 5). This suggests that by adopting alternate wetting and drying irrigation, it would be possible to achieve the dual goals of maintaining productivity while minimizing the global warming potential of rice cultivation.

Conclusion

For sustainable agriculture, organic manures should be added at a recommended amount to improve the rice yield and reduce greenhouse gas emissions. From our findings, the application of cow dung manure can be recommended in paddy production since it mitigated the global warming potential compared to that of the control and compost groups, although it resulted in a lower yield potential. Additionally, the short duration rice variety had a lower GWP and a lower GHGI value while maintaining the potential rice yield. Manure-induced greenhouse gas emissions were suppressed by AWD irrigation practices, with

significant GHGI values. Thus, short duration varieties are highly recommended with AWD irrigation and application of 5 t ha^{-1} cow dung manure to reduce greenhouse gas emissions and maintain rice yield under the soil-environmental conditions of Myanmar. In our study, there was no relationship between GWP and rice yield. Therefore, the choice of rice varieties should be combined with soil-environmental factors and cultivation systems to mitigate greenhouse gas emissions while increasing rice yields for sustainable rice production. Further studies under field condition are needed to measure the effect of manure and mineral fertilizer on greenhouse gas emission, global warming potential and rice yield under water management conditions for better understanding of emission mechanisms.

Acknowledgements

We thank Sonoko D. Bellingrath-Kimura and Aung Zaw Oo for technical advice and editing a previous manuscript version.

Reference

1. Burney JA, Davis SJ, Lobell DB. Greenhouse gas mitigation by agricultural intensification. Proc. Nat. Acad. Sci. U. S. A. 2010; 107: 12052–12057.
2. Tilman D, Balzer C, Hill J, Befort BL. Global food demand and the sustainable intensification of agriculture. Proc. Nat. Acad. Sci. USA. 2011; 108: 20260–20264.
3. Smith P, Martino D, Cai Z, Gwary D, Janzen H, Kumar P, et al. Agriculture. In: Metz, B., Davidson, O.R., Bosch, P.R., Dave, R., Meyer, L.A. (Eds.), Climate Change 2007: Mitigation. Contribution of Working Group III to the Fourth Assessment Report of the Intergovernmental Panel on Climate Change. Cambridge University Press, Cambridge, UK and New York, NY, USA, 2007; 497–540.
4. Mer JL and Roger P. Production, oxidation, emission and consumption of methane by soils: A review. Eur. J. Soil Biol. 2001; 37: 25-50.
5. Yan X, Akiyama H, Yagi K, Akimoto H. Global estimations of the inventory and mitigation potential of methane emissions from rice cultivation conducted using the

2006 Intergovernmental Panel on Climate Change Guidelines. Global Biogeochem. Cyc. 2009; 23, GB2002.

6. Zou J, Huang Y, Qin Y, Liu S, Shen Q, Pan G, et al. Changes in fertilizer-induced direct N2O emissions from paddy fields during ricegrowing season in China between 1950 and 1990. Global Change Biol., 2009; 15: 229–242.
7. Wang JY, Jia JX, Xiong ZQ, Khalil MAK, Xing GX. Water regime-nitrogen fertilizer-straw incorporation interaction: field study on nitrous oxide emissions from a rice agroecosystem in Nanjing. China. Agric. Ecosyst. Environ., 2011; 141: 437–446.
8. Meijide A, Manca G, Goded I, Magliulo V, di Tommasi P, Seufert G, et al. Seasonal trends and environmental controls of methane emissions in a rice paddy field in Northern Italy. Biogeosciences, 2011; 8: 3809-3821.
9. IPCC. Climate change 2007: synthesis report *Contribution of Working Groups I, II and III to the Fourth Assessment Report of the Intergovernmental Panel on Climate Change*, ed. By Pachauri, RK and Reisinger A. IPCC, Geneva, Switzerland, 2007; 104 p.
10. Forster P, Ramaswamy V, Artaxo P, Berntsen T, Betts R, Fahey DW, et al. Changes in atmospheric constituents and in radiative forcing. In: Solomon S, Qin D, Man-ning M, Chen Z, Marquis M, Averyt K, Tignor M, Miller H (Eds) Climate Change 2007: the physical science basis. Contribution of Working Group I to the Fourth Assessment Report of the Intergovernmental Panel on Climate Change. Cambridge University Press: UK and New York pp 129–234.
11. IPCC: Intergovernment Panel on Climate Change. 2013. Fifth Assessment Report (AR5) Contribution of Working Group III to Climate Change 2014: Mitigation of Climate Change.
12. Ma Y, Wang J, Zhou W, Yan X, Xiong Z. Greenhouse gas emissions during the seedling stage of rice agriculture as affected by cultivar type and crop density. Biol. Fertil. Soils 2012; 48: 589-595.
13. Win EP, Win KK, Bellingrath-Kimura SD, Oo AZ. Greenhouse gas emissions, grain yield and water productivity: a paddy rice field case study based in Myanmar. Greenhouse Gases: Science and Technology, 2020; 10: 884-897.
14. Liu S, Zhang Y, Lin F, Zhang L, Zou J. Methane and nitrous oxide emissions from direct-seeded and seedling-transplanted rice paddies in southeast China. Plant Soil, 2013. Available: http://dx.doi.org/10.1007/s11104-013-1878-7.

15. Ma J, Ji Y, Zhang G, Xu H, Yagi K. Timing of midseason aeration to reduce CH_4 and N_2O emissions from double rice cultivation in China. Soil Sci. Plant Nutr., 2013; 59: 35-45.
16. Das K. and Baruah K K. Association between contrasting methane emissions of two rice (*Oryza sativa* L.) cultivars from the irrigated agroecosystem of northeast India and their growth and photosynthetic characteristics. Acta Physiologiae Plantarum, 2008; 30: 569-578.
17. Setyanto P, Rosenani AB, Boer R, Fauziah CI and Khanif MJ. The effect of rice cultivars on methane emission from irrigated rice field. Indonesian J Agric Sci., 2004; 5: 20-31.
18. Eller G, Frenzel P. Changes in activity and community structure of methane oxidizing bacteria over the growth period of rice. Appl Environ Microbiol, 2001; 67: 2395–2403.
19. Dubey SK, Singh JS. Spatio-temporal variation and effect of urea fertilization on methanotrophs in a tropical dryland rice field. Soil Biol Biochem, 2000; 32: 221–226.
20. Ma KE, Qiu Q, Lu Y. Microbial mechanism for rice variety control on methane emission from rice field soil. Global Change Biol. 2010. doi:10.1111/j13652486200902145
21. Wang W, Lai DYF, Wang C, Tong C, Zeng C. Effects of inorganic amendments, rice cultivars and cultivation methods on greenhouse gas emissions and rice productivity in a subtropical paddy field. Ecological Engineering 2016; 95: 770-778.
22. Ciarlo E, Conti M, Bartoloni N, Rubio G. The effect of moisture on nitrous oxide emissions from soil and the $N_2O/(N_2O+N_2)$ ratio under laboratory conditions. Biol. Fertil. Soils 2007; 43: 675-681.
23. Liou SM, Lo SL, Wang SH. A generalized water quality index for Taiwan. Environmental Monitoring and Assessment, 2003; 96: 35-52.
24. Chen J, Qin G, Wang J, Yu J, Shen B, Li S, et al. One-step fabrication of sub-10-nm plasmonic nanogaps for reliable SERS sensing of microorganisms. Biosens Bioelectron, 2013; 44: 191-197.
25. Gogoi B and Baruah KK. Nitrous oxide emissions from fields with different wheat and rice varieties. Pedosphere 2012; 22(1): 112-121.
26. Mariko S, Harazono Y, Owa N, Nouchi I. Methane in flooded soil water and the emission through rice plants to the atmosphere. Environ Exp Bot., 1991; 31: 343–350.

27. Wang B, Neue HU, Samonte HP. Effect of cultivar difference (‘IR72’, ‘IR65598’ and ‘Dular’) on methane emission. Agric Ecosyst Environ., 1997; 62: 31–40.
28. Xu YC, Wang ZY, Li Z, Wang BJ. Effect of rice cultivars on methame emission from Beijing rice field. Plant Nutr Fert Sci., 1999; 5: 93–96 (in Chinese with English abstract).
29. Aulakh MS, Bodenbender J, Wassmann R, Rennenberg H. Methane transport capacity of rice plants, II. Variations among different rice cultivars and relationship with morphological characteristics. NutrCycling Agroecosyst. 2000; 58: 367–375.
30. Oo AZ, Win KT, Motobayashi T and Bellingrath-Kimura SD. Effect of cattle manure amendment and rice cultivars on methane emission from paddy rice soil under continuously flooded conditions. J. Environmental Biology, 2016; 37(5): 1029-1036.
31. Singh S, Kumar S, Jain MC. Methane emission from two Indian soils planted with different rice cultivars. Biol Fert Soils. 1997; 25: 285–289.
32. Khosa MK, Sidhu BS, Benbi DK. Effect of organic materials and rice cultivars on methane emission from rice field. J Environ Biol., 2010; 31: 281–285.
33. Yu J, Wang LL, Yan XJ, Tian YL, Deng AX, Zhang WJ. Super rice cropping will enhance rice yield and reduce CH4 emission: a case study in Nanjing. Chin Rice Sci., 2013; 20: 427–433.
34. Qin XB, Li YE, Wan YF, Liao YL, Fan MR, Gao QZ, et al. Effect of tillage and rice residue return on CH4 and N2O emission from double rice field. Trans Chin Soc Agric Eng., 2014; 30: 216–224 (in Chinese with English abstract).
35. Kongchum M. Effect of Plant Residue and Water Management Practices on Soil Redox Chemistry, Methane Emission, and Rice Productivity. A Dissertation, he Graduate Faculty of the Louisiana State University and Agricultural and Mechanical College, 2005.
36. Jan PL, Gerard LV, Wim de V, Johannes K. Differentiation of nitrous oxide emission factors for agricultural soils. Environmental Pollution, 2011; 159: 3215-3222.
37. Adhya TK, Bharati K, Mohanty SR, Ramakrishnan B, Rao VR, Sethunathan N, et al. Methane emission from rice fields at Cuttack, India. Nutrient Cycling in Agroecosystems, 2000; 58: 95-105.
38. Gathorne-Hardy A. A Life Cycle Assessment (LCA) of greenhouse gas emissions from SRI and flooded rice production in Southeast India, Taiwan Water Conservancy Journal, 2013; 61(4): 111-125.

39. Yan X, Yagi K, Akiyama H, Akimoto H. Statistical analysis of the major variables controlling methane emission from rice fields. Global Change Biol., 2005; 11(7): 1131–1141.
40. Alfred GH, Duv NR, Venkatanarayana M, Barbara HW. A Life Cycle Assessment (LCA) of greenhouse gas emissions from SRI and flooded rice production in SE India. Taiwan Water Conservancy, 2013; 61(4): 110-125.
41. Myanmar Agriculture Sector in Brief. Crop Sub-Sector. Department of planning, the Ministry of agriculture, livestock and irrigation, Nay Pyi Taw, Myanmar, 2019.
42. Oo AZ, Win KT, Bellingrath-Kimura SD. Within field spatial variation in methane emissions from lowland rice in Myanmar. Springer Plus. 2015; 4: 145. Available: http://dx.doi.org/10.1186/s40064-015-0901-2.
43. Ali MA, Kim PJ and Inubushi K. Mitigating yield-scaled greenhouse gas emissions through combined application of soil amendments: A comparative study between temperate and subtropical rice paddy soils. Science of the Total Environment, 2015;529: 140–148.
44. Xiong Z, Yinglie L, Wu Z, Huang T. Differences in net global warming potential and greenhouse gas intensity between major rice-based cropping systems in China. Scientific Report 2015; 5: 17774.
45. Pittelkow CM, Adviento-Borbe MA, Kessel C, Hill JE, Linquist BA. Optimizing rice yields while minimizing yield-scaled global warming potential. Global Change Biol. 2014; 20: 1382–1393.
46. Van Groenigen JW, Velthof GL, Oenema O, Van Groenigen KJ, Van Kessel C. Towards an agronomic assessment of N2O emissions: a case study for arable crops. Eur. J. Soil Sci. 2010; 61: 903–913.
47. Jean LM and Pierre R. Production, oxidation, emission and consumption of methane by soils: A review. Eur. J. Soil Biol., 2001; 37: 25-50.
48. Oo AZ, Nguyen L, Win KT, Cadisch G and Bellingrath-Kimura SD. Toposequential variation in methane emissions from double-cropping paddy rice in Northwest Vietnam. Geoderma, 2013; 209-210, 41-49.
49. Oo AZ, Sudo S, Inubushi K, Mano M, Yamamoto A, Ono K, et al. Methane and nitrous oxide emission from conventional and modified rice cultivation systems in South India. Agriculture, Ecosystems and Environment, 2018; 252: 148-158.

50. Zou J, Huang Y, Jiang J, Zheng X, Sass RL. A 3-year field measurement of methane and nitrous oxide emissions from rice paddies in China: Effects of water regime, crop residue, and fertilizer application. Global Biogeochem. Cycles, 2005; 19: GB2021.

51. Dalal RC, Gibson I, Allen DE, Menzies NW. Green waste compost reduces nitrous oxide emissions from feedlot manure applied to soil. Agric. Ecosyst. Environ. 2010; 136: 273-281.

52. Iqbal J, Parkin TB, Helmers MJ, Zhou X, Castellano MJ. Denitrification and nitrous oxide emissions in annual croplands, perennialgrass buffers, and restored perennial grasslands. Soil Sci. Soc. Am. J. 2015; 79: 239-250.

53. Shan J and Yan X. Effects of crop residue returning on nitrous oxide emissions in agricultural soils. Atmospheric Environment, 2013; 71: 170-175.

54. Oo AZ, Sudo S, Inubushi K, Chellappan U, Yamamoto A, Ono K, et al. Mitigation Potential and Yield-Scaled Global Warming Potential of Early-Season Drainage from a Rice Paddy in Tamil Nadu, India. Agronomy 2018; 8: 202; doi:10.3390/agronomy8100202

55. Kumar JIN and Viyol S. Short term diurnal and temporal measurement of methane emission in relation to organic carbon, phosphate and sulphate content of two rice fields of central Gujarat, India. Journal of Environmental Biology, 2009; 30(2): 241-246.

56. Koga N. and Tajima R. Assessing energy efficiencies and greenhouse gas emissions under bioethanol-oriented paddy rice production in northern Japan. Journal of Environmental Management, 2011; 92(3): 967-973.

57. Wang ZY, Xu YC, Li Z, Guo YX, Wassmann R, Neue N, et al. A four year record of methane emission from irrigated rice fields in Beijing region of China, Nutr Cycling Agroecosys, 2000; 58: 55-63.

58. Cai ZC, Tsuruta H, Minami K. Methane emission from rice fields in China: measurements and influencing factors. J. Geophys. Res. 2000; 105 (D13): 17231–17242.

59. Satyanto P, Makarim AK, Fagi AM, Wassmann R and Buendia LV. Crop management affecting methane emissions from irrigated and rainfed rice in Central Jawa (Indonesia), Nutr Cycling Agroecosys, 2000; 58: 85-93.

60. Yang X, Shang Q, Wu P, Liu J, Shen Q, Guo S, et al. Methane emissions from double rice agriculture under long-term fertilizing systems in Hunan, China. Agric. Ecosyst. Environ. 2010; 137: 308–316.

61. Huang Y, Zou J, Zheng X, Wang Y. and Xu X. Nitrous oxide emissions as influenced by amendment of plant residues with different C/N ratios. Soil Biology and Biochemistry, 2004; 36: 973-981.
62. Zheng H, Huang H, Yao L, Liu J, He H, Tang J. Impacts of rice varieties and management on yield-scaled greenhouse gas emissions from rice fields in China: A meta-analysis. Biogeosciences, 2014; 11: 3685-3693.
63. Feng JF, Chen CQ, Zhang Y, Song ZW, Deng AX, Zheng CY, et al. Impacts of cropping practices on yield-scaled greenhouse gas emissions from rice fields in China: a meta-analysis, Agr. Ecosyst. Environ., 2013; 164: 220–228.
64. Oo AN, Banterng P, Polthanee A and Trelo-Ges V. The Effect of Different Fertilizers Management Strategies on Growth and Yield of Upland Black Glutinous Rice and Soil Property. Asian Journal of Plant Sciences, 2010; 9: 414-422.
65. Das S, Adhya TK. Effect of combine applica-tion of organic manure and inorganic fertilizer on me-thane and nitrous oxide emissions from a tropical flooded soil planted to rice. Geoderma, 2014; 213: 185-192.
66. Borah L, Baruah KK. Effects of foliar application of plant growth hormone on methane emission from tropical rice paddy. Agriculture Ecosystems and Environment 2016; 233:75–84.
67. Baruah KK, Gorh D, Gogoi N. Analysis of greenhouse gas (Methane and Nitrous oxide) emission and global warming potential from rice fields: with reference to biological mitigation of climate change. SDRP Journal of Earth Sciences & Environmental Studies, 2018; 3(2): 1-13.
68. Kell DB. Large-scale sequestration of atmos-pheric carbon via plant roots in natural and agricultural ecosystems: why and how. Philos. Trans. R. Soc. B. 2012; 367:1589–1597.
69. Hou AX, Chen GX, Wang ZP, Van Cleemput O, Patrick Jr, WH. Methane and nitrous oxide emissions from a rice field in relation to soil redox and microbiological process. Soil Sci. Soc. Am. J. 2000; 64: 2180–2186.
70. Jain N, Dubey R, Dubey DS, Singh J, Khanna M, Pathak H, et al. Mitigation of greenhouse gas emission with system of rice intensification in the Indo-Gangetic Plains. Paddy Water Environ., 2014; 12(3): 355–363.
71. Dalal RC, Wang WJ, Robertson GP, Parton WJ. Nitrous oxide emission from Australian agricultural lands and mitigation options: A review. Aust. J. Soil Res., 2003; 41: 165–195.

72. IPCC (Intergovernmental Panel on Climate Change). Guidelines for National Greenhouse Gas Inventories. In National Greenhouse Gas Inventories Programme; Eggleston HS, Buendia L, Miwa K, Ngara T, Tanabe K, Eds.; IGES: Hayama, Kanagawa, Japan, 2006.
73. Dobbie KE, McTaggart IP and Smith KA. Nitrous oxide emissions from intensive agricultural systems: variations between crops and seasons, key driving variables, and mean emission factors. J. Geophys. Res. 1999; 104: 26891–26899.
74. Simojoki A and Jaakkola A. Effect of nitrogen fertilization, cropping and irrigation on soil air composition and nitrous oxide emission in a loamy clay. Eur. J. Soil Sci. 2000; 51: 413–424.
75. Dobbie KE and Smith KA. The effects of temperature, water-filled pore space and land use on N2O emissions from an imperfectly drained gleysol. Eur. J. Soil Sci. 2001; 52: 667–673.
76. Simojoki A. Oxygen supply to plant roots in cultivated mineral soils. Pro Terra No. 7, Helsinki, Finland, 2001; 59 pp. Dissertation, pdf version available at http://ethesis.helsinki.fi_.
77. Skiba U and Ball B. The effect of soil texture and soil drainage on emissions of nitric oxide and nitrous oxide. Soil Use Manage. 2002; 18: 56–60.
78. Kurosawa M, Yamashiki Y, Tezuka T. Estimation of energy consumption and environmental impact reduction by introducing sustainable agriculture to rice paddy field cultivation. J. Life Cycle Ass. Japan 2007; 3(4): 232–238.
79. Itoh M, Sudo S, Mori S, Saito H, Yoshida T, Shiratori Y, et al. Mitigation of methane emissions from paddy fields by prolonging midseason drainage. Agric. Ecosyst. Environ., 2011; 141: 359–372.
80. Pittelkow CM, Adviento-Borbe MA, Hill JE, Six J, van Kassel C, Linquist B. Yield-scaled global warming potential of annual nitrous oxide and methane emissions from continuously flooded rice in response to nitrogen input. Agric. Ecosyst. Environ., 2013; 177: 10–20.
81. Sander BO, Wassmann R, Siopongco JDLC. Mitigating greenhouse gas emissions from rice production through water-saving techniques: potential, adoption and empirical evidence. In: Hoanh, C., Smakhtin, V., Johnston, R. (Eds.), Climate Change and Agricultural Water Management in Developing Countries. CAB International, Oxfordshire, UK, 2015; 193–207.

82. Tarlera S, Capurro MC, Irisarri P, Scavino AF, Cantou G, Roel A. Yieldscaled global warming potential of two irrigation management systems in a highly productive rice system. Sci. Agric. 2016; 2: 43–50.
83. Wu X, Wang W, Xie X, Yin C, Hou H, Yan W, et al. Net global warming potential and greenhouse gas intensity as affected by different water management strategies in Chinese double rice-cropping systems. Scientific reports, 2018; 8: 779.
84. Dong NM, Brandt KK, Sørensen J, Hung NN, Hach CV, Tan PS, et al. Effects of alternating wetting and drying versus continuous flooding on fertilizer nitrogen fate in rice fields in the Mekong Delta, Vietnam. Soil Biol. Biochem. 2012; 47: 166–174.
85. Tan X, Shao D, Liu H, Yang F, Xaio C, Yang H. Effects of alternate wetting and drying irrigation on percolation and nitrogen leaching in paddy fields. Paddy Water Env., 2013; 11: 1–15.
86. Yang JC, Zhang JH. Grain-filling problem in 'super' rice. J. Exp. Bot. 2010; 61: 1–5.
87. Zhang H, Xue Y, Wang Z, Yang J, Zhang J. Alternate wetting and moderate soil drying improves root and shoot growth in rice. Crop Sci. 2009; 49: 2246–2260.
88. Liu L, Chen T, Wang Z, Zhang H, Yang J, Zhang J. Combination of site-specific nitrogen management and alternate wetting and drying irrigation increases grain yield and nitrogen and water use efficiency in super rice. Field Crops Res. 2013; 154: 226–235.
89. Ye M, Song Y, Long J, Wang R, Baerson SR, Pan Z, et al. Priming of jasmonate-mediated antiherbivore defense responses in rice by silicon. Proc. Natl. Acad. Sci. USA 2013; 110: 3631-3639.
90. Djaman K, Mel VC, Diop L, Sow A, El-Namaky R, Manneh B, et al. Effects of AlternateWetting and Drying Irrigation Regime and Nitrogen Fertilizer on Yield and Nitrogen Use Efficiency of Irrigated Rice in the Sahel. Water, 2018; 10: 711.

Click here to access/download
Supporting Information
NZSGJQ41_200929_GHG_text_final.docx

Influence of rice varieties, organic manure and water management on greenhouse gas emissions from paddy rice soils

Ei Phyu Win[1], Kyaw Kyaw Win[1], Sonoko D. Bellingrath-Kimura[2], Aung Zaw Oo[3]

[1]Department of Agronomy, Yezin Agricultural University, Myanmar
[2]Institute of Land Use Systems, Faculty of Life Science, Humboldt-Universität zu Berlin, Germany
[3]Japan International Research Center for Agricultural Sciences, Ohwashi, Tsukuba, Ibaraki, Japan

Abstract

The study is focused on impact of manure application, rice varieties and water management on greenhouse gas (GHG) emissions from paddy rice soil in pot experiment. The objectives of this study were a) to assess the effect of different types of manure amendments and rice varieties on greenhouse gas emissions and b) to determine the optimum manure application rate to increase rice yield while mitigating GHG emissions under alternate wetting and drying irrigation in paddy rice production. The first pot experiment was conducted at the Department of Agronomy, Yezin Agricultural University, Myanmar, in the wet season from June to October 2016. Two different organic manures (compost and cow dung) and control (no manure), and two rice varieties; Manawthukha (135 days) and IR-50 (115 days), were tested. The results showed that cumulative CH_4 emission from Manawthukha (1.084 g CH_4 kg^{-1} soil) was significantly higher than that from IR-50 (0.683 g CH_4 kg^{-1} soil) ($P<0.0046$) with yield increase ($P<0.0164$) because of the longer growth duration of the former. In contrast, higher cumulative nitrous oxide emissions were found for IR-50 (2.644 mg N_2O kg^{-1} soil) than for Manawthukha (2.585 mg N_2O kg^{-1} soil). However, IR-50 showed less global warming potential (GWP) than Manawthukha ($P<0.0050$). Although not significant, the numerically lowest CH_4 and N_2O emissions were observed in the cow dung manure treatment (0.808 g CH_4 kg^{-1} soil, 2.135 mg N_2O kg^{-1} soil) compared to those of the control and compost. To determine the effect of water management and organic manures on greenhouse gas emissions, second pot experiments were conducted in Madaya township during the dry and wet seasons from February to October 2017. Two water

Commented [T1]: I just revised it.

Commented [T2]: I put p value for their significance.

management practices {continuous flooding (CF) and alternate wetting and drying (AWD)} and four cow dung manure rates {(1) 0 (2) 2.5 t ha^{-1} (3) 5 t ha^{-1} (4) 7.5 t ha^{-1}} were tested. The different cow dung manure rates did not significantly affect grain yield or greenhouse gas emissions in this experiment. Across the manure treatments, AWD irrigation significantly reduced CH_4 emissions by 70% during the dry season and 66% during the wet season. Although a relative increase in N_2O emissions under AWD was observed in both rice seasons, the global warming potential was significantly reduced in AWD compared to CF in both seasons ($P<0.0002$, $P<0.0000$) according to reduced emission in CH_4. Therefore, AWD is the effective mitigation practice for reducing GWP without compromising rice yield while manure amendment had no significant effect on GHG emission from paddy rice field. Besides, AWD saved water about 10% in dry season and 19% in wet season.

Commented [T3]: Conclusion and recommendation.

Keywords: mitigation, greenhouse gas emissions, rice varieties, organic fertilizer, water management

*Corresponding author

E-mail: eiphyuwin@yau.edu.mm (EP)

Introduction

Developing new strategies is necessary to achieve the dual goals of ensuring food security and protecting natural resources and the environment through reduced greenhouse gas (GHG) emissions [1, 2]. It is estimated that nitrous oxide (N_2O) and methane (CH_4) emissions may increase by 35-60% and 60%, respectively, by 2030 [3]. Flooded rice soils are an important source of global CH_4 emissions [4, 5], and rice-based cropping systems can emit substantial amounts of N_2O [6] during the rice season itself [7].

Rice is one of the most important cereal grains and staple food crops globally and is particularly important in Asia [8]. Rice paddies contribute to the emission of the two most important GHGs; methane and nitrous oxide. IPCC [9] reported that rice fields contribute about 30% and 11% of global agricultural CH_4 and N_2O emissions, respectively. With a

linearly increasing rate of 0.26% per year during the recent few decades, the atmospheric N_2O concentration has increased by 18% compared to the preindustrial level. Methane and nitrous oxide have long atmospheric lifetimes of 12 and 114 years, respectively, and account for 20% and 7%, respectively, of global radiative forcing [10]. The high global warming potential (GWP) of CH_4 and N_2O, 34 and 298 times that of CO_2 at a 100-year time horizon, makes them major contributors to climate change [11]. In recent years, suitable management practices have been developed for achieving both improvement in rice yields and mitigation of GHG emissions, which include the development of new rice varieties [12], the application of manure such as cow dung [13], the selection of appropriate cultivation methods [14] and the timing of drainage [15].

The magnitude of CH_4 emissions from rice plants is regulated by complex and dynamic interactions among the plants, environment, and microorganisms [16]. Methane produced in flooded rice soils is emitted to the atmosphere by molecular diffusion, ebullition or plant-mediated transport. Approximately 80-90% of the total CH_4 flux is emitted to the atmosphere from the rhizosphere via the rice plant [17]. An increase in plant biomass [18] and tiller number [19] enhanced CH_4 oxidization activity by enlarging the volume of aerenchyma and enhancing O_2 transport from the atmosphere to the rhizosphere. Ma et al. [20] revealed that a hybrid rice variety with 50–60% higher shoot biomass emitted less CH_4 than an indica rice variety, possibly due to higher CH_4 oxidization activity.

Nitrous oxide is produced as a by-product of nitrification, denitrification, nitrifier denitrification, etc. and moisture content is a key factor governing N_2O production in soils [21]. Ciarlo et al. [22] found that denitrification is dominant pathway for N_2O emission when the water-filled pore space in soils is high (80%), and if the soils were saturated than this level, most of N_2O would be reduced to nitrogen.

Selecting a rice variety that has high productivity and low GHG emissions is crucial for improving crop yield and mitigating climate change; however, research examining the effects of rice varieties has mostly focused on CH_4 flux so far [12, 23, 24], with little focus on N_2O flux [25]. Many studies reported that the effect of rice varieties on methane emissions is mostly related to rice growth performance, i.e., the number of plant tillers and above- and belowground biomass, root exudates and root arenchyma [26-30]. Although significant positive relationship have been found between rice biomass and methane fluxes [31, 32], a comparison of rice varieties has produced different results [33, 34].

Organic residue amendments have been practised to improve soil fertility in paddy production. The organic matter in paddy fields originates from both direct by-products of rice production (such as sloughed-off root cells and root exudates) and added materials (manures and previous crop residues). The addition of organic carbon to the soil, whether it comes from the disposal of crop residues or as organic fertilizer, appears to be the most important factor in methane production [32]. Waterlogged conditions are ideal for the decomposition of organic matter in paddy fields. The methane production from rice soil can be increased by addition of cow manure as a source of organic material [35]. Nitrous oxide emissions from applied fertilizer and manures can vary with different environmental factors (e.g., climate and soil conditions), crop factors (e.g., crop type and crop residues), and management practices (e.g., type of manure and fertilizer, application rate, time of application) [36].

Although there are wide range of factors influencing methane emission from paddy soil [37-39], water management and organic amendment are the two main drivers of methane production. Under anaerobic soil conditions, methanogens (methane-producing bacteria) produce methane by oxidation of organic matter during anaerobic respiration. Flooded rice soils are known to have strong denitrification activity emitting some amount of N_2O from rice soils. However, it is also guided by the water management conditions of the rice field. Nitrate

and nitrite in rice soils is limited due to submerged conditions. The oxygen supply due to decomposition of organic matter, roots, and also through vascular transport via tillers may help in production of nitrate in rice soils. Sometimes due to prolonged submergence of the rice fields, the soil nitrate and nitrite N (available due to mineralization of organic matter) is completely reduced to N_2 gas thereby resulting in low N_2O emission. This varies with the prevailing conditions of the rice field. Although minimal N_2O emissions are likely from flooded soils, some off-site (indirect) N_2O emissions are likely from irrigated rice production due to the addition of nitrogen fertilizer to fields [40].

Commented [T4]: Not related to farming but it related to transportation of fertilizer, and fertilizer industry.

Myanmar ranks sixth in the world in rice production. Rice is the country's most important crop and is grown on 7.3 million ha [41]. The conventional rice production method commonly used by the farmers in Myanmar includes transplanting old seedlings (30-45) under continuous flooding conditions and the intensive use of organic fertilizers such as manure or compost. However, there is very limited information on methane emissions from the flooded rice fields of Myanmar, although more than half of the cultivated area is devoted to rice production [13, 42]. Thus, the objectives of this study were a) to assess the effect of different types of manure amendments and rice varieties on greenhouse gas emissions and b) to determine the optimum manure application rate to increase rice yield while mitigating GHG emissions under alternate wetting and drying irrigation in paddy rice production.

Materials and methods

The first pot experiment was conducted at open field, Department of Agronomy, Yezin Agricultural University (19° 45'N and 96° 6'E), Myanmar, during the wet season (June – October), 2016 to study the local production potential of GHG emission in this area. A two-factor factorial experiment with completely randomized design was used with 3 replications. The factor A was assigned into two categories of organic manure (compost and cow dung) and control (no manure). The compost is collected from straw compost making process and

stored for ten months. The cow dung is resulted from farmer traditional heap method for ten months. The amount of organic manure for cow dung and compost treatments was based on the nitrogen content of the organic manure analysis. The recommended chemical fertilizer amounts are 60 kg N ha^{-1}, 30 kg P_2O_5 ha^{-1}, and 20 kg K_2O ha^{-1} for all treatments. The recommended amounts of nitrogen fertilizer of compost and cow dung treatments were replaced by compost and cow dung manures which was based on same amount of carbon content. Therefore, 3 t ha^{-1} of compost was applied to compost treatment, and 3.15 ton cow dung + chemical fertilizer (22.15 kg N ha^{-1}) was applied to cow dung treatment to get the recommended amount of nitrogen fertilizer based on their nitrogen analysis. The control treatment received only recommended amount of chemical nitrogen fertilizer. The factor B was two types of rice varieties: Manawthukha (135 days) and IR-50 (115 days). The two rice varieties were grown in a concrete pot (52.5 cm in diameter and 45 cm in height). The soil was collected from a lowland irrigated rice field. Twenty-one-day-old seedlings were transplanted with two seedlings per pot. Compost and cow dung manures were broadcasted at 14 days before transplanting to avoid transplanting shock due to the manure decomposition process. The soil was analysed for pH (1:5 soil: water suspension), electrical conductivity (1:5 soil: water suspension), total N% (Kjaldehl distillation method), organic matter% (Tyurin's method), calcium chloride extractable SO_4-S (Turbidity method) and Texture (pipette method). The manures were analysed for total N% (Kjaldehl distillation method), total P% (Molybivanado phosphoric acid method), total K% (Wet digestion with HNO3: HCLO4 (4:1), total S% (Turbidity method) and organic carbon % (Tyurin's method). Table 1 shows the physiochemical properties of the soil and manures. The recommended amount of T-super (30 kg P_2O_5 ha^{-1}) was applied as basal fertilizer, and the recommended amount of potash (20 kg K_2O ha^{-1}) was applied with two split applications (as basal fertilizer and at the panicle initiation stage) to all treated pots. The water level was maintained at 5 cm throughout

Commented [T5]: I think this will be understandable for treatment detail.

Commented [T6]: Soil detection method

the rice growing period except during the drying period before harvest. During the rice growing season, weather data were recorded at the Department of Agronomy and are shown in Fig 1. The average minimum and maximum temperatures during the rice growing season (wet season) were 23.9 and 31.8°C, respectively, with 739 mm of rainfall.

The second pot experiment was conducted in a farmer's field, Madaya Township (22° 13' 0" N and 96° 7' 0" E), Myanmar, during the dry and wet seasons (February – October 2017) to assess the effect of cow dung manure and water management on greenhouse gases emissions from paddy rice soils. The pots were arranged in a two-factor factorial experiment with completely randomized design with three replications. Water management (continuous flooding (CF) and alternate wetting and drying (AWD) was arranged as factor A. Different rates of organic manure were assigned as factor B. In this study, cow dung manure was applied as an organic source based on the reduced global warming potential (GWP) value in previous study and widely used in the study area. The cow dung manure treatments (OM_0 = no cow dung, OM_1 = half of the recommended cow dung (2.5 t ha^{-1}), OM_2 = the recommended rate of cow dung (5 t ha^{-1}) and OM_3 = one and a half times the recommended rate of cow dung (7.5 t ha^{-1}), were applied seven days before transplanting. The recommended rate of cow dung manure is 5 t ha^{-1}. Each pot received the recommended fertilizer at the rates of 90 kg N ha^{-1}, 30 kg P_2O_5 ha^{-1}, and 20 kg K_2O ha^{-1}. Urea, T-super and potash were used as nutrient sources. Urea was applied as three equal split applications at the active tillering, panicle initiation and heading growth stages. T-super was applied only as a basal fertilizer at one day before transplanting, and potash fertilizer was applied in two equal split applications as a basal fertilizer and at panicle initiation. The soil was collected from a lowland irrigated rice field and analysed for pH (1:5 soil: water suspension), available N (Alkaline permanganate method), available P (9C-Olsen’s P-Malachite green), available K (1N Ammonium acetate extraction), total N% (Kjaldehl distillation method), organic matter%

(Tyurin's method), cation exchange capacity (CEC) (Leaching method) and texture (Pipette method). The cow dung manure was collected from farmer traditional heap method stored for ten months, and analysed for total N% (Kjaldehl distillation method) and organic carbon % (Tyurin's method). Table 2 shows the physiochemical properties of the soil and cow dung.

Commented [T7]: Soil detection method

IR-50 rice variety (115 days) was used based on reduced emission in previous study and widely grown in the study area. Dry-season rice was transplanted on 1st February 2017 and harvested on 14 May 2017. Wet-season rice was transplanted on 8 July 2017 and harvested on 11st October 2017. Just after transplanting, a base (40 cm in diameter with 2.5 cm water seal, 5 cm in height) was placed around the plants used for gas sampling to avoid disturbing the environmental conditions around the rice plants during chamber deployment in both experiments. The base was equipped with a water seal to ensure a gas-tight closure. The base remained embedded in the soil throughout the rice growing period. Water tubes (PVC pipe-25 cm height with six row holes each 2.5 cm apart) were installed in the AWD pots at a depth of 15 cm below the soil surface between the seedlings and the base just after transplanting. For AWD pots, whenever there was no water in the water tube, irrigation water was applied to a 5 cm depth above the soil surface. The irrigation interval ranged from 4 to 9 days, and the amount ranged from 7 to 13 litres depending on the different rates of cow dung manure in the AWD pots. Withdrawal of water was started one week before the harvest period in all irrigated pots. During the rice growing seasons, weather data were collected from the Department of Agricultural Research, Madaya and are shown in Fig 2. The average minimum and maximum temperatures were 21.8°C and 35.6°C during the dry season and 26.8°C and 35.7°C during the wet season, respectively. The total rainfall amounts were 201.7 mm during the dry season and 420.6 mm during the wet season.

Table 1. Physiochemical properties of experimental soil and organic manures used in the first pot experiment

Item	Total N%	Total P%	Total K%	O.C%
Cow dung	1.2	1.0	2.1	23.3
Compost	2.0	2.9	1.9	24.5

Item	pH	EC (dS/m)	Total N%	OM%	SO4-S (mg/kg)	Texture %		
						Sand	Silt	Clay
Soil	6.7	0.4	0.2	2.7	12	79.74	13.28	6.98

Table 2. Physiochemical properties of experimental soil and cow dung manure used in the second pot experiment

Analytical Item	Unit	Analytical Result	
Soil pH		7.4	Moderately alkaline
Available N	mg kg^{-1}	50	Low
Available P	mg kg^{-1}	13	Medium
Available K	mg kg^{-1}	78	Low
Total N	%	0.17	
Organic matter	%	1.8	Low
CEC	$cmol_c$ kg^{-1}	11	Low
Sand	%	87	
Silt	%	4	
Clay	%	9	
Textural class			Loamy sand
**Cow dung manure**		**Dry season**	**Wet season**
Total N	%	1.32	1.2
Organic carbon	%	16	23.3

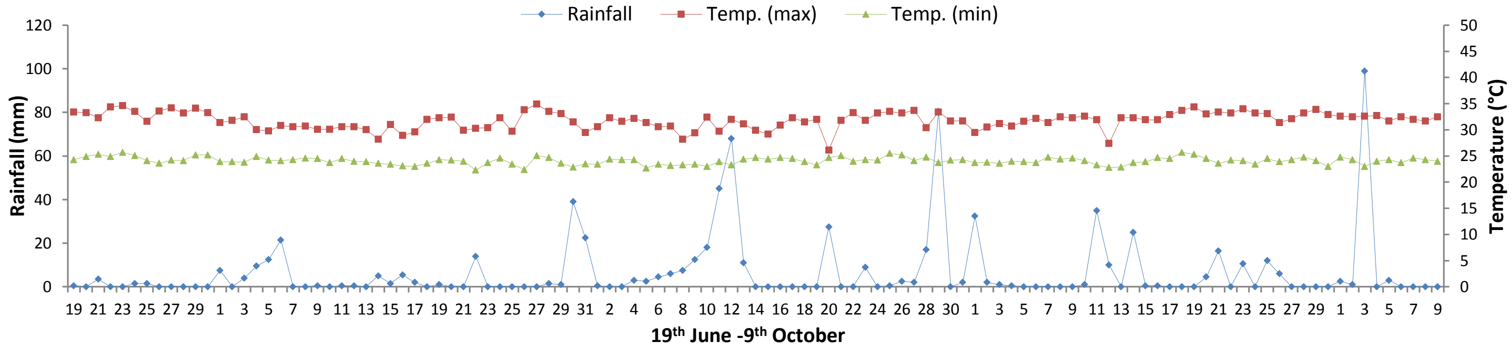

Fig 1. Daily rainfall, maximum and minimum temperatures in Yezin Agricultural University, Myanmar during wet season, 2016

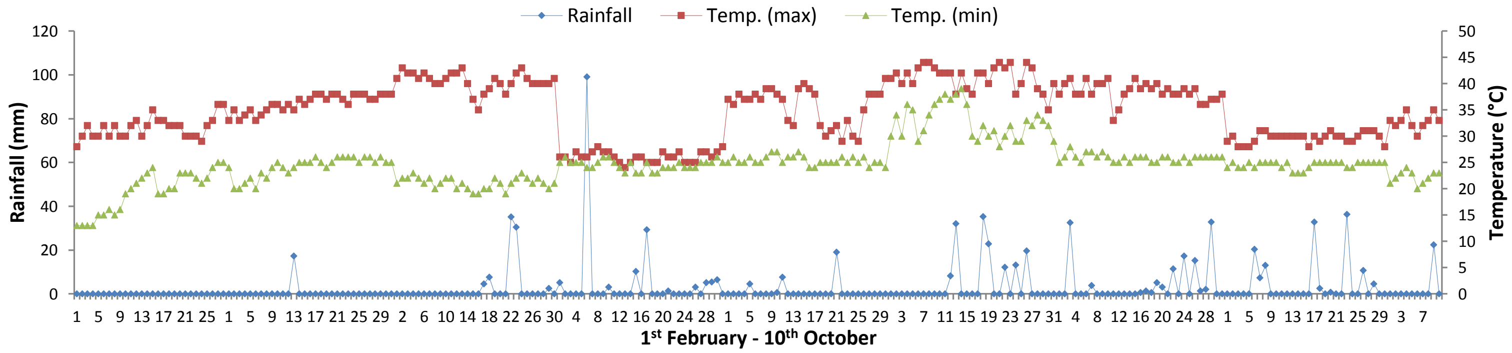

Fig 2. Daily rainfall, maximum and minimum temperatures in Madaya township, Myanmar during dry and wet seasons, 2017

Gas sample collection, analysis and calculation

A two-bonded chamber with a total capacity of 77 L (93 cm height) was used for collecting the gas sample. To thoroughly mix the gases in the chamber, the chamber was equipped with a small 12 volt DC fan connected with three 9-volt dry battery [43]. For CH_4 calculation, the temperature was recorded with a digital thermometer (TT-508 Tanita, Tokyo, Japan). To compensate for the air pressure changes between the increased temperature and gas sampling, an air buffer bag (1-L Tedlar bag) was attached to the chamber. The silicon rubber tube connected with three-way stop cock was inserted airtight into a hole on the chamber. The gas sample was taken with an airtight 50 ml syringe by connecting it to the three-way stop cock and then transferred to a 20 ml pre-evacuated glass vial.

Gas sampling was performed at 7-day intervals starting from 1 day after transplanting until harvest by the closed chamber method in pot experiment 1. In pot experiment 2, the first two gas samplings were performed at one-week intervals after transplanting, and later gas samplings were performed at 10-day intervals. The gas samples were collected from 9:00 am until 12:00 am and three times (0, 15, 30 min) for each treatment for gas flux calculation.

Methane and N_2O concentrations were analysed with a gas chromatograph (GC 2014, Shimadzu Corporation, Kyoto, Japan) equipped with a flame ionization detector (FID) and an electron capture detector (ECD). The amount of CH_4 and N_2O fluxes was calculated by using the following equation:

$$Q = (V/A) \times (\Delta c/\Delta t) \times (M/22.4) \times (273/K)$$

where Q = the flux of gas (mg m^{-2} min^{-1})

V = the volume of the chamber (m^3)

A = the base area of the chamber (m^2)

($\Delta c/\Delta t$) = the rate of increase or decrease in the gas concentration (mg m^{-3}) per unit time (min)

M = the molar weight of the gas

K = Kelvin temperature of the air temperature inside the chamber

Total emissions were calculated by interpolation method of sample gas analysis at each gas measurement for the growing period. In this study, the IPCC factors were used to calculate the combined GWPs for 100 years (GWP= (25×CH_4) + (298×N_2O)) in kg CO_2-equivalents ha^{-1} [9] for CH_4 and N_2O. The yield was recorded from each pot. The grains were threshed, cleaned and sun-dried. Yields were adjusted at 14% moisture by using the following formula to remove the error due to the different moisture content, and grain moistures were measured by using a grain moisture metre (model: GMK-303RS).

Adjusted grain weight at 14% moisture level = A x W

where A = Adjustment coefficient

W = Weight of harvested grains

$$A= \frac{100\text{-moisture\%}}{86}$$

In both water management practices, the water was applied with 1.2 L water cup. The total amount of water applied throughout the growing season was recorded and water saving in AWD was calculated.

The greenhouse gas intensity (GHGI) was calculated by dividing the GWP by the rice grain yield [44-46].

GHGI = GWP/grain yield (kg CO_2-eq. kg^{-1} grain)

Statistical analysis

The data were analysed by using Statistix (version 8.0). Mean comparisons were performed by least significant difference (LSD) test at the 5% level.

Results

First pot experiment during the wet season, 2016

Methane emission: During the early growth stage, low CH_4 emission was observed for both varieties until 36 days after transplanting (DAT), and then CH_4 emission flux gradually increased with some fluctuations until harvest (Fig 3). The mean cumulative CH_4 emissions from the control, compost, and cow dung treatments were 0.893, 0.951 and 0.808 g CH_4 kg^{-1} soil, respectively. Although the effect was not significant, cow dung amendment reduced cumulative CH_4 emissions by 9.5% compared with the control and 15% compared to compost. When comparing the two rice varieties, the total cumulative CH_4 emissions were significantly higher in the Manawthukha variety (1.084 g CH_4 kg^{-1} soil) than in the IR-50 variety (0.683 g CH_4 kg^{-1} soil) ($P<0.0046$) (Table 3). The IR-50 variety reduced cumulative CH_4 emissions by 37% compared to the Manawthukha variety. During the rice growing season, cumulative CH_4 emissions were higher in later growth stages (reproductive and ripening) than in the vegetative growth stage.

Nitrous oxide emission: High nitrous oxide emission was observed during very early growth until 15 DAT in both varieties. After that, a small amount of N_2O emission was found until harvest (Fig 4). Although there was no significant difference in N_2O emissions among the manure treatments, higher emission (3.218 mg N_2O kg^{-1} soil) was recorded from the control (no manure) compared to the compost (2.491 mg N_2O kg^{-1} soil) and cow dung (2.135 mg N_2O kg^{-1} soil) (Table 1). Cow dung manure reduced cumulative N_2O emissions by 33.7% compared with the control and 14.3% compared to compost. There was also no significant difference in cumulative N_2O emissions among the tested varieties: Manawthukha variety (2.585 mg N_2O kg^{-1} soil), IR-50 (2.644 mg N_2O kg^{-1} soil) (Table 3).

Global warming potential (GWP): GWP was not significantly different among manure treatments, although higher GWP (17.2 Mt CO_2-eq. ha^{-1}) was observed in the compost treatment followed by the control (16.3 Mt CO_2-eq. ha^{-1}) and cow dung treatment (14.6 Mt CO_2-eq. ha^{-1}) (Fig 5a). There was a significant difference in GWP among the varieties ($P<0.0050$). A higher GWP was observed for Manawthukha (19.5 Mt CO_2-eq.ha^{-1}) than for IR-50 (12.5 Mt CO_2-eq. ha^{-1}).

Rice yield: Grain yield was not significantly affected by the manure treatments. However, the numerically highest grain yield (122.3±2.4 g $plant^{-1}$) was recorded from the control (no manure), followed by the compost (102.6±13.6 g $plant^{-1}$) and cow dung treatments (79.6±15.6 g $plant^{-1}$). There was significant different in grain yield between the varieties Manawthukha (121.5±15.0 g $plant^{-1}$) and IR-50 (81.6±8.0 g $plant^{-1}$) ($P<0.0164$) (Table 3).

Greenhouse gas intensity: Greenhouse gas intensity was not affected by manure management and varieties (Table 3). However, across manure management treatments, higher GHGI values were found in Manawthukha (3.8 kg CO_2-eq. kg^{-1} grain) than in IR-50 (3.5 kg CO_2-eq. kg^{-1} grain) (Fig 5b). There was no interaction between manure and rice varieties on the GHGI.

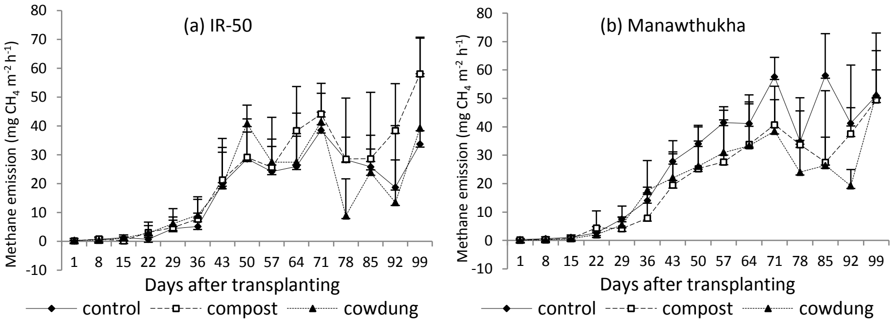

Fig 3. Methane emission of rice varieties at Yezin Agricultural University during the wet season, 2016. Mean value±standard deviation (n=3).

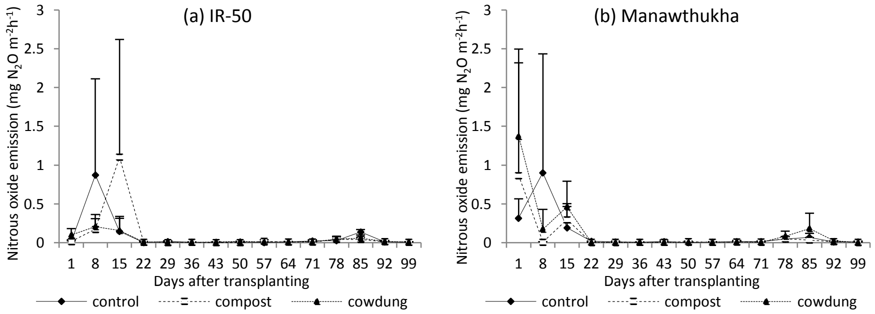

Fig 4. Nitrous oxide emission of rice varieties at Yezin Agricultural University during the wet season, 2016. Mean value±standard deviation (n=3).

Table 3. Effects of manure and rice variety on greenhouse gases emission and grain yield of rice during wet season, 2016

Treatment	CH_4 (g kg^{-1} soil)	N_2O (mg kg^{-1} soil)	Grain yield (g plant^{-1})	GHGI (kg CO_2-eqv kg^{-1} grain)
Manure				
Control	0.893	3.218	122.3± 2.4 a	2.8
Compost	0.951	2.491	102.6±13.6 ab	3.8
Cow dung	0.808	2.135	79.6±15.6 b	4.3
LSD $_{0.05}$	0.307	3.490	38.2	1.7
Variety				
Manawthukha	1.084 a	2.585	121.5±15.0 a	3.8
IR-50	0.683 b	2.644	81.6±8.0 b	3.5
LSD $_{0.05}$	0.251	2.849	31.2	1.4
Pr>F				
Manure	0.6091	0.7922	0.0901	0.2332
Variety	0.0046	0.9649	0.0164	0.6719
Manure*Variety	0.6724	0.5766	0.6633	0.9931
CV (%)	27.67	106.09	29.92	38.66

Within each column, values with different alphabets indicate significant differences among the treatments at 5% of LSD test.

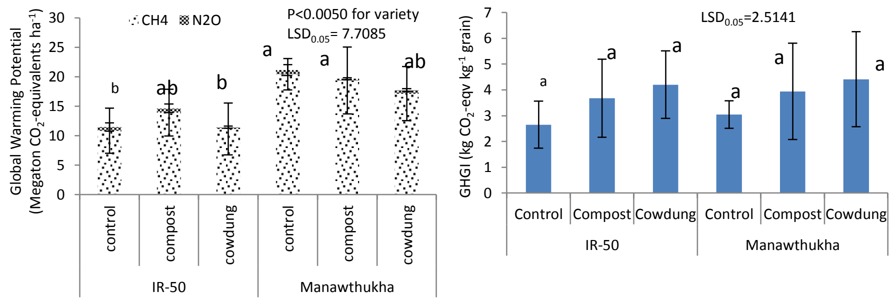

(a) Global warming potential (b) Greenhouse gas intensity

Fig 5. Effect of organic manure and rice varieties on (a) global warming potential and (b) greenhouse gas intensity during wet season, 2016. Mean value±standard deviation (n=3).

Second pot experiment during the dry and wet seasons, 2017

***Methane emission**:* The seasonal methane emissions of rice are shown in Fig 6 (dry season) and Fig 7 (wet season). In the dry season, high methane emissions were observed in the early growth stage and then decreased until harvest under both water regimes. In the wet season, a slight increase in emissions was recorded in the early growth stage, emissions peaked in the middle stage, and gradually decreased until harvest. There were significant differences in cumulative CH_4 emissions among water management practices ($P<0.0003$) (Table 4). Higher cumulative CH_4 emissions were observed under CF than AWD. Despite no significant difference among the cow dung manure rates, a generally higher amount of cow dung manure produced more methane emissions than lower rates (Fig 12a).

***Nitrous oxide emission**:* The seasonal nitrous oxide emissions of rice are shown in Fig 8 (dry season) and Fig 9 (wet season). Relatively high nitrous oxide emissions were found in the early growth stage, and reduced emissions were found in the later growth stage in both seasons. There were significant different in nitrous oxide emissions among the water management practices in dry season ($P<0.0159$) but not significant in wet season (Table 4). AWD gave a relative high N_2O emission in both seasons. No significant difference was observed in N_2O emission among the manure rates in either season.

***Global warming potential (GWP)**:* The GWP was significantly different among water management practices in both seasons ($P<0.0004$, $P<0.0000$). A higher GWPs (66.3 Mt CO_2-eq. ha^{-1} and 40.0 Mt CO_2-eq. ha^{-1}) were observed under CF than under AWD (20.1 Mt CO_2-eq. ha^{-1} and 13.7 Mt CO_2-eq. ha^{-1}) in the dry (Fig 10a) and wet seasons (Fig 10b), respectively. Generally, the large application of cow dung manure resulted in a higher GWP in both seasons (Fig 12b). The different rates of cow dung manure had no significant effect on the GWP in either season.

***Rice yield**:* In this pot experiment, the grain yield was not significantly affected by water management and manure amendment in either rice season (Table 5). However, higher grain yields per plant were recorded with AWD than CF in both seasons. The manure rates had no significant effect on rice yield.

***Greenhouse gas intensity**:* Greenhouse gas intensities were significantly different among the water management practices in both seasons (P<0.0002, P<0.0000) (Table 5). Significantly higher GHGI values were found under CF than under AWD in both seasons (Fig 11). No significant differences were found in GHGI values among the manure management practices. However, the higher amount of cow dung manure affected the GHGI values under CF irrigation, but the effect of manure was suppressed by AWD.

***Water input and water saving**:* Water inputs of rice as affected by water and manure management are shown in Table 6. There was significantly different of water input among the water management practices in either rice season (P<0.0001, P<0.0000). CF was irrigated more than AWD. The organic manure increases the water holding capacity of the soil. Accordingly, the higher amount of cow dung manure used less water. Water saving of treatments is shown in Table 7. AWD saved water 13.6% over CF in no cow dung manure in dry season and 19.1% in wet season. Across the manure management, the water saving of rice mainly depends on water management practices.

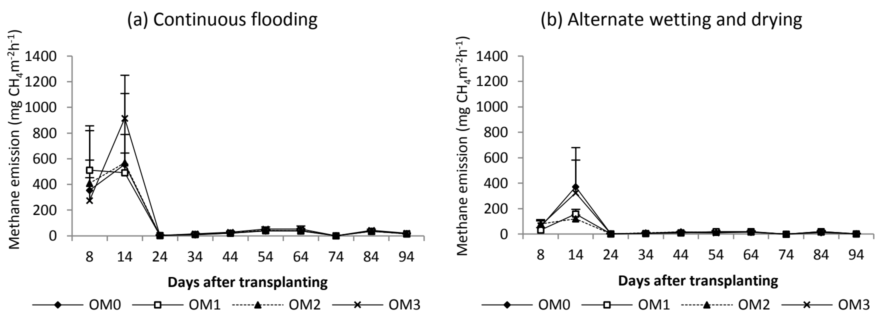

Fig 6. Methane emission from rice under (a) continuous flooding and (b) alternate wetting and drying during the dry season, 2017. Mean value±standard deviation (n=3).

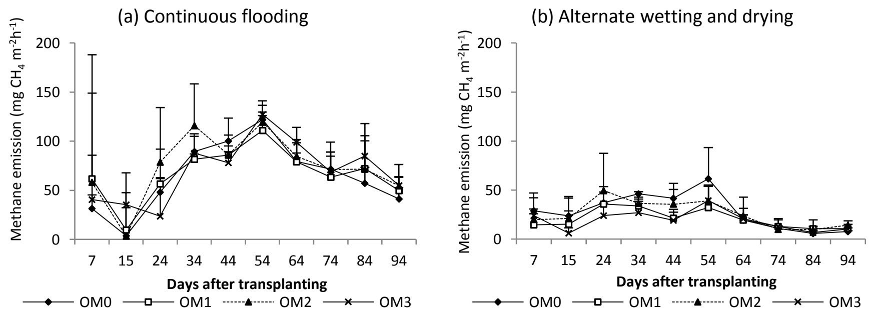

Fig 7. Methane emission from rice under (a) continuous flooding and (b) alternate wetting and drying during the wet season, 2017. Mean value±standard deviation (n=3).

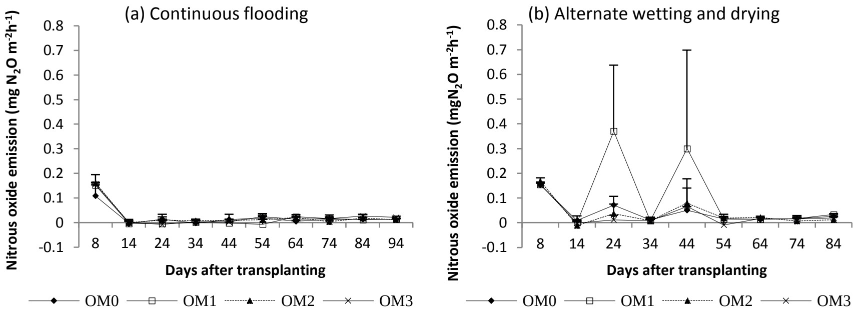

Fig 8. Nitrous oxide emission from rice under (a) continuous flooding and (b) alternate wetting and drying during the dry season, 2017. Mean value±standard deviation (n=3).

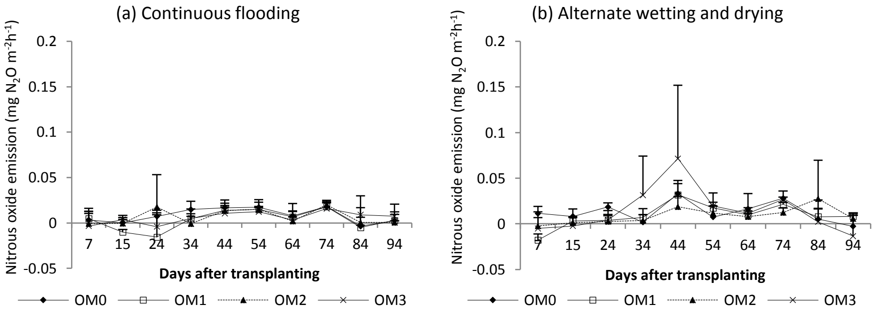

Fig 9. Nitrous oxide emission of rice under (a) continuous flooding and (b) alternate wetting and drying during the wet season, 2017. Mean value±standard deviation (n=3).

Table 4. Mean effects of water and manure management on greenhouse gases emission of rice during dry and wet seasons, 2017

Treatment	Cumulative methane emission ($g\ CH_4\ kg^{-1}$ soil)		Cumulative nitrous oxide emission ($mg\ N_2O\ kg^{-1}$ soil)	
	Dry season	Wet season	Dry season	Wet season
Water				
CF	3.327 a	2.009 a	0.590 b	0.183
AWD	0.996 b	0.682 b	1.392 a	0.344
LSD $_{0.05}$	1.089	0.369	0.631	0.176
Manure				
OM_0 (0 t ha^{-1})	2.129	1.361	0.786	0.306
OM_1 (2.5 t ha^{-1})	2.036	1.279	1.621	0.201
OM_2 (5 t ha^{-1})	2.007	1.460	0.817	0.234
OM_3 (7.5 t ha^{-1})	2.475	1.283	0.740	0.312
LSD $_{0.05}$	1.540	0.522	0.892	0.248
Pr>F				
Water	0.0003	0.0000	0.0159	0.0709
Manure	0.9114	0.8682	0.1546	0.7335
Water x Manure	0.9038	0.8217	0.0890	0.8582
CV (%)	58.21	31.74	73.53	77.08

Within each column, values with different alphabets indicate significant differences among the treatments at 5% of LSD test.

(a) Dry season (b) Wet season

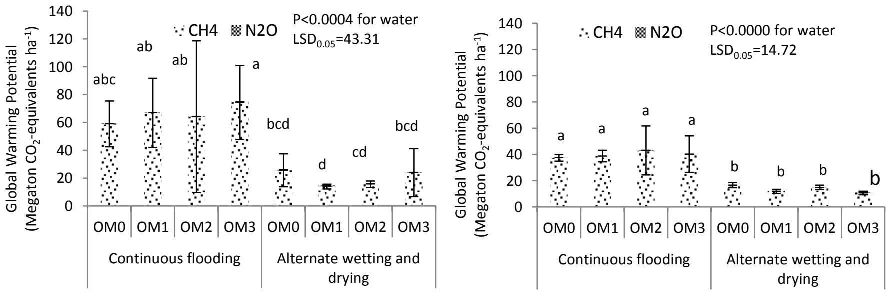

Fig 10. Effect of water and cow dung manure management on global warming potential of potted rice during dry (a) and wet (b) seasons, 2017. OM_0-no cow dung, OM_1- cow dung 2.5 t ha^{-1}, OM_2- cow dung 5.0 t ha^{-1}, OM_3- cow dung 7.5 t ha^{-1}. Mean value±standard deviation (n=3).

Table 5. Mean effects of water and manure management on rice yield during dry and wet seasons, 2017

Treatment	Yield (g plant^{-1})		GHGI (kg CO_2-eq. kg^{-1} grain)	
	Dry season	Wet season	Dry season	Wet season
Water				
CF	175.06±6.3	180.88±4.9	7.4 a	4.3 a
AWD	177.73±6.8	183.47±6.7	2.1 b	1.4 b
LSD $_{0.05}$	12.67	14.27	2.3	0.7
Manure				
OM_0 (0 t ha^{-1})	181.54±9.4	183.70±3.1	4.5	2.9
OM_1 (2.5 t ha^{-1})	171.79±3.2	178.81±0.1	4.7	2.7
OM_2 (5 t ha^{-1})	178.08±11.0	179.20±0.1	4.3	3.1
OM_3 (7.5 t ha^{-1})	174.16±5.0	187.01±7.6	5.6	2.7
LSD $_{0.05}$	17.92	20.18	3.2	1.1
Pr>F				
Water	0.6608	0.7060	0.0002	0.0000
Manure	0.6758	0.7984	0.8359	0.8283
Water x Manure	0.3976	0.6511	0.7742	0.8092
CV (%)	8.30	9.05	55.13	31.13

Within each column, values with different alphabets indicate significant differences among the treatments at 5% of LSD test.

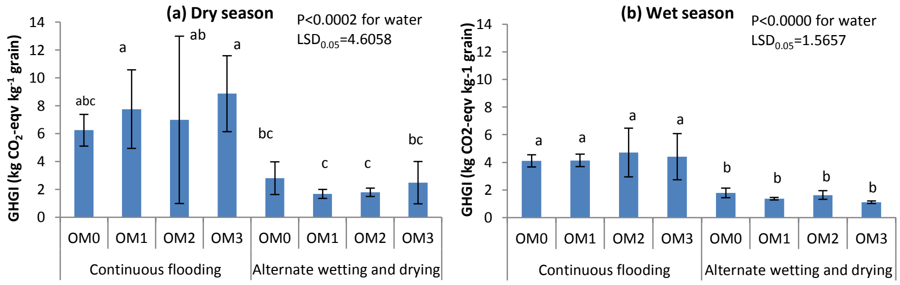

Fig 11. Effect of water and cow dung manure management on greenhouse gas intensity of potted rice during dry (a) and wet (b) seasons, 2017. OM_0-no cow dung, OM_1-cow dung 2.5 t ha^{-1}, OM_2- cow dung 5.0 t ha^{-1}, OM_3- cow dung 7.5 t ha^{-1}. Mean value±standard deviation (n=3).

Table 6. Mean effects of water and manure management on water input of rice during dry and wet seasons, 2017

Treatment	Water input $_{(I+R)}$ (mm)	
	Dry season	Wet season
Water		
CF	1075.5 a	842.1 a
AWD	969.8 b	681.4 b
LSD $_{0.05}$	42.4	32.7
Manure		
OM_0	1030.5	775.0
OM_1	1017.6	750.5
OM_2	1015.4	740.6
OM_3	1027.1	780.9
LSD $_{0.05}$	60.0	46.2
Pr>F		
Water	0.0001	0.0000
Manure	0.9385	0.2372
Water x Manure	0.0955	0.7619
CV (%)	4.80	4.96

Within each column, values with different alphabets indicate significant differences among the treatments at 5% of LSD test.

Table 7. Comparison of water saving of rice as affected by water and cow dung manure management during dry and wet seasons, 2017

Treatment		Water saving (%)	
Water	Cow dung manure	Dry season	Wet season
CF	OM_0		
CF	OM_1		
CF	OM_2		
CF	OM_3		
AWD	OM_0	13.6	19.1
AWD	OM_1	7.5	18.7
AWD	OM_2	2.8	17.1
AWD	OM_3	14.9	21.2

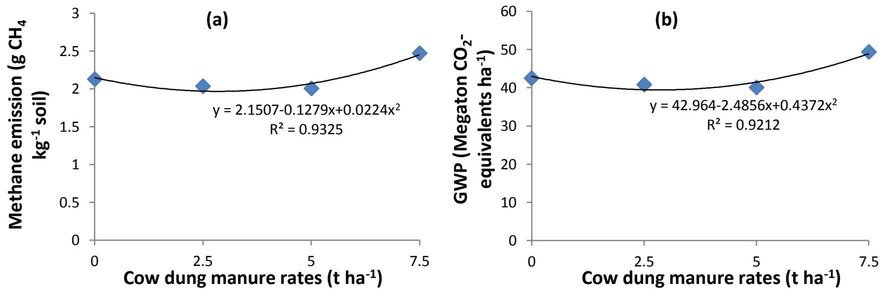

Fig 12. Relationship between methane emission and different cow dung manure rates (a) and between GWP and different cow dung manure rates (b) in dry season, 2017

Discussion

Effect of manure and rice varieties on greenhouse gas emissions, global warming potential and rice yield

Methane production and emissions from flooded paddies are highly affected by the addition of organic matter [47]. The higher CH_4 emission in this study was found in later growth stages (Fig 3) because the CH_4 emission was associated with higher soil organic matter turnover due to increased microbial activities, decomposition of decaying plant residues from fallen leaves and root turnover, and higher availability of root exudates in the rhizosphere [48]. Although there was no significant difference based on manure management, cow dung manure reduced methane emissions. This study also agrees with that of Oo et al. [30], who reported the lower CH_4 emissions from pots treated with well-decomposed cattle manure due to fewer carbon substrates with a depletion of potential CH_4 precursors resulting from the preceding fermentation. In contrast to CH_4 emissions, high N_2O emissions were recorded in the early growth stage, and small amounts of emissions were recorded in the later growth stage (Fig 4). This could be due to the rapid nitrification with the presence of oxygen and denitrification with the utilization of NO_3^- as electron acceptor in the initial stage with high temperature and low rainfall (Fig 1), and indigenous soil nitrogen (Table 1). After emission peaked initially, the rates of N_2O emission were generally low due to continuous flooding. Another study reported that the consistently low soil redox potential under continuous flooding resulted in more complete denitrification and thus reduced N_2O emissions [49]. The control (no manure) treatment resulted in higher N_2O emissions than the compost and cow dung treatments. Lower nitrous oxide emission was resulted by incorporation of organic inputs due to nitrogen immobilization [50-52]. This study agrees with that of Shan and Yan [53], who reported that N_2O emissions were significantly reduced

Commented [T8]: I revised it according to your suggestion.

by crop residue return combined with synthetic N fertilizers compared with emissions from treatments only received synthetic N fertilizer.

The selection of suitable rice varieties might play a significant role in regulating CH_4 emissions from rice fields [54]. The results of this study showed that there was a significant difference between the tested rice varieties (Fig 3 and Table 3). The result was in agreement with other findings, which highlighted that there were substantial differences in the rates of CH_4 emission among different rice varieties [30, 55, 56]. As ninety percent of methane emissions to the atmosphere are through rice plants [57], the Manawthukha variety, which has a longer growth duration, emitted more CH_4 than IR-50, which has a shorter growth duration. Previous studies [42, 58-60] have also reported that the CH_4 flux from late maturing rice is higher than that from early maturing rice. According to this research finding, a shorter growth duration (IR-50) variety can be used to reduce methane emissions. When compared to the two rice varieties, IR-50 resulted in higher N_2O emissions than the Manawthukha variety. This could be due to the favourable effect of root exudates of IR-50 on the nitrification process in the soil. Gogoi and Baruah [25] reported that the main driving forces influencing N_2O emission in rice were soil NO_3-N, soil organic carbon. There was no interaction between manure and rice varieties. The compost and Manawthukha variety emitted higher CH_4 due to longer decomposing time and support of substrates for methanogens. The control treatment and IR-50 variety resulted in higher N_2O emission due to nitrification and denitrification of inorganic nutrient in the favour of root exudates (Fig 5a).

Cow dung manure resulted in a 10.4% reduction in GWP compared to the control (no manure) with reduced CH_4 and N_2O emission. Combination of decomposed cow dung manure + mineral fertilizer might suppress the available carbon and nitrogen for CH_4 and N_2O production. Unfortunately, in our study, we couldn’t measure the carbon and nitrogen at every gas sampling and their mechanisms. While an increased GWP (5.5%) was found in the

compost compared to the control. Huang et al. [61] reported that incorporation of organic residues provides a source of readily available C and N in the soil and subsequently influences N_2O emissions. Manawthukha had a higher GWP (56%) than IR-50. These results were supported by Zheng et al. [62], who reported that yield-scaled GWP at 80-90 days of growth duration after transplanting was 87% higher than that at 70-80 days of growth duration after transplanting. Feng et al. [63] also reported that yield-scaled GWP in late rice in a double-rice cropping system was 73% higher than that for varieties with 90-100 days of growth duration after transplanting.

Commented [T9]: This is the explanation of how manure affect on GHG emission.

A high grain yield of rice was recorded from the control due to the mineralization of indigenous soil organic matter and inorganic fertilizer (Table 3). Oo et al. [64] reported a high grain yield under inorganic sources of nutrients due to the immediate release and availability of nutrients. Relative to IR-50, Manawthukha had a higher grain yield by 48.9% whereas the GWP was reduced in IR-50 than in Manawthukha. Therefore, considering the mitigation practice for GWP from rice production, IR-50 and cow dung manure was experimented in 2017 rice seasons.

Effect of water management and manure application on greenhouse gas emissions, global warming potential and rice yield

In the dry season, high methane emissions were observed in the early growth stage and then decreased until harvest in both water regimes (Fig 6). The early increase in CH_4 emission was due to the indigenous soil carbon content and availability of substrates, and the decrease in the later growth stage was due to the senescence of older leaves and non-availability of substrate as the crop approached maturity [65-67]. In the wet season, a slight increase in emission was recorded in the early growth stage, and emission was highest in the

middle stage and gradually decreased until harvest (Fig 7). A higher rate of CH_4 production is attributed to the availability of organic substrates from the previous crop residues in the form of plant-derived C through processes such as root exudation and release of fallen leaves and intensively reduced conditions in the rice rhizosphere [54, 68]. The gradual decrease in CH_4 emission in later growth stage was due to the decomposition and non-availability of substrates depending on temperature (Fig 2). Higher cumulative CH_4 emissions were observed under CF than under AWD in both seasons because the anoxic conditions increased the methanogen population and favoured methane production. The methane emission was higher in the dry season than in the wet season because it depended on the availability of indigenous soil carbon and decomposition process favoured by high temperature in the dry season, and depletion of soil carbon and previous crop resides decomposition hindered by low temperature in wet season (Fig 2). Relatively high nitrous oxide emissions were observed in the early growth stage, and reduced emissions were observed in the later growth stage in both seasons (Fig 8 and 9). This was due to the indigenous soil nitrogen content in the early stage, and depletion of nutrients and the available nitrogen content for nitrification and denitrification in the later stage. Higher N_2O emissions were observed under AWD than CF in both seasons [54, 69, 70]. AWD increased N_2O emissions by 135% compared to CF in dry season and 88% in wet season. Similar to the CH_4 emission pattern, higher N_2O emissions were observed in the dry season than in the wet season because of the soil nitrogen availability and the favoured soil condition (high temperature and low rainfall) for nitrification and denitrification in the dry season [71, 72].

Generally higher rates of cow dung manure resulted in higher CH_4 and N_2O emission because it provided carbon and nitrogen sources for methanogenesis and nitrification and denitrification process. Methane is produced by methanogenic bacteria during the anaerobic digestion of organic substrates [4] and N_2O production is observed by soil water content and

availability of substrates (nitrate and easily degradable organic matter) for denitrification [73]. However, the polynomial distribution of methane emission with different cow dung manure rates (Fig 12a) and polynomial distribution of GWP with different cow dung manure rates (Fig 12b) were observed in dry season. The polynomial regression equation for methane emission is $y = 2.1507-0.1279x+0.0224x^2$. The equation showed that the methane emission decreased in the rate of 0.1279 g CH_4 kg^{-1} soil at every increased unit (ton) of cow dung manure applied, after that increased in the rate of 0.0224 g CH_4 kg^{-1} soil at every increased unit (ton) of cow dung manure applied. The coefficient of determination (R^2) showed that 93% of variation in methane emission could be accounted by the quadratic regression equation of different cow dung manure rates. The polynomial regression equation for GWP is $y = 42.964-2.4856x+0.4372x^2$. The equation showed that the GWP decreased in the rate of 2.4856 Mt CO_2-equivalent ha^{-1} at every increased unit (ton) of cow dung manure applied, after that increased in the rate of 0.4372 Mt CO_2-equivalent ha^{-1} at every increased unit (ton) of cow dung manure applied. The coefficient of determination (R^2) showed that 92% of variation in GWP could be accounted by the quadratic regression equation of different cow dung manure rates. According to these results, it would be recommended that 5 t ha^{-1} should be applied for improving soil fertility and reduced greenhouse gas emission. No interactive effect between water management and the application of different cow dung manure rates was found on CH_4 emissions in either season. However, the effect of the application of different cow dung manure rates on N_2O emissions was influenced by alternate wetting and drying irrigation practices in the dry season but not in the wet season. Therefore, the nitrification and denitrification processes of soil are influenced by the soil moisture content [74-77].

Commented [T10]: This is the explanation of rates of manure on emission according to our findings.

A higher GWP was observed under CF in accordance with the higher methane emissions in both seasons (Fig 10). Methane emission mainly contributes to the GWP from

paddy production. Many studies [39, 54, 78-82] reported that N_2O emissions contribute much less to the global warming potential than those of CH_4. Therefore, the water regime in paddy production is the main factor controlling CH_4 emissions from rice fields [39, 54, 83].

AWD resulted in a higher grain yield per plant than did CF in both seasons (Table 3.3) because it strengthened the air exchange between the soil and atmosphere and supplied sufficient oxygen to the root system to accelerate soil organic matter mineralization, all of which should increase soil fertility and favour rice growth [84, 85]. Yang and Zhang [86] reported an increase in paddy yield under AWD due to the increase in the proportion of productive tillers. In this study, AWD saved water about 10% over CF in dry season and 19% in wet season. Zhang et al. [87] also indicated an irrigation water saving of 35% under AWD with a 10% yield increase relative to that under CF. Liu et al. [88], Ye et al. [89], and Djaman et al. [90] found that grain yield increased with reduced water input by AWD. Grain yield was not affected by the different rates of cow dung manure application because its decomposition was influenced by biotic and soil-environmental factors. Relative to the CF water management, AWD produced comparable grain yields, increased by 1.5% and decreased GHGI by 69% (Table 5). This suggests that by adopting alternate wetting and drying irrigation, it would be possible to achieve the dual goals of maintaining productivity while minimizing the global warming potential of rice cultivation.

Conclusion

For sustainable agriculture, organic manures should be added at a recommended amount to improve the rice yield and reduce greenhouse gas emissions. From our findings, the application of cow dung manure can be recommended in paddy production since it mitigated the global warming potential compared to that of the control and compost groups, although it resulted in a lower yield potential. Additionally, the short duration rice variety had a lower GWP and a lower GHGI value while maintaining the potential rice yield. Manure-

Commented [T11]: I mainly consider this based on reduced emission and sustainable production.

induced greenhouse gas emissions were suppressed by AWD irrigation practices, with significant GHGI values. Thus, short duration varieties are highly recommended with AWD irrigation and application of 5 t ha^{-1} cow dung manure to reduce greenhouse gas emissions and maintain rice yield under the soil-environmental conditions of Myanmar. In our study, there was no relationship between GWP and rice yield. Therefore, the choice of rice varieties should be combined with soil-environmental factors and cultivation systems to mitigate greenhouse gas emissions while increasing rice yields for sustainable rice production. Further studies under field condition are needed to measure the effect of manure and mineral fertilizer on greenhouse gas emission, global warming potential and rice yield under water management conditions for better understanding of emission mechanisms.

Acknowledgements

We thank Sonoko D. Bellingrath-Kimura and Aung Zaw Oo for technical advice and editing a previous manuscript version.

Reference

1. Burney JA, Davis SJ, Lobell DB. Greenhouse gas mitigation by agricultural intensification. Proc. Nat. Acad. Sci. U. S. A. 2010; 107: 12052–12057.
2. Tilman D, Balzer C, Hill J, Befort BL. Global food demand and the sustainable intensification of agriculture. Proc. Nat. Acad. Sci. USA. 2011; 108: 20260–20264.
3. Smith P, Martino D, Cai Z, Gwary D, Janzen H, Kumar P, et al. Agriculture. In: Metz, B., Davidson, O.R., Bosch, P.R., Dave, R., Meyer, L.A. (Eds.), Climate Change 2007: Mitigation. Contribution of Working Group III to the Fourth Assessment Report of the Intergovernmental Panel on Climate Change. Cambridge University Press, Cambridge, UK and New York, NY, USA, 2007; 497–540.
4. Mer JL and Roger P. Production, oxidation, emission and consumption of methane by soils: A review. Eur. J. Soil Biol. 2001; 37: 25-50.
5. Yan X, Akiyama H, Yagi K, Akimoto H. Global estimations of the inventory and mitigation potential of methane emissions from rice cultivation conducted using the

2006 Intergovernmental Panel on Climate Change Guidelines. Global Biogeochem. Cyc. 2009; 23, GB2002.

6. Zou J, Huang Y, Qin Y, Liu S, Shen Q, Pan G, et al. Changes in fertilizer-induced direct N2O emissions from paddy fields during ricegrowing season in China between 1950 and 1990. Global Change Biol., 2009; 15: 229–242.
7. Wang JY, Jia JX, Xiong ZQ, Khalil MAK, Xing GX. Water regime-nitrogen fertilizer-straw incorporation interaction: field study on nitrous oxide emissions from a rice agroecosystem in Nanjing. China. Agric. Ecosyst. Environ., 2011; 141: 437–446.
8. Meijide A, Manca G, Goded I, Magliulo V, di Tommasi P, Seufert G, et al. Seasonal trends and environmental controls of methane emissions in a rice paddy field in Northern Italy. Biogeosciences, 2011; 8: 3809-3821.
9. IPCC. Climate change 2007: synthesis report *Contribution of Working Groups I, II and III to the Fourth Assessment Report of the Intergovernmental Panel on Climate Change*, ed. By Pachauri, RK and Reisinger A. IPCC, Geneva, Switzerland, 2007; 104 p.
10. Forster P, Ramaswamy V, Artaxo P, Berntsen T, Betts R, Fahey DW, et al. Changes in atmospheric constituents and in radiative forcing. In: Solomon S, Qin D, Man-ning M, Chen Z, Marquis M, Averyt K, Tignor M, Miller H (Eds) Climate Change 2007: the physical science basis. Contribution of Working Group I to the Fourth Assessment Report of the Intergovernmental Panel on Climate Change. Cambridge University Press: UK and New York pp 129–234.
11. IPCC: Intergovernment Panel on Climate Change. 2013. Fifth Assessment Report (AR5) Contribution of Working Group III to Climate Change 2014: Mitigation of Climate Change.
12. Ma Y, Wang J, Zhou W, Yan X, Xiong Z. Greenhouse gas emissions during the seedling stage of rice agriculture as affected by cultivar type and crop density. Biol. Fertil. Soils 2012; 48: 589-595.
13. Win EP, Win KK, Bellingrath-Kimura SD, Oo AZ. Greenhouse gas emissions, grain yield and water productivity: a paddy rice field case study based in Myanmar. Greenhouse Gases: Science and Technology, 2020; 10: 884-897.
14. Liu S, Zhang Y, Lin F, Zhang L, Zou J. Methane and nitrous oxide emissions from direct-seeded and seedling-transplanted rice paddies in southeast China. Plant Soil, 2013. Available: http://dx.doi.org/10.1007/s11104-013-1878-7.

15. Ma J, Ji Y, Zhang G, Xu H, Yagi K. Timing of midseason aeration to reduce CH_4 and N_2O emissions from double rice cultivation in China. Soil Sci. Plant Nutr., 2013; 59: 35-45.
16. Das K. and Baruah K K. Association between contrasting methane emissions of two rice (*Oryza sativa* L.) cultivars from the irrigated agroecosystem of northeast India and their growth and photosynthetic characteristics. Acta Physiologiae Plantarum, 2008; 30: 569-578.
17. Setyanto P, Rosenani AB, Boer R, Fauziah CI and Khanif MJ. The effect of rice cultivars on methane emission from irrigated rice field. Indonesian J Agric Sci., 2004; 5: 20-31.
18. Eller G, Frenzel P. Changes in activity and community structure of methane oxidizing bacteria over the growth period of rice. Appl Environ Microbiol, 2001; 67: 2395–2403.
19. Dubey SK, Singh JS. Spatio-temporal variation and effect of urea fertilization on methanotrophs in a tropical dryland rice field. Soil Biol Biochem, 2000; 32: 221–226.
20. Ma KE, Qiu Q, Lu Y. Microbial mechanism for rice variety control on methane emission from rice field soil. Global Change Biol. 2010. doi:10.1111/j13652486200902145
21. Wang W, Lai DYF, Wang C, Tong C, Zeng C. Effects of inorganic amendments, rice cultivars and cultivation methods on greenhouse gas emissions and rice productivity in a subtropical paddy field. Ecological Engineering 2016; 95: 770-778.
22. Ciarlo E, Conti M, Bartoloni N, Rubio G. The effect of moisture on nitrous oxide emissions from soil and the $N_2O/(N_2O+N_2)$ ratio under laboratory conditions. Biol. Fertil. Soils 2007; 43: 675-681.
23. Liou SM, Lo SL, Wang SH. A generalized water quality index for Taiwan. Environmental Monitoring and Assessment, 2003; 96: 35-52.
24. Chen J, Qin G, Wang J, Yu J, Shen B, Li S, et al. One-step fabrication of sub-10-nm plasmonic nanogaps for reliable SERS sensing of microorganisms. Biosens Bioelectron, 2013; 44: 191-197.
25. Gogoi B and Baruah KK. Nitrous oxide emissions from fields with different wheat and rice varieties. Pedosphere 2012; 22(1): 112-121.
26. Mariko S, Harazono Y, Owa N, Nouchi I. Methane in flooded soil water and the emission through rice plants to the atmosphere. Environ Exp Bot., 1991; 31: 343–350.

27. Wang B, Neue HU, Samonte HP. Effect of cultivar difference (‘IR72’, ‘IR65598’ and ‘Dular’) on methane emission. Agric Ecosyst Environ., 1997; 62: 31–40.
28. Xu YC, Wang ZY, Li Z, Wang BJ. Effect of rice cultivars on methame emission from Beijing rice field. Plant Nutr Fert Sci., 1999; 5: 93–96 (in Chinese with English abstract).
29. Aulakh MS, Bodenbender J, Wassmann R, Rennenberg H. Methane transport capacity of rice plants, II. Variations among different rice cultivars and relationship with morphological characteristics. NutrCycling Agroecosyst. 2000; 58: 367–375.
30. Oo AZ, Win KT, Motobayashi T and Bellingrath-Kimura SD. Effect of cattle manure amendment and rice cultivars on methane emission from paddy rice soil under continuously flooded conditions. J. Environmental Biology, 2016; 37(5): 1029-1036.
31. Singh S, Kumar S, Jain MC. Methane emission from two Indian soils planted with different rice cultivars. Biol Fert Soils. 1997; 25: 285–289.
32. Khosa MK, Sidhu BS, Benbi DK. Effect of organic materials and rice cultivars on methane emission from rice field. J Environ Biol., 2010; 31: 281–285.
33. Yu J, Wang LL, Yan XJ, Tian YL, Deng AX, Zhang WJ. Super rice cropping will enhance rice yield and reduce CH4 emission: a case study in Nanjing. Chin Rice Sci., 2013; 20: 427–433.
34. Qin XB, Li YE, Wan YF, Liao YL, Fan MR, Gao QZ, et al. Effect of tillage and rice residue return on CH4 and N2O emission from double rice field. Trans Chin Soc Agric Eng., 2014; 30: 216–224 (in Chinese with English abstract).
35. Kongchum M. Effect of Plant Residue and Water Management Practices on Soil Redox Chemistry, Methane Emission, and Rice Productivity. A Dissertation, he Graduate Faculty of the Louisiana State University and Agricultural and Mechanical College, 2005.
36. Jan PL, Gerard LV, Wim de V, Johannes K. Differentiation of nitrous oxide emission factors for agricultural soils. Environmental Pollution, 2011; 159: 3215-3222.
37. Adhya TK, Bharati K, Mohanty SR, Ramakrishnan B, Rao VR, Sethunathan N, et al. Methane emission from rice fields at Cuttack, India. Nutrient Cycling in Agroecosystems, 2000; 58: 95-105.
38. Gathorne-Hardy A. A Life Cycle Assessment (LCA) of greenhouse gas emissions from SRI and flooded rice production in Southeast India, Taiwan Water Conservancy Journal, 2013; 61(4): 111-125.

Commented [T12]: I have confirmed it.

39. Yan X, Yagi K, Akiyama H, Akimoto H. Statistical analysis of the major variables controlling methane emission from rice fields. Global Change Biol., 2005; 11(7): 1131–1141.
40. Alfred GH, Duv NR, Venkatanarayana M, Barbara HW. A Life Cycle Assessment (LCA) of greenhouse gas emissions from SRI and flooded rice production in SE India. Taiwan Water Conservancy, 2013; 61(4): 110-125.
41. Myanmar Agriculture Sector in Brief. Crop Sub-Sector. Department of planning, the Ministry of agriculture, livestock and irrigation, Nay Pyi Taw, Myanmar, 2019.
42. Oo AZ, Win KT, Bellingrath-Kimura SD. Within field spatial variation in methane emissions from lowland rice in Myanmar. Springer Plus. 2015; 4: 145. Available: http://dx.doi.org/10.1186/s40064-015-0901-2.
43. Ali MA, Kim PJ and Inubushi K. Mitigating yield-scaled greenhouse gas emissions through combined application of soil amendments: A comparative study between temperate and subtropical rice paddy soils. Science of the Total Environment, 2015;529: 140–148.
44. Xiong Z, Yinglie L, Wu Z, Huang T. Differences in net global warming potential and greenhouse gas intensity between major rice-based cropping systems in China. Scientific Report 2015; 5: 17774.
45. Pittelkow CM, Adviento-Borbe MA, Kessel C, Hill JE, Linquist BA. Optimizing rice yields while minimizing yield-scaled global warming potential. Global Change Biol. 2014; 20: 1382–1393.
46. Van Groenigen JW, Velthof GL, Oenema O, Van Groenigen KJ, Van Kessel C. Towards an agronomic assessment of N2O emissions: a case study for arable crops. Eur. J. Soil Sci. 2010; 61: 903–913.
47. Jean LM and Pierre R. Production, oxidation, emission and consumption of methane by soils: A review. Eur. J. Soil Biol., 2001; 37: 25-50.
48. Oo AZ, Nguyen L, Win KT, Cadisch G and Bellingrath-Kimura SD. Toposequential variation in methane emissions from double-cropping paddy rice in Northwest Vietnam. Geoderma, 2013; 209-210, 41-49.
49. Oo AZ, Sudo S, Inubushi K, Mano M, Yamamoto A, Ono K, et al. Methane and nitrous oxide emission from conventional and modified rice cultivation systems in South India. Agriculture, Ecosystems and Environment, 2018; 252: 148-158.

50. Zou J, Huang Y, Jiang J, Zheng X, Sass RL. A 3-year field measurement of methane and nitrous oxide emissions from rice paddies in China: Effects of water regime, crop residue, and fertilizer application. Global Biogeochem. Cycles, 2005; 19: GB2021.
51. Dalal RC, Gibson I, Allen DE, Menzies NW. Green waste compost reduces nitrous oxide emissions from feedlot manure applied to soil. Agric. Ecosyst. Environ. 2010; 136: 273-281.
52. Iqbal J, Parkin TB, Helmers MJ, Zhou X, Castellano MJ. Denitrification and nitrous oxide emissions in annual croplands, perennialgrass buffers, and restored perennial grasslands. Soil Sci. Soc. Am. J. 2015; 79: 239-250.
53. Shan J and Yan X. Effects of crop residue returning on nitrous oxide emissions in agricultural soils. Atmospheric Environment, 2013; 71: 170-175.
54. Oo AZ, Sudo S, Inubushi K, Chellappan U, Yamamoto A, Ono K, et al. Mitigation Potential and Yield-Scaled Global Warming Potential of Early-Season Drainage from a Rice Paddy in Tamil Nadu, India. Agronomy 2018; 8: 202; doi:10.3390/agronomy8100202
55. Kumar JIN and Viyol S. Short term diurnal and temporal measurement of methane emission in relation to organic carbon, phosphate and sulphate content of two rice fields of central Gujarat, India. Journal of Environmental Biology, 2009; 30(2): 241-246.
56. Koga N. and Tajima R. Assessing energy efficiencies and greenhouse gas emissions under bioethanol-oriented paddy rice production in northern Japan. Journal of Environmental Management, 2011; 92(3): 967-973.
57. Wang ZY, Xu YC, Li Z, Guo YX, Wassmann R, Neue N, et al. A four year record of methane emission from irrigated rice fields in Beijing region of China, Nutr Cycling Agroecosys, 2000; 58: 55-63.
58. Cai ZC, Tsuruta H, Minami K. Methane emission from rice fields in China: measurements and influencing factors. J. Geophys. Res. 2000; 105 (D13): 17231–17242.
59. Satyanto P, Makarim AK, Fagi AM, Wassmann R and Buendia LV. Crop management affecting methane emissions from irrigated and rainfed rice in Central Jawa (Indonesia), Nutr Cycling Agroecosys, 2000; 58: 85-93.
60. Yang X, Shang Q, Wu P, Liu J, Shen Q, Guo S, et al. Methane emissions from double rice agriculture under long-term fertilizing systems in Hunan, China. Agric. Ecosyst. Environ. 2010; 137: 308–316.

61. Huang Y, Zou J, Zheng X, Wang Y. and Xu X. Nitrous oxide emissions as influenced by amendment of plant residues with different C/N ratios. Soil Biology and Biochemistry, 2004; 36: 973-981.
62. Zheng H, Huang H, Yao L, Liu J, He H, Tang J. Impacts of rice varieties and management on yield-scaled greenhouse gas emissions from rice fields in China: A meta-analysis. Biogeosciences, 2014; 11: 3685-3693.
63. Feng JF, Chen CQ, Zhang Y, Song ZW, Deng AX, Zheng CY, et al. Impacts of cropping practices on yield-scaled greenhouse gas emissions from rice fields in China: a meta-analysis, Agr. Ecosyst. Environ., 2013; 164: 220–228.
64. Oo AN, Banterng P, Polthanee A and Trelo-Ges V. The Effect of Different Fertilizers Management Strategies on Growth and Yield of Upland Black Glutinous Rice and Soil Property. Asian Journal of Plant Sciences, 2010; 9: 414-422.
65. Das S, Adhya TK. Effect of combine applica-tion of organic manure and inorganic fertilizer on me-thane and nitrous oxide emissions from a tropical flooded soil planted to rice. Geoderma, 2014; 213: 185-192.
66. Borah L, Baruah KK. Effects of foliar application of plant growth hormone on methane emission from tropical rice paddy. Agriculture Ecosystems and Environment 2016; 233:75–84.
67. Baruah KK, Gorh D, Gogoi N. Analysis of greenhouse gas (Methane and Nitrous oxide) emission and global warming potential from rice fields: with reference to biological mitigation of climate change. SDRP Journal of Earth Sciences & Environmental Studies, 2018; 3(2): 1-13.
68. Kell DB. Large-scale sequestration of atmos-pheric carbon via plant roots in natural and agricultural ecosystems: why and how. Philos. Trans. R. Soc. B. 2012; 367:1589–1597.
69. Hou AX, Chen GX, Wang ZP, Van Cleemput O, Patrick Jr, WH. Methane and nitrous oxide emissions from a rice field in relation to soil redox and microbiological process. Soil Sci. Soc. Am. J. 2000; 64: 2180–2186.
70. Jain N, Dubey R, Dubey DS, Singh J, Khanna M, Pathak H, et al. Mitigation of greenhouse gas emission with system of rice intensification in the Indo-Gangetic Plains. Paddy Water Environ., 2014; 12(3): 355–363.
71. Dalal RC, Wang WJ, Robertson GP, Parton WJ. Nitrous oxide emission from Australian agricultural lands and mitigation options: A review. Aust. J. Soil Res., 2003; 41: 165–195.

Commented [T13]: I have confirmed this.

72. IPCC (Intergovernmental Panel on Climate Change). Guidelines for National Greenhouse Gas Inventories. In National Greenhouse Gas Inventories Programme; Eggleston HS, Buendia L, Miwa K, Ngara T, Tanabe K, Eds.; IGES: Hayama, Kanagawa, Japan, 2006.
73. Dobbie KE, McTaggart IP and Smith KA. Nitrous oxide emissions from intensive agricultural systems: variations between crops and seasons, key driving variables, and mean emission factors. J. Geophys. Res. 1999; 104: 26891–26899.
74. Simojoki A and Jaakkola A. Effect of nitrogen fertilization, cropping and irrigation on soil air composition and nitrous oxide emission in a loamy clay. Eur. J. Soil Sci. 2000; 51: 413–424.
75. Dobbie KE and Smith KA. The effects of temperature, water-filled pore space and land use on N2O emissions from an imperfectly drained gleysol. Eur. J. Soil Sci. 2001; 52: 667–673.
76. Simojoki A. Oxygen supply to plant roots in cultivated mineral soils. Pro Terra No. 7, Helsinki, Finland, 2001; 59 pp. Dissertation, pdf version available at http://ethesis.helsinki.fi .
77. Skiba U and Ball B. The effect of soil texture and soil drainage on emissions of nitric oxide and nitrous oxide. Soil Use Manage. 2002; 18: 56–60.
78. Kurosawa M, Yamashiki Y, Tezuka T. Estimation of energy consumption and environmental impact reduction by introducing sustainable agriculture to rice paddy field cultivation. J. Life Cycle Ass. Japan 2007; 3(4): 232–238.
79. Itoh M, Sudo S, Mori S, Saito H, Yoshida T, Shiratori Y, et al. Mitigation of methane emissions from paddy fields by prolonging midseason drainage. Agric. Ecosyst. Environ., 2011; 141: 359–372.
80. Pittelkow CM, Adviento-Borbe MA, Hill JE, Six J, van Kassel C, Linquist B. Yield-scaled global warming potential of annual nitrous oxide and methane emissions from continuously flooded rice in response to nitrogen input. Agric. Ecosyst. Environ., 2013; 177: 10–20.
81. Sander BO, Wassmann R, Siopongco JDLC. Mitigating greenhouse gas emissions from rice production through water-saving techniques: potential, adoption and empirical evidence. In: Hoanh, C., Smakhtin, V., Johnston, R. (Eds.), Climate Change and Agricultural Water Management in Developing Countries. CAB International, Oxfordshire, UK, 2015; 193–207.

82. Tarlera S, Capurro MC, Irisarri P, Scavino AF, Cantou G, Roel A. Yieldscaled global warming potential of two irrigation management systems in a highly productive rice system. Sci. Agric. 2016; 2: 43–50.
83. Wu X, Wang W, Xie X, Yin C, Hou H, Yan W, et al. Net global warming potential and greenhouse gas intensity as affected by different water management strategies in Chinese double rice-cropping systems. Scientific reports, 2018; 8: 779.
84. Dong NM, Brandt KK, Sørensen J, Hung NN, Hach CV, Tan PS, et al. Effects of alternating wetting and drying versus continuous flooding on fertilizer nitrogen fate in rice fields in the Mekong Delta, Vietnam. Soil Biol. Biochem. 2012; 47: 166–174.
85. Tan X, Shao D, Liu H, Yang F, Xaio C, Yang H. Effects of alternate wetting and drying irrigation on percolation and nitrogen leaching in paddy fields. Paddy Water Env., 2013; 11: 1–15.
86. Yang JC, Zhang JH. Grain-filling problem in ‘super’ rice. J. Exp. Bot. 2010; 61: 1–5.
87. Zhang H, Xue Y, Wang Z, Yang J, Zhang J. Alternate wetting and moderate soil drying improves root and shoot growth in rice. Crop Sci. 2009; 49: 2246–2260.
88. Liu L, Chen T, Wang Z, Zhang H, Yang J, Zhang J. Combination of site-specific nitrogen management and alternate wetting and drying irrigation increases grain yield and nitrogen and water use efficiency in super rice. Field Crops Res. 2013; 154: 226–235.
89. Ye M, Song Y, Long J, Wang R, Baerson SR, Pan Z, et al. Priming of jasmonate-mediated antiherbivore defense responses in rice by silicon. Proc. Natl. Acad. Sci. USA 2013; 110: 3631-3639.
90. Djaman K, Mel VC, Diop L, Sow A, El-Namaky R, Manneh B, et al. Effects of AlternateWetting and Drying Irrigation Regime and Nitrogen Fertilizer on Yield and Nitrogen Use Efficiency of Irrigated Rice in the Sahel. Water, 2018; 10: 711.

Debjani Sihi
Academic Editor
PLOS ONE
PONE-D-20-31281] - [EMID:e10e97d637debdbb]

Dear Dr. Sihi,
Thanks for handling our manuscript "**Influence of rice varieties, organic manure and water management on greenhouse gas emissions from paddy rice soils**". We sincerely appreciate valuable and positive comments from three anonymous reviewers on the original manuscript. We have taken all the comments and suggestions into account in the revised manuscript. “Lxx–xx” in the below responses indicate the lines in the manuscript file after all the track changes approved. All the figures were checked to Preflight Analysis and Conversion Engine (PACE) digital diagnostic tool.

Sincerely yours,

Responses to the reviewer 1 (green highlight) (reviewer(1)pdf)

No.	General Comments	Responses
1	What do you mean by off-site?	Not related to farming but it related to transportation and fertilizer industry. (See L107)
2	Did the application dependent on carbon content or nitrogen content? clarify	The application depended on nitrogen content, and applied the same amount based on carbon content. (See L127-135)
3	If each plot received recommended fertilizer, what was the purpose of adding manure?	As we mentioned in the introduction, farmers use organic manure in addition to chemical fertilizer in flooded rice. To represent farmers’ field condition, we added manure in this study.
4	Did you consider how availability of different acceptors would affect CH4 production	Although we consider availability of different acceptors from different amendments would affect CH_4 emission, in this study we did not discuss detail about it.
5	Do you have data to support that cow dung was humified organic matter than others (compost)? Or this is an assumption? Also the difference was not significant.	This is our assumption and revised it. (See L490)
6	This should be a complement to your observation of low CH4 in the initial stage because nitrification occuring could mean presence of oxygen and denitrification could mean utilization of NO3 as electron	Thank you, Sir. I revised it. (See L495-497)

	acceptor, refer to the point I made above	
7	This is an assumption?	Yes, Sir but I revised it with reference. (See L519,520)
8	You can't have a paragraph that is one statement	I revised it. (See L520)
9	This was just a repetition of the results without explaining why Manure reduced GWP and composed increased GWP	I revised it. (See L526-532)
10	As I indicated in the methods section, If each plot received recommended fertilizer, what was the purpose of adding manure? Why was the nutrient in the manure not considered, does it mean manure just added extra nutriments that plant did not need for production?	I have responded it above. As we answered before, we added manure to represent farmers' practice apart from recommended chemical fertilizer. In Myanmar, the application rate of chemical fertilizer is still very low, thus, we consider that combined application will further improve crop production but we also assume that manure amendment in flooded rice might enhance CH_4 emission.<br>(See L127-135)
11	This is not consistent with your finding, in your finding it was inorganic fertilizer that recorded higher grain yield but not organic+inorganic fertilizer. This is not consistent.	Thank you very much for your thoroughly editing and kind patience. I revised it. (See L541)
12	Does the dry season affect the water level?	As I mentioned in Material and Method section, I kept the water level 5 cm in CF, and AWD when need irrigation. So there is no effect on water level because of dry season.
13	Does this contrast your statement in line 247	I revised it.
14	Is this a suggestion? or did you test for it? or do you have a citation to support it?	I revised it with citation. (See L601)
15	This study is interesting, however the design has a number of deficiencies, for example one part considers wet and dry season, the other part only considers one season. All plot received recommended fertilizer, then manure/compost was added what was the purpose of adding manure/compost. Why were the nutrients from manure/nutrients ignored? Tables reported data for C, N etc then in the methods	Thank you for constructive comments. We revised the methods, discussion by responding all comments. Please see L127-135, L141-147, L172-179.

	there is no mention of how these nutrients were analyzed. The discussion was poorly thought through and is inconsistent with series of statements that are not supported by data or literature. The discussion will need to be rewritten. I would recommend not publishing this paper in the status it is but have the author do major changes for consideration, which will include re-write abstract, discussion and include the methods missing. In addition to other suggestions below.	
16	Paragraph 1 Why did you consider wet season only, while the second study you covered both wet and dry seasons?	We added detail explanation about the seasons in the methods. See L122 and L157,158.

Responses to the reviewer 2 (gray highlight) (reviewer pdf)

No.	General Comments	Responses
1	Please write conclusion or recommendation	I revised it. Please see L31-36.
2	pot was kept in open field or in the laboratories?	Revised (open field). Please see L120
3	cow dung manure was fresh or dry	Air dry
4	either amount was calculated based on N required for application or carbon content<br><br>was whole amount of recommend N was applied through organic manure?<br><br>was fertilizer applied to control?	I have revised them. Please see L127-135
5	how soil was collected and filled in the pots ?	I collected the surface soil from lowland irrigated rice fields and pour them to pot after measuring of their weights.
6	which rice variety was used in second experiment	I revised it. Please see L180

7	OM+90 kg N ?	I revised it. Please see L127-135.
8	why same parameters have not been analyzed in both the experiment. it should be similar	Sorry, Sir for this condition. But in first pot experiment, I would like to test the organic inputs so I analyzed them for nearly complete set. However, in second pot experiment, I just wanted to know N and C content of cow dung manure for GHG emission.
9	write full dimension of the chamber (height, width and length)	Sir, I couldn't mention the full dimension of the chamber. So please may I show you the photo of chamber. See L212. 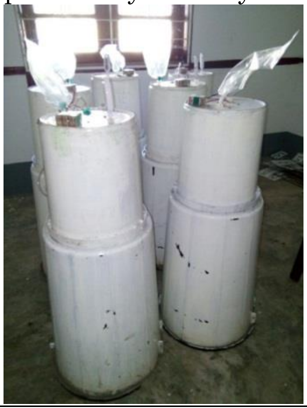
10	please mention date of harvesting so that duration of gas sampling may be clear	I revised it. Please see L181-183.
11	standard deviation for yield may be indicated	I indicated it.
12	not clear. need rewriting	I have revised it. Please see L297-299.
13	control did not received manure, therefor trend of CH4 emission might be different from manure amended soil. due to gradual decomposition of applied manure<br><br>please explain<br><br>was sampling of GHG was done up to 99 DAT for each treatment or different duration for different treatments?	Please see L262-266 for explanation of CH_4 emission from control and manure treatments.<br>Sir, I collected gas samples according to their different duration. However, the comparison was done up to final sampling of short duration variety.
14	cumulative CH4 emission seems decreasing with increase in cow dung application rate up to 5 t/ha than it increased. please discuss in details about this trend	I revised it. Please see L575-595.
15	which are more humified: cow dung manure or compost	I revised it. Please see L490.
16	need more clarification.<br>crop residue incorporation result into lower N2O emission due to immobilization.	The crop residue incorporation results into lower N_2O emission. The cow dung and compost also result into lower N_2O emission. See L502,503.

	however, cow dung and compost might led to higher N2O emission as compared to control.	
17	no data on biomass yield, number of tillage has been reported in the study. the CH4 emission is dependent on number of tillers and duration of the crop	I revised it. See L512,513.
18	Need more clarification. CH4 is result of anaerobic decomposition of OM.	I revised it. See L521-524.
19	this section need more discussion with appropriate reported justification	Sir, I revised it and please may I know more comments and suggestion.

Responses to the reviewer 3 (Yellow highlight) (reviewer 1pdf)

No.	General Comments	Responses
1	The statistical design used by the authors in this study is wrong. The split plot design is never recommended for pot study. The authors should use CRD ie complete randomized design and analyse the results again to draw the conclusions	I have revised them. Please see L122 and L158.
2	Generally the organic manure is applied based on N content and recommended dose of N and not on the basis of C content	Yes, Sir, the organic manure is applied based on N content. Sorry for misunderstood writing and I revised it. Please see L127-135.
3	My major concern is statistical design used in the experiment	I have revised it, Sir.
4	It is also not clear weather N and other fertilisers were applied in control pot or not. Specify it in the treatments detail.	N, P, K fertilizer was applied to control pot. I revised it. Please see L127-135.
5	generally potash full dose is given as basal. Is this the practice used by farmers or is it the recommendation to the farmers	It is recommendation and also practiced by farmers.
6	what does it mean.<br>Chambers were rectangular or cylindrical	‘Two-bonded’ mean binding two buckets. Sir, the chambers were neither rectangular nor cylindrical. I couldn’t say the shape of chamber. So may I show you the photo of them.

		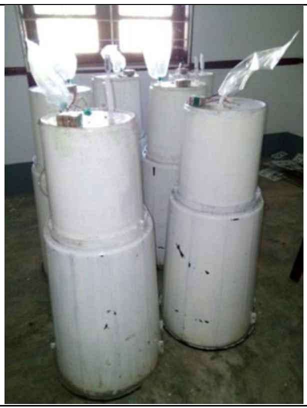
7	what was the basis for selecting the rice varieties.<br>How much soil was there in the pot.<br>Generally the emissions are high during maximum tillering stage	I chose two rice varieties for first pot experiment based on widely grown and promising in the study area. In second pot experiment, I selected IR-50 based on previous pot result and survey result in the study area. 70 kg soil/pot was used in first experiment and 79.5 kg soil/pot in second experiment.
8	I suggest the authors to revise the results based on revised statistical analysis and modified reporting of pot experiment data	I have revised them according to your comments and suggestions. Please may I know if you have more comments and suggestions on that.

Dear Sir,

I really thank you very much for your valuable and constructive comments on this manuscript. I have revised all reviewers' comments and may I know if you have more comments and suggestions to be manuscript improved.

Reviewer #1: Some statistical analysis information is missing especially in the tables but this can be corrected if the authors have data. Also more information on the analysis methods have been requested in the attached reviewer notes.

Answer: I have revised them.

Reviewer #2: Comments to the author and editors
The study is focused on impact of manure application, rice verities and water management on GHG emission from paddy soil putted in pot experiment. The objectives of this study were a) to assess the effect of different types of manure amendments and rice varieties on greenhouse gas emissions and b) to determine the optimum manure application rate to increase rice yield while mitigating GHG emissions under alternate wetting and drying irrigation in paddy rice production. I appreciate the effort of the worker for reporting data, however, the study has been conducted in the pot and data has reported for only one season which is not adequate to draw sound conclusion. It would have been more appropriate if at least two year data might have been generated. There are many research works already reported on this aspect with long duration of experiment from field trial. The manuscript also lack proper synthesis and sound discussion. It may be suitable for publication in local journal of the study area. The some comments has been made in the pdf of the manuscript and some broad comments are enlisted below

1. The readability of the manuscript is poor
2. The abstract lack genesis, objective and conclusion of the research
3. Material and methods lack: information on feed-stock of compost, clear statement regarding control and N-fertilization, intercultural operation, harvesting date, dimension of gas chamber and duration of gas sampling. Cumulative CH4 emission is highly dependent on duration of gas sampling and biomass yield especially number of tiller. No data has been presented on vegetative growth of the two rice varieties.
4. The clear difference in compost and cow dung manure has not been stated: the method of preparation, feed-stock used etc.
5. Table 2.1 and 2.2 should be similar in terms of parameters reported
6. The result section and discussion need re-synthesis to draw final conclusion. Control has shown lowest GHG intensity in first experiment, but Cow dung application has been recommended. Need proper discussion
7. In second experiment, increasing rate of cow dung has reduced CH4 emission as compared to control (control> C 2.5 t/ha > C 5 t/ha < c 7.5 t/ha) up to 5 t/ha then it increased. Need detailed discussion on this trend

Answer: Sir, thank you very much for your constructive comments and valuable usage of words. I had revised the abstract with your comments and suggestions and I substituted your comments to our manuscript to be improved of readability of it. I have revised all comments. Please may I know more comments and suggestions to be improved of our manuscript and I really thank you and appreciate your valuable comments.

Reviewer #3: The statistical design used in the not suitable for this study. The authors have to use CRD design for analysing their data. Split plot design used by authors is not for pot experiments. This type of design is used for field experiments. This is why I feed the the manuscript is not technically sound.
I suggest the authors to revise the results based on revised statistical analysis and modified reporting of pot experiment data as pot experiment result are not reported on per hectare basis

Answer: Sir, I revised the manuscript with your suggestion. I revised the statistical analysis to two factors with CRD design. Thank you very much for your constructive and valuable comments. Please may I know more comments and suggestions and I really appreciate your fruitful comments.
