# Supplementary material for: Influence of rice varieties, organic manure and water management on greenhouse gas emissions from paddy rice soils
Source: PLoS One. 2021 Jun 30;16(6):e0253755. doi: 10.1371/journal.pone.0253755 (PMC8244889; doi:10.1371/journal.pone.0253755)
Supplement: Attachment — Submitted filename: Response to Reviewers 1.docx [file pone.0253755.s005.docx]

Dear Sir,

I really thank you very much for your valuable and constructive comments on this manuscript. I have revised all reviewers’ comments and may I know if you have more comments and suggestions to be manuscript improved.

Reviewer #1: Some statistical analysis information is missing especially in the tables but this can be corrected if the authors have data. Also more information on the analysis methods have been requested in the attached reviewer notes.

Answer: I have revised them.

Reviewer #2: Comments to the author and editors
The study is focused on impact of manure application, rice verities and water management on GHG emission from paddy soil putted in pot experiment. The objectives of this study were a) to assess the effect of different types of manure amendments and rice varieties on greenhouse gas emissions and b) to determine the optimum manure application rate to increase rice yield while mitigating GHG emissions under alternate wetting and drying irrigation in paddy rice production. I appreciate the effort of the worker for reporting data, however, the study has been conducted in the pot and data has reported for only one season which is not adequate to draw sound conclusion. It would have been more appropriate if at least two year data might have been generated. There are many research works already reported on this aspect with long duration of experiment from field trial. The manuscript also lack proper synthesis and sound discussion. It may be suitable for publication in local journal of the study area. The some comments has been made in the pdf of the manuscript and some broad comments are enlisted below
1. The readability of the manuscript is poor
2. The abstract lack genesis, objective and conclusion of the research
3. Material and methods lack: information on feed-stock of compost, clear statement regarding control and N-fertilization, intercultural operation, harvesting date, dimension of gas chamber and duration of gas sampling. Cumulative CH4 emission is highly dependent on duration of gas sampling and biomass yield especially number of tiller. No data has been presented on vegetative growth of the two rice varieties.
4. The clear difference in compost and cow dung manure has not been stated: the method of preparation, feed-stock used etc.
5. Table 2.1 and 2.2 should be similar in terms of parameters reported
6. The result section and discussion need re-synthesis to draw final conclusion. Control has shown lowest GHG intensity in first experiment, but Cow dung application has been recommended. Need proper discussion
7. In second experiment, increasing rate of cow dung has reduced CH4 emission as compared to control (control> C 2.5 t/ha > C 5 t/ha < c 7.5 t/ha) up to 5 t/ha then it increased. Need detailed discussion on this trend

Answer: Sir, thank you very much for your constructive comments and valuable usage of words. I had revised the abstract with your comments and suggestions and I substituted your comments to our manuscript to be improved of readability of it. I have revised all comments. Please may I know more comments and suggestions to be improved of our manuscript and I really thank you and appreciate your valuable comments.

Reviewer #3: The statistical design used in the not suitable for this study. The authors have to use CRD design for analysing their data. Split plot design used by authors is not for pot experiments. This type of design is used for field experiments. This is why I feed the the manuscript is not technically sound.
I suggest the authors to revise the results based on revised statistical analysis and modified reporting of pot experiment data as pot experiment result are not reported on per hectare basis

Answer: Sir, I revised the manuscript with your suggestion. I revised the statistical analysis to two factors with CRD design. Thank you very much for your constructive and valuable comments. Please may I know more comments and suggestions and I really appreciate your fruitful comments.
