# Supplementary material for: Influence of rice varieties, organic manure and water management on greenhouse gas emissions from paddy rice soils
Source: PLoS One. 2021 Jun 30;16(6):e0253755. doi: 10.1371/journal.pone.0253755 (PMC8244889; doi:10.1371/journal.pone.0253755)
Supplement: Attachment — Submitted filename: Response to reviewers-R1.docx [file pone.0253755.s007.docx]

Debjani Sihi
Academic Editor
PLOS ONE

PONE-D-20-31281R1] - [EMID:5a1a391e053f9ca5]

Dear Sir,

I really thank you very much for your valuable and constructive comments and time sharing on this manuscript ‘Influence of rice varieties, organic manure and water management on greenhouse gas emissions from paddy rice soils’. We sincerely appreciate valuable and positive comments from you. We have taken all the comments and suggestions into account in the revised manuscript.

Sir, I really sorry for your time and deeply thank for that. Your comments made me improved for my profession and I got invaluable knowledge and fruitful remarks. Please may I know if you have more comments and suggestions to be manuscript improved.

Sincerely yours,

Reviewer #3:

Comment 1

Q: The authors have modified the manuscript to a larger extent. But there are several points which are not acceptable. The authors say the emission of methane in wet season is less compared to dry season.

A: Sir, these data are realistic. So I have to interpret the results according to research findings. Sir, I conducted the field experiment simultaneously with this pot experiment. In that field experiment, the emission is more in wet season than in dry season but in this pot experiment the reverse is found. Therefore, for this pot experiment, I interpreted the emission is influenced by control environmental factors and mainly depends on the soil nutritional status. (Line: 560-564)

Comment 2

Q: The addition of carbon due to application of 2.5,5 and 7.5 t of manure having 16% C will be quite different. In fact the carbon will be 2 and 3 times more in quantitative terms in OM2 and OM3. (Addition of 2.5 t of manure will add 400 kg of carbon, while OM2 and OM3 will add 800kg and 1200 kg of carbon. It is very surprising that addition of such high amount of carbon is not increasing methane emission even in wet season. It seems there is serious sampling issue. There may be some leakage issue either from the chamber or after sampling or error during analysis.

A: Sir, thank you very much for your constructive comment and it is improved to my knowledge. Yes, I was also frustrated about this finding. Therefore, according to my knowledge, I interpreted the water management is key player to emission mechanism especially CH_4_ emission nevertheless of how much addition of cowdung manure. Sir, I also agree with your suggestion about sampling issue on this point.

Comment 3

Q: Another point is at one place authors are saying manure quantity was according to N required but they adjusted to maintain same C content, not clear very mind boggling.

A: Sir, as pilot experiment, we would just like to know the effect of manures on emission. At that time, we considered that we will replace the chemical fertilizer with manures. And then, we also wanted to compare the effect of manures on emission. Therefore, we considered on same amount of Carbon of manures to get the equal chance of carbon input.

Comment 4

Q: They say High methane emission and low N2O emission from long duration variety However IR 50 is short duration but N2O is high. The justification given is not very appealing.

A: Sir, high CH4 emission and low N2O emission from long duration variety, Manawthukha.

Low CH4 emission and high N2O emission from short duration variety, IR 50.

Although CH4 emission depends on longer growth duration for methanogenesis, N2O emission depends on nutritional features of the soil for nitrification and denitrification process. Please may I know for your suggestion upon this response.

There are several other points which are given in the PDF.

Comment 5

Q: If the crop duration is more the emission of both the GHG will be more depending on the difference in crop duration

A: Sir, as my presenting in the abstract: The results showed that cumulative CH_4_ emission from Manawthukha (1.084 g CH_4_ kg^-1^ soil) was significantly higher than that from IR-50 (0.683 g CH_4_ kg^-1^ soil) (P<0.0046) with yield increase (P<0.0164) because of the longer growth duration of the former. In contrast, higher cumulative nitrous oxide emissions were found for IR-50 (2.644 mg N_2_O kg^-1^ soil) than for Manawthukha (2.585 mg N_2_O kg^-1^ soil), I got this result. Yes, I agree with your idea, the longer growth duration may give more emission of both CH_4_ and N_2_O. But Sir, according to my results, N_2_O emission mainly depends on the mineral condition in the soil. In spite of longer growth duration, nitrification and denitrification is restricted by mineral features of the soil. (Line: 15-19)

Comment 6

Q: This level of significance (P<0.0000)

A: Sir, in data analysis, this value was shown in results. (Line: 33)

Comment 7

Q: devoted

A: already changed to ‘under’ (Line: 114)

Comment 8

Q: not clear what does this mean. The C content of compost and cow dung will be different.

In the reply to reviewer comments the authors have mentioned that manure was applied in addition to recommended dose of fertiliser N.

A: Sir, first we considered to replace the recommended chemical fertilizer with manure based on N analysis of manure. Then to compare the effect of manures on emission, we considered carbon content of manures to get the same amount of carbon input for emission (Line: 131). Sir, if this reply is still frustrated, I will send you the calculation of manure based on chemical analysis. (Line: 132-134)

Comment 9

Q: Although the carbon added in OM3 was maximum (3 times the OM1) however the CH4 emission is at par with OM1 in wet season, which is very surprising. (Table 4)

A: This is good question because I was frustrated for it and thank you very much for your suggestion. Please see on comment 2.

Comment 10

Q: Fail to understand this as short duration variety will have impact on N2O also

A: Sir, according to my knowledge, the plant structure and plant type will influence the nitrification and denitrification process in case of oxygen transport and weathering of dead tissues. Unfortunately, I couldn’t measure about them. (Line: 516)

Comment 11

Q: Root exudates generally have organic acids which has impact on CH4 emission

A: Yes, Sir. It has impact on CH4 but also on N2O. I cited the reference about that fact. (Line: 518, 519)

Comment 12

Q: This not acceptable reason

A: Please see on comment 1. This data is realistic. (Line: 560-564)
